# Recycling of the actin monomer pool limits the lifetime of network turnover

**DOI:** 10.15252/embj.2022112717

**Published:** 2023-03-13

**Authors:** Alexandra Colin, Tommi Kotila, Christophe Guérin, Magali Orhant‐Prioux, Benoit Vianay, Alex Mogilner, Pekka Lappalainen, Manuel Théry, Laurent Blanchoin

**Affiliations:** ^1^ CytoMorpho Lab, Laboratoire de Physiologie Cellulaire & Végétale, Interdisciplinary Research Institute of Grenoble University of Grenoble‐Alpes, CEA, CNRS, INRA Grenoble France; ^2^ Institute of Biotechnology and Helsinki Institute of Life Science University of Helsinki Helsinki Finland; ^3^ CytoMorpho Lab, Institut de Recherche Saint Louis, U976 Human Immunology Pathophysiology Immunotherapy (HIPI) University of Paris, INSERM, CEA Paris France; ^4^ Courant Institute of Mathematical Sciences New York University New York NY USA; ^5^ Department of Biology New York University New York NY USA

**Keywords:** actin turnover, aging, lifetime, microwells, reconstituted system, Cell Adhesion, Polarity & Cytoskeleton

## Abstract

Intracellular organization is largely mediated by actin turnover. Cellular actin networks continuously assemble and disassemble, while maintaining their overall appearance. This behavior, called “dynamic steady state,” allows cells to sense and adapt to their environment. However, how structural stability can be maintained during the constant turnover of a limited actin monomer pool is poorly understood. To answer this question, we developed an experimental system where polystyrene beads are propelled by an actin comet in a microwell containing a limited amount of components. We used the speed and the size of the actin comet tails to evaluate the system's monomer consumption and its lifetime. We established the relative contribution of actin assembly, disassembly, and recycling for a bead movement over tens of hours. Recycling mediated by cyclase‐associated protein (CAP) is the key step in allowing the reuse of monomers for multiple assembly cycles. ATP supply and protein aging are also factors that limit the lifetime of actin turnover. This work reveals the balancing mechanism for long‐term network assembly with a limited amount of building blocks.

## Graphical abstract

Reconstitution of actin in a dynamic steady state using cell‐sized compartments reveals the parameters influencing long‐term assembly upon limited component availability.

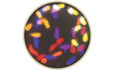

## Introduction

**Figure 1:**
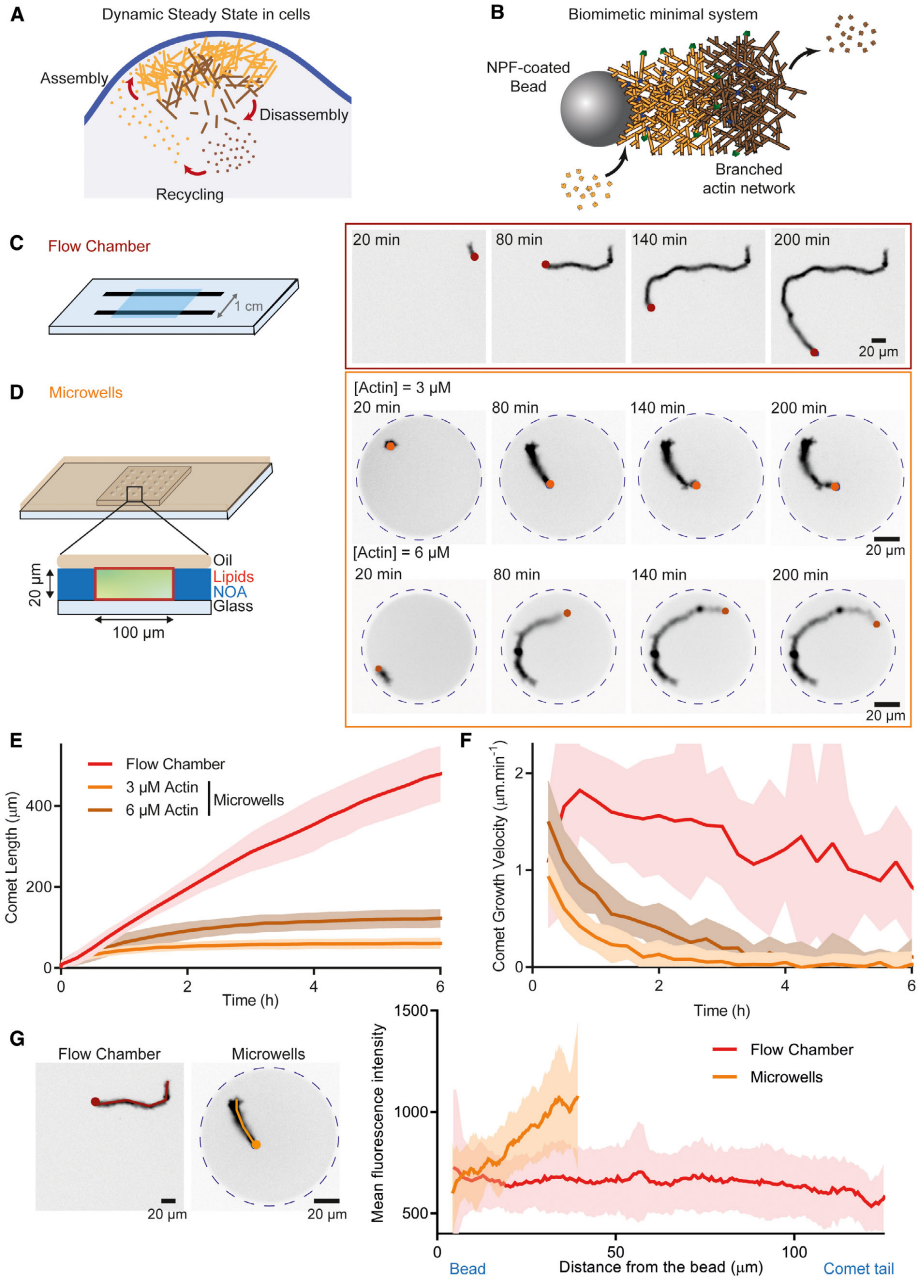
Effect of a limited pool of monomers on actin assembly Cartoon of the three‐step cycle for actin dynamic steady state in cells.Schematic of a bead coated with a nucleation‐promoting factor (NPF) of the Arp2/3 complex able to generate a branched actin comet. Actin assembly takes place on the bead. Disassembly occurs within the tail of the comet. Actin filament in orange represents the freshly assembled ATP or ADP‐Pi actin network, whereas actin in brown represents the ADP‐actin network.Left: Scheme of the flow chamber (unlimited amount of proteins) used in this study. Right: Snapshots of the growth of one actin comet from a bead (red dot) in a flow chamber.Left: Schematic of the microwells used in this study. Right: Snapshots of the growth of one actin comet from a bead (orange) in a microwell for two different initial actin monomers concentration (3 and 6 μM).Length of comets grown as a function of time in flow chamber and in microwells.Growth velocity of comets grown in flow chamber and in microwells as a function of time. Flow chamber: *N* = 2, *n* = 33 comets tails. Microwells 3 μM actin: *N* = 3, *n* = 38 comets tails. Microwells 6 μM actin: *N* = 1, *n* = 17 comet tails.Mean intensity profiles along comets grown in flow chambers and in microwells (Flow chamber: *N* = 1, *n* = 15 comets tails. Microwells 3 μM actin: *N* = 1, *n* = 21 comets tails). Data information: 4.5 μm beads coated with 400 nM of SNAP‐Strep‐WA‐His. Reaction mix: [Actin] = 3 or 6 μM. [Profilin] = 6 or 12 μM. [Arp2/3 complex] = 90 nM. [Capping Protein] = 15 nM.

Living organisms depend on maintaining a ceaseless renewal of their internal organization in the face of constant environmental changes. This energy‐dependent process can be observed at several hierarchical levels, where the stability of a biological component is supported by the turnover of its building blocks (Rafelski & Marshall, 2008; Chan & Marshall, 2012; Goehring & Hyman, 2012). In eukaryotes, the actin cytoskeleton plays many key roles in maintaining dynamic intracellular organization (Chhabra & Higgs, 2007; Lappalainen *et al*, 2022). For example, actin dynamics in the cortex (Fritzsche *et al*, 2013, 2016), lamellipodium (Pollard & Borisy, 2003) or stress fibers (Hotulainen & Lappalainen, 2006; Tojkander *et al*, 2015; Nishimura *et al*, 2021) underpin cell migration (Lai *et al*, 2008; Burnette *et al*, 2011; Rottner & Stradal, 2011; Blanchoin *et al*, 2014), and organelle dynamics (Chakrabarti *et al*, 2021). During these processes, actin filament networks constantly assemble and disassemble, often while maintaining an apparent stable structure necessary for force generation (Blanchoin *et al*, 2014; Lappalainen *et al*, 2022), suggesting that the two processes are precisely balanced. Such behaviors are called “dynamic steady‐states,” conferring upon cytoskeletal networks a high degree of plasticity that allows cells to adapt and optimize their architecture in response to external changes (Rafelski & Theriot, 2004; Lomakin *et al*, 2015; Vargas *et al*, 2016; Mueller *et al*, 2017; Banerjee *et al*, 2019). A three‐step cycle of assembly, disassembly, and recycling (Fig 1A) regulates actin dynamics in cells, which is sustained by constant energy consumption. In Step 1, actin monomers loaded with ATP assemble into actin filaments. Prior to disassembly, actin subunits in an actin filament hydrolyze their bound ATP and dissociate the inorganic phosphate product, with the subunit remaining ADP‐bound (Pollard *et al*, 2000). In Step 2, the ADP‐bound subunit dissociates from the filament. In Step 3, the depolymerized subunit reloads with ATP, recycling the subunit for another round of assembly (Plastino & Blanchoin, 2019). One question that remains unanswered is how a dynamic steady state can be set up and maintained with the limited amount of components available inside the cell cytoplasm.

*In vitro* experiments, based on a minimal set of purified actin‐binding proteins, have dramatically enhanced our understanding of actin network dynamics, with the molecular insights obtained from these approaches complementing *in vivo* experiments. Such experiments have revealed the basics of actin network assembly by the Arp2/3 complex (Mullins *et al*, 1998), actin‐based motility and force generation (Loisel *et al*, 1999; Bernheim‐Groswasser *et al*, 2002), selectivity in actin network contraction (Reymann *et al*, 2012) or disassembly (Gressin *et al*, 2015), actin network competition (Suarez *et al*, 2015; Antkowiak *et al*, 2019), as well as cytoskeletal network cross talk (López *et al*, 2014; Henty‐Ridilla *et al*, 2016; Colin *et al*, 2018; Alkemade *et al*, 2022). However, these systems rarely reach the dynamic steady state that occurs in living cells, with networks either growing then stalling or disassembling. Some systems have improved upon this situation by reaching a balance between assembly and disassembly. For example, dynamic actomyosin networks were reconstituted *in vitro* close to a lipid bilayer (Sonal *et al*, 2019) or in *Xenopus* egg extracts encapsulated in oil droplets (Pinot *et al*, 2012; Tan *et al*, 2018; Malik‐Garbi *et al*, 2019). Those studies demonstrated that robust and tunable actin flows are regulated by two main components: the actin turnover rate and the network geometry. In parallel, reconstitution of dynamic networks with a balance between assembly and disassembly mediated by actin polymerization and disassembly by ADF/cofilin (Michelot *et al*, 2007; Akin & Mullins, 2008; Reymann *et al*, 2011; Manhart *et al*, 2019; Bleicher *et al*, 2020; Pollard *et al*, 2020) has allowed to better understand the role of the different molecular actors in the establishment of a dynamic steady state. However, those experiments were performed either with cell extracts where precise protein content is unknown or with purified proteins in unlimited volumes (therefore masking some crucial steps existing when a limited amount of component is available).

Indeed, one major challenge in investigating actin turnover and its impact on dynamic steady state in reconstituted systems is related to the number of available active molecular constituents over time. *In vitro* experiments use biologically relevant concentrations of the components, but they are typically performed in relatively large volumes. Therefore, in a large environment, the reservoir of components necessary for actin turnover will be unlimited. As a result, depletion of some components, a critical effect in small volumes like that of a cell, cannot be controlled or even accomplished, shifting thus the chemical kinetics to a nonbiological regime in which the lifetime of a self‐sustaining system cannot be studied. A growing number of studies have used cell‐sized compartment such as water in oil droplets, GUVs (giant unilamellar vesicles) or microchambers in order to mimic the cell volume and to evaluate the effect of biochemical or mechanical parameters on the cytoskeleton self‐organization (Soares e Silva *et al*, 2011; Alvarado *et al*, 2014; Miyazaki *et al*, 2015; Jia & Schwille, 2019; Bashirzadeh *et al*, 2021; Hsu *et al*, 2022). However, how cytoskeletal dynamics can be set up and maintained in these protein‐pool‐limited environments has been little studied. More precisely, the balance between assembly, disassembly, and recycling fluxes, coupled with energy supply to maintain dynamic actin structures, remains poorly understood.

Here, we ask how actin network assembly can be achieved over time in a cell‐sized compartment where subunit recycling is required to maintain its limited pool. To address this issue, we developed a novel experimental system that combines actin bead motility and microfabricated microwells. In our cell‐sized compartment, we first investigated the minimal biochemical conditions for establishing a sustained actin turnover over time. We quantified how assembly, disassembly, and recycling individually or collectively support the maintenance of a dynamic and stable actin structure over long periods of time. Fast recycling by cyclase‐associated protein plays a critical role in maintaining the pool of polymerizable actin monomers. This step depends on ATP concentration. Furthermore, we find that even in excess of ATP, the system loses its effectiveness overtime because aging of actin monomers limits the lifetime of actin turnover.

## Results

### Microwells are closed environments where actin assembly is limited over time

To study actin network dynamic, we used the well‐described bead motility assay, in which 4.5 μm diameter polystyrene beads are coated with an Arp2/3 complex‐activating nucleation‐promoting factor (NPF, Snap‐Streptavidin‐WA‐His, in this study; Cameron *et al*, 1999, 2001; Bernheim‐Groswasser *et al*, 2002; Akin & Mullins, 2008; Reymann *et al*, 2011). When these beads are introduced to a medium containing the Arp2/3 complex, actin, profilin, and capping protein (assembly conditions), an actin comet assembles and beads exhibit directional motility that can be sustained for hours (Fig 1B and C; Movie EV1).

**Figure EV1:**
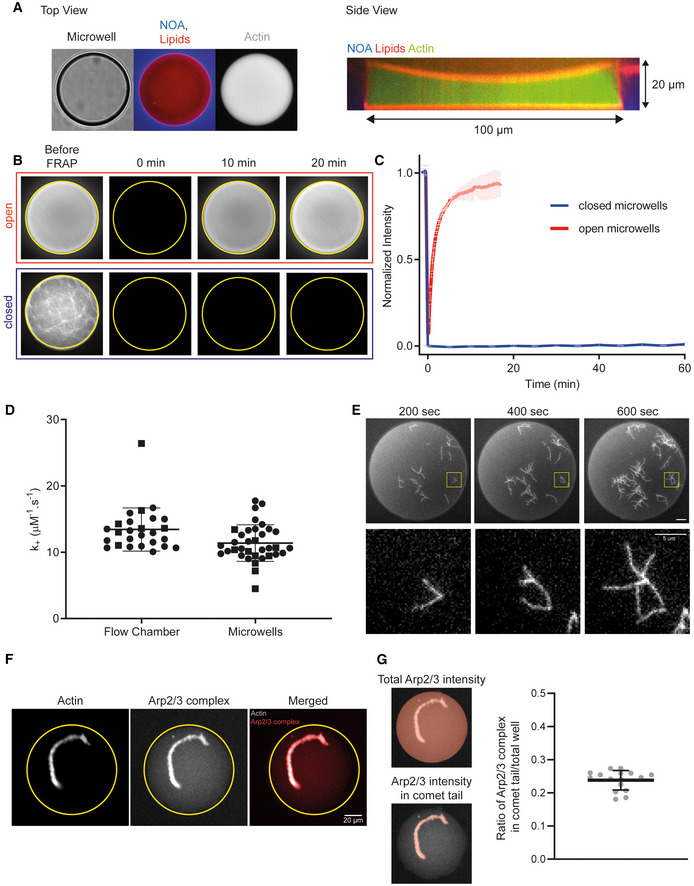
Microwells are closed environments that preserve key parameters for actin assembly Top and side views of microwells used in this study.Snapshots of FRAP experiment in open (Top) and closed (Bottom) microwells.Quantification of FRAP experiment in open and closed microwell. Closed wells: *N* = 3, *n* = 5 microwells. Open wells: *N* = 1, *n* = 2 microwells. Mean and standard deviation are represented.Quantification of the association rate constant of actin filament assembly at the barbed ends in flow chamber and in microwells. Biochemical conditions: [actin] = 0.8 μM. [profilin] = 2.4 μM. *N* = 2, *n* = 26 filaments for the flow chamber and *n* = 35 filaments for the microwells. Individual points are represented (1 symbol per independent dataset) with mean and standard deviation superimposed.Visualization of actin branched network formation in closed microwells (full well and zoom). Biochemical conditions: [actin] = 1 μM. [profilin] = 3 μM. [WA] = 50 nM. [Arp2/3 complex] = 25 nM.Snapshots of actin comet tail grown in assembly conditions with labeled Arp2/3 complex (see Materials and Methods).Quantification of the Arp2/3 complex incorporated in the comet tail over the total quantity of the Arp2/3 complex in the microwell. Biochemical conditions of the experiment: 4.5 μm polystyrene beads coated with 400 nM SNAP‐Strep‐WA‐His; 3 μM actin, 6 μM profilin, 90 nM Arp2/3 complex (labeled with Alexa647, see Materials and Methods), 15 nM capping protein. *N* = 1, *n* = 16 comet tails. Individual points are represented with mean and standard deviation superimposed.

To evaluate the effect of a limited amount of components on the lifetime of bead‐induced actin assembly, we used microwells (similar to the ones described in Yamamoto *et al*, 2022). They have a diameter of 100 μm and a height of 20 μm giving an approximate volume of 140 pl, five orders of magnitude smaller than the 20 μl volume of a classical flow chamber used in *in vitro* assays (Figs 1D and EV1A). The microwells were closed with oil and their internal surface passivated with lipids (Fig EV1A). The hermeticity of the microwells has been confirmed using FRAP experiment (Figs EV1B and EV1C). We validated that actin assembly is preserved inside microwells by measuring the association rate constant at the actin barbed end (k_+_) and comparing it to actin assembly in flow chamber (Fig EV1D). Finally, we also confirmed that the Arp2/3 complex machinery was functional (Fig EV1E). In all experiments, the time 0 of the reaction was when actin monomers were added to the mix before introducing it into the microwells and sealing them with oil.

We first compared single bead movement in a flow chamber (unlimited pool) and in the microwells (limited pool; Fig 1C and D; Movie EV1). In the flow chamber, the length of the comet increases linearly for ∼ 6 h while it reached a plateau after ∼ 3 h in microwells (Fig 1E). In consequence, the growth velocity of comet tails was constant over time in the flow chamber but decreases rapidly in microwells (Fig 1F). This suggests that in microwells the pool of actin monomers is limited and that after 3 h most of the monomers were consumed. Interestingly, when we doubled the initial concentration of actin monomers to 6 μM, the maximum comet length was 130 μm, whereas it was only 60 μm for an initial actin monomer concentration of 3 μM (Fig 1D and E). Furthermore, we found that the Arp2/3 complex and/or capping protein are still available in bulk during the experiment (Figs EV1F and EV1G and quantitative estimates in supplemental information). This demonstrates that in microwells, actin comet size is determined by the initial pool of monomers and that this pool is rapidly consumed over time.

Another argument for this limited number of components in microwells comes from the analysis of fluorescence profiles of the comet tails (Fig 1G). Indeed, actin fluorescence is constant over the entire tail of the comet in flow chamber (Fig 1G), but decreases in the region of the comet near the beads in microwells (Fig 1G). As new actin filaments are nucleated at the surface of the bead, this result confirms that the amount of polymerized actin in the wells decreases over time. Therefore, we sought conditions under which the actin monomer pool is maintained over time to improve the lifetime of actin assembly and bead motility.

### Sustained actin assembly in cell‐sized compartment requires disassembly and recycling

**Figure 2:**
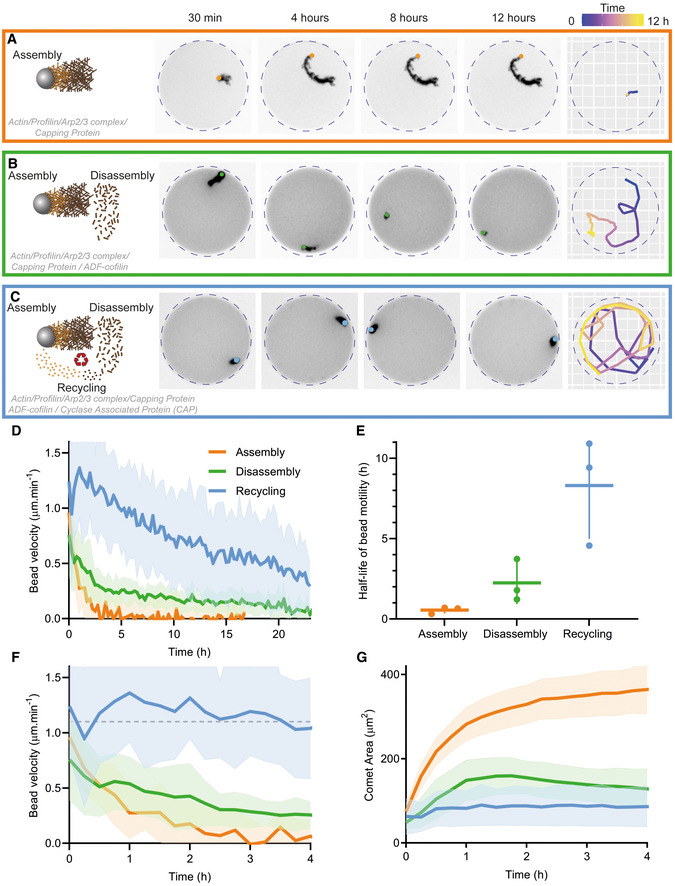
Contribution of assembly, disassembly, and recycling on actin turnover in cell‐sized compartment Left: Snapshots of actin comet tails assembled in Assembly conditions: 4.5 μm polystyrene beads coated with 400 nM SNAP‐Strep‐WA‐His; 3 μM actin, 6 μM profilin, 90 nM Arp2/3 complex, 15 nM capping protein. Right: Tracking of the comet shown in the snapshots. Time is encoded in color.Left: Snapshots of actin comet tails assembled in Disassembly conditions: same as assembly conditions with 200 nM ADF/cofilin added. Right: Tracking of the comet shown in the snapshots. Time is encoded in color.Left: Snapshots of actin comet tails assembled in Recycling conditions: Disassembly conditions with 400 nM cyclase‐associated protein (CAP) added. Right: Tracking of the comet shown in the snapshots. Time is encoded in color.Bead velocity as a function of time for one dataset per condition. Mean and standard deviation are represented.Estimation of half‐life of bead motility (from an exponential fit to the bead velocity curve) for each independent dataset in the different dynamic conditions (1 point per independent dataset).Same panel as panel D on a 4‐h timescale. The dashed line represents a constant velocity at 1.1 μm.min^−1^.Comet area as a function of time for one dataset per condition. Data information: Mean and standard deviation are represented. Assembly: *n* = 20 comet tails. Disassembly: *n* = 13 comet tails. Recycling: *n* = 16 comet tails.

**Figure EV2:**
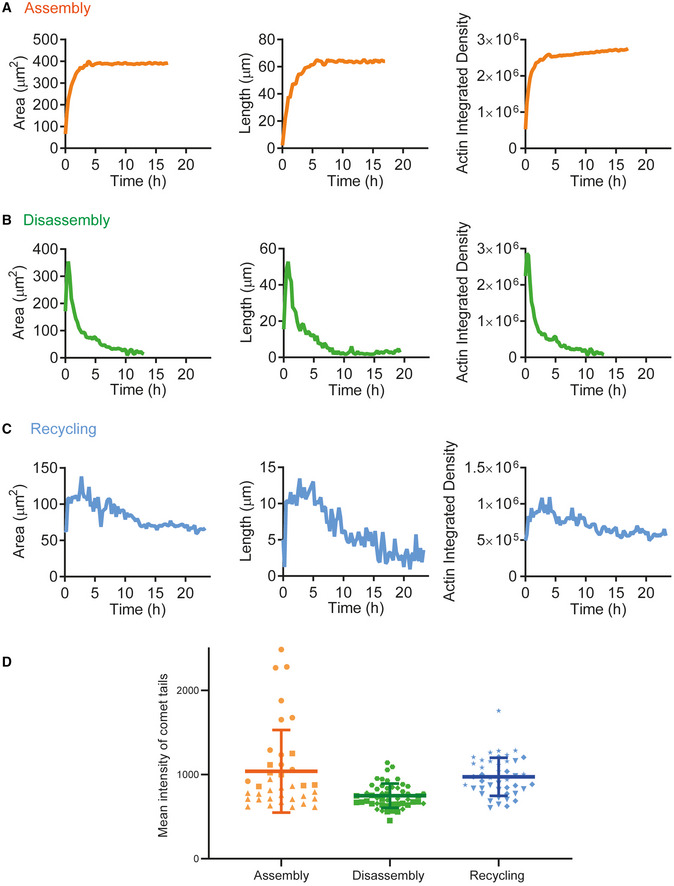
Quantitative analysis of actin within the comets in assembly, disassembly, and recycling conditions Estimation of length, area, and actin integrated density of the comet shown in Fig 2 for Assembly conditions.Estimation of length, area, and actin integrated density of the comet shown in Fig 2 for Disassembly conditions.Estimation of length, area, and actin integrated density of the comet shown in Fig 2 for Recycling conditions.Mean intensity (actin density) of comet tails for the different conditions reconstituted. Assembly: *N* = 3, 38 comet tails, Disassembly: *N* = 3, 50 comet tails, Recycling: *N* = 3, 45 comet tails. Each independent replicate is represented by a different symbol; mean and standard deviation are superimposed on top of each condition.

We first added the protein ADF/cofilin to our initial mixture because it is known to be a major actor of actin dynamics in cells (Lappalainen & Drubin, 1997; Dawe *et al*, 2003; Vitriol *et al*, 2013) and in reconstituted systems (Loisel *et al*, 1999; Suarez *et al*, 2011; Wioland *et al*, 2017). Actin comet tail disassembly was induced by the addition of 200 nM of ADF/cofilin. The choice of the ADF/cofilin concentration was based on our previous work on branched actin dynamics (Manhart *et al*, 2019) and the concentration dependence of ADF/cofilin activity (Andrianantoandro & Pollard, 2006). In these conditions, actin comets do not just assemble for a limited time (Figs 2A and EV2A; Movie EV3, Assembly conditions) but assemble and disassemble (Figs 2B and EV2B; Movie EV4, Disassembly conditions). The disassembly step following the addition of ADF/cofilin allowed the bead to move for more than 12 h (right panel in Fig 2B and D; Movie EV2 middle column) instead of only ∼ 3 h in the absence of ADF/cofilin (right panel in Fig 2A and D; Movie EV2 first column). In consequence, the half‐life of bead motility was increased in disassembly conditions compared with assembly conditions (0.6 h in Assembly conditions vs. 2 h in Disassembly conditions, Fig 2E). However, even in the presence of ADF/cofilin, the bead velocity decreased rapidly within the first 5 h of the experiment (Fig 2F and G). Since the pool of monomers is limited in our conditions, one possibility for the nonstability of the system is its inefficiency to recycle actin subunits or fragments generated by ADF/cofilin‐induced disassembly to recharge the pool of actin monomers bound to ATP.

In order to improve the recycling steps of the actin turnover, we added cyclase‐associated protein (CAP), which is well known to be important for actin dynamics in cells (Rust *et al*, 2020; Iwanski *et al*, 2021; Schneider *et al*, 2021). In addition, CAP is known to have a synergic effect with ADF/cofilin during actin disassembly, as demonstrated in cells (Bertling *et al*, 2004) and in *in vitro* (Normoyle & Brieher, 2012; Chaudhry *et al*, 2013; Kotila *et al*, 2019; Shekhar *et al*, 2019). CAP also catalyzes nucleotide exchange more efficiently than the profilin (Moriyama & Yahara, 2002; Chaudhry *et al*, 2010), therefore potentially playing a key role during actin monomer recycling. Addition of 400 nM of CAP, 2 excess molar ratio over ADF/cofilin (Chaudhry *et al*, 2014; Kotila *et al*, 2019; Figs 2C and EV2C; Movie EV5, Recycling conditions) further enhanced the half‐life of bead motility (8 h in Recycling conditions vs. 2 h in Disassembly conditions, Fig 2D and E; Movie EV2 right column). Importantly, in most cases, bead velocity and comet surface area stayed nearly constant for 4 h (Fig 2F and G), as expected for a dynamic steady state. These results show that we have successfully reconstituted a dynamic steady state in a compartment with a limited amount of components for an average lifetime of 4 h. However, this lifetime can be limited by additional factors that we will address in the following parts of this study.

### The actin monomer pool is recycled several times to maintain actin assembly in cell‐sized compartment

**Figure 3:**
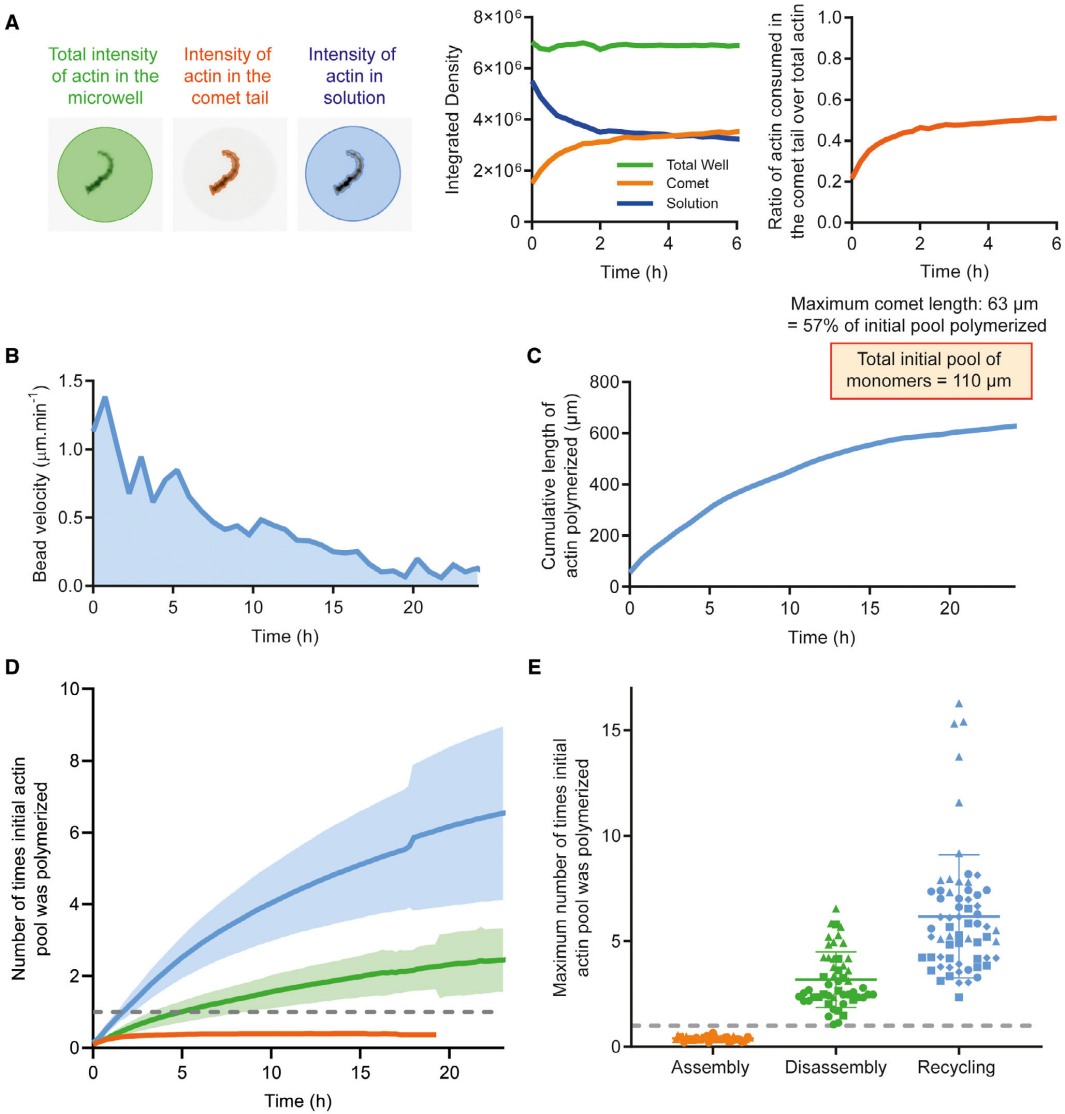
Actin monomers are recycled several times during sustained actin turnover in cell‐sized compartment Left: Estimation of the total actin amount in microwell and in the comet tail. Middle: Actin integrated density as a function of time for comet tail, solution, and total well. Right: Ratio of actin consumed in comet tail versus total actin as a function of time.Example of bead velocity as a function of time in recycling conditions. The integral of this curve (or area under curve) represents the length of actin polymerized in the comets during the time of the experiment.Example of cumulative length of actin polymerized as a function of time.Mean number of times initial actin amount was polymerized as a function of time (mean and standard deviation are shown). Assembly: *N* = 3, *n* = 38 comet tails. Disassembly: *N* = 3, *n* = 52 comet tails. Recycling: *N* = 4, *n* = 65 comet tails. The gray dashed line represents 1 cycle, which is equivalent to 3 μM, the initial concentration of actin introduced in the microwell.Maximum number of times the initial pool of actin monomers was polymerized in the microwell for the different conditions for actin‐based motility. The gray dashed line represents 1 cycle, which is equivalent to 3 μM, the initial concentration of actin introduced in the microwell. Individual points (1 symbol per independent dataset) for each comet are represented with mean and standard deviation superimposed. Assembly: *N* = 3, *n* = 38 comet tails. Disassembly: *N* = 3, *n* = 52 comet tails. Recycling: *N* = 4, *n* = 65 comet tails.

We sought to determine the amount of actin assembled in the comet as a function of time to assess the number of times the assembly/disassembly/recycling cycle was performed during sustained bead motility. First, in assembly conditions, we determined the amount of actin in the comet tails over time, by measuring the total fluorescence intensity of actin in the microwells and in the comet tail (Fig 3A). Given the critical concentration of ATP‐actin (0.1 μM), full polymerization should theoretically result in ∼ 96% actin in comet tails. We estimate that in our condition 57% of the actin initially introduced in the microwell assembles into the comet tail (the rest being likely polymerized in the bulk; Fig 3A). Quantitative estimates considering actin concentration and comet length confirm this value (see Materials and Methods).

**Figure EV3:**
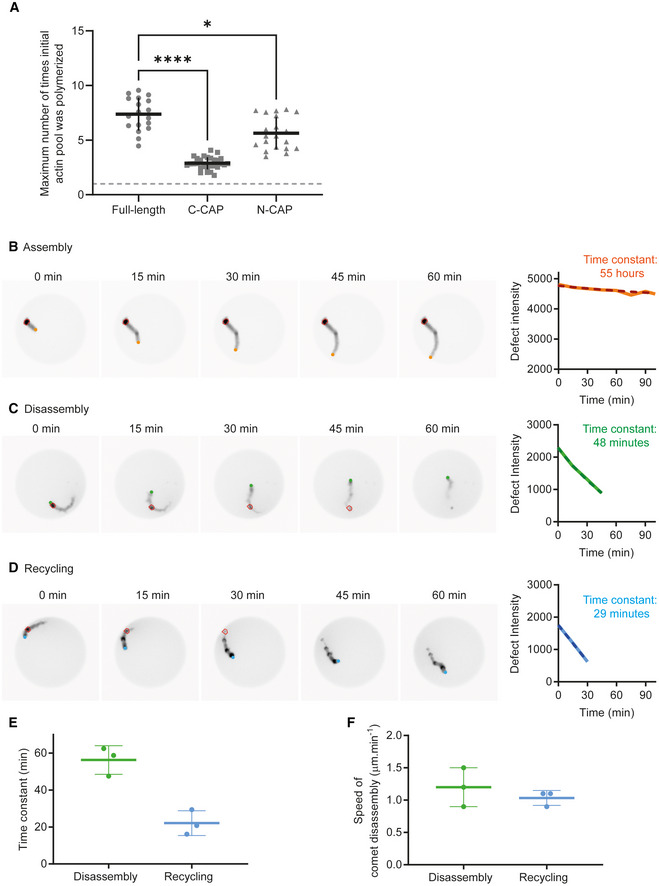
Quantitative estimates of the experimental system under assembly, disassembly, and recycling conditions AMaximum number of times the initial pool of actin monomers was polymerized in the microwell for the different CAP constructs. Full‐length: *N* = 2, *n* = 18 comet tails. C‐CAP: *N* = 2, *n* = 28 comet tails. N‐CAP: *N* = 2, *n* = 20 comet tails. Individual points are represented with mean and standard deviation superimposed. One‐way ANOVA statistics: Full‐length/C‐CAP: ****P‐value < 0.0001; Full‐length/*N*‐CAP: *P‐value < 0.05. The gray dashed line represents 1 cycle which is equivalent to 3 μM, the initial concentration of actin introduced in the microwell.B–Ddetermination of the rate of disassembly of the actin comets in assembly, disassembly, and recycling conditions. (B) Left: Snapshots of an actin comet tail assembled in Assembly conditions in a microwell. The bead is in orange and the tracked defect in red. Right: Defect fluorescence intensity as a function of time (solid line) and exponential fit (dashed line). Time constant is estimated from the exponential fit. (C) Left: Snapshots of an actin comet tail assembled in Disassembly conditions in a microwell. The bead is in green and the tracked defect in red. Right: Defect fluorescence intensity as a function of time (solid line) and exponential fit (dashed line). Time constant is estimated from the exponential fit. (D) Left: Snapshots of an actin comet tail assembled in Recycling conditions in a microwell. The bead is in blue and the tracked defect in red. Right: Defect fluorescence intensity as a function of time (solid line) and exponential fit (dashed line). Time constant is estimated from the exponential fit.EComet disassembly time in disassembly (*N* = 2, *n* = 3 comets) and recycling (*N* = 2, *n* = 3 comets) conditions. Individual points for each comet are represented with mean and standard deviation superimposed.FSpeed of comet disassembly in disassembly (*N* = 2, *n* = 3 comets) and recycling conditions (*N* = 2, *n* = 3 comets). Individual points for each comet are represented with mean and standard deviation superimposed.

In disassembly and recycling conditions, we computed the integrals of the bead velocity curves as a function of time (Fig 3B) in order to estimate the quantity of actin polymerized in the comet tail over time. Indeed, by computing the integral of the velocity curve, we obtained the cumulative length of actin polymerized as a function of time (Fig 3C). In order to convert the actin length into a quantity of actin polymerized (in μM), we used the fact that under assembly conditions, 57% of the actin initially introduced in the microwell assembles into the comet tail. As the mean actin comet length is 63 μm, the total initial pool of actin monomers represents _∼_ 110 μm of comet tail (we were able to use this assumption since the mean intensity of actin in comet tails does not vary significantly between the different reconstituted conditions, Fig EV2D). We used this factor to convert the length of actin polymerized in a quantity of actin polymerized. From those measurements, we were able to estimate the consumption of actin monomers by the system. In recycling conditions, the whole system consumed initially about 2 μM of actin monomer per hour whereas it was slower in assembly and disassembly conditions (Fig 3D). In disassembly conditions, the initial pool of actin monomers was assembled in the comet on average three times (Fig 3E), demonstrating that the presence of ADF/cofilin and profilin enables recycling of ADP‐actin subunits after disassembly as suggested before (Blanchoin & Pollard, 1998). In recycling conditions, the addition of CAP further improved the ability of the system to reuse the actin monomer pool in multiple cycles of actin turnover. Indeed, in the presence of CAP, the initial pool of actin monomers was assembled on average six times (and up to 17 times, Fig 3E). Because CAP can both increase the rate of actin filament disassembly and nucleotide exchange on subunits, we used N‐CAP and C‐CAP known to, respectively, accelerate filament depolymerization and recharge ADP‐actin monomers with ATP (Kotila *et al*, 2018, 2019). We saw that both activities were required for an efficient recycling (Fig EV3A).

The disassembly rate of the actin network was measured experimentally by tracking the fluorescence of actin defects in the comets and examining their fluorescence decay over time (Fig EV3B–D). Interestingly, under disassembly conditions, the mean disassembly time of the comet was 55 min whereas it was estimated to be 22 min on average under recycling conditions (Fig EV3E). When the disassembly time was “normalized” to the comet length, we were able to estimate a rate of network disassembly, which in both disassembly and recycling conditions was approximately 1.2 μm/min matching the rate of actin assembly (Fig EV3F).

Although our system approaches on average a dynamic steady state for 4 h under recycling conditions, the bead velocity decreases over time, and bead motility eventually stops (Fig 2D). This result suggests the presence of a limiting factor affecting the lifetime of our system. Since actin assembly consumes one ATP each time an actin monomer adds to the comet tail, we tested whether ATP depletion explains the decreasing velocity.

### ATP is necessary to maintain the dynamic steady state, but is not the limiting factor

**Figure 4:**
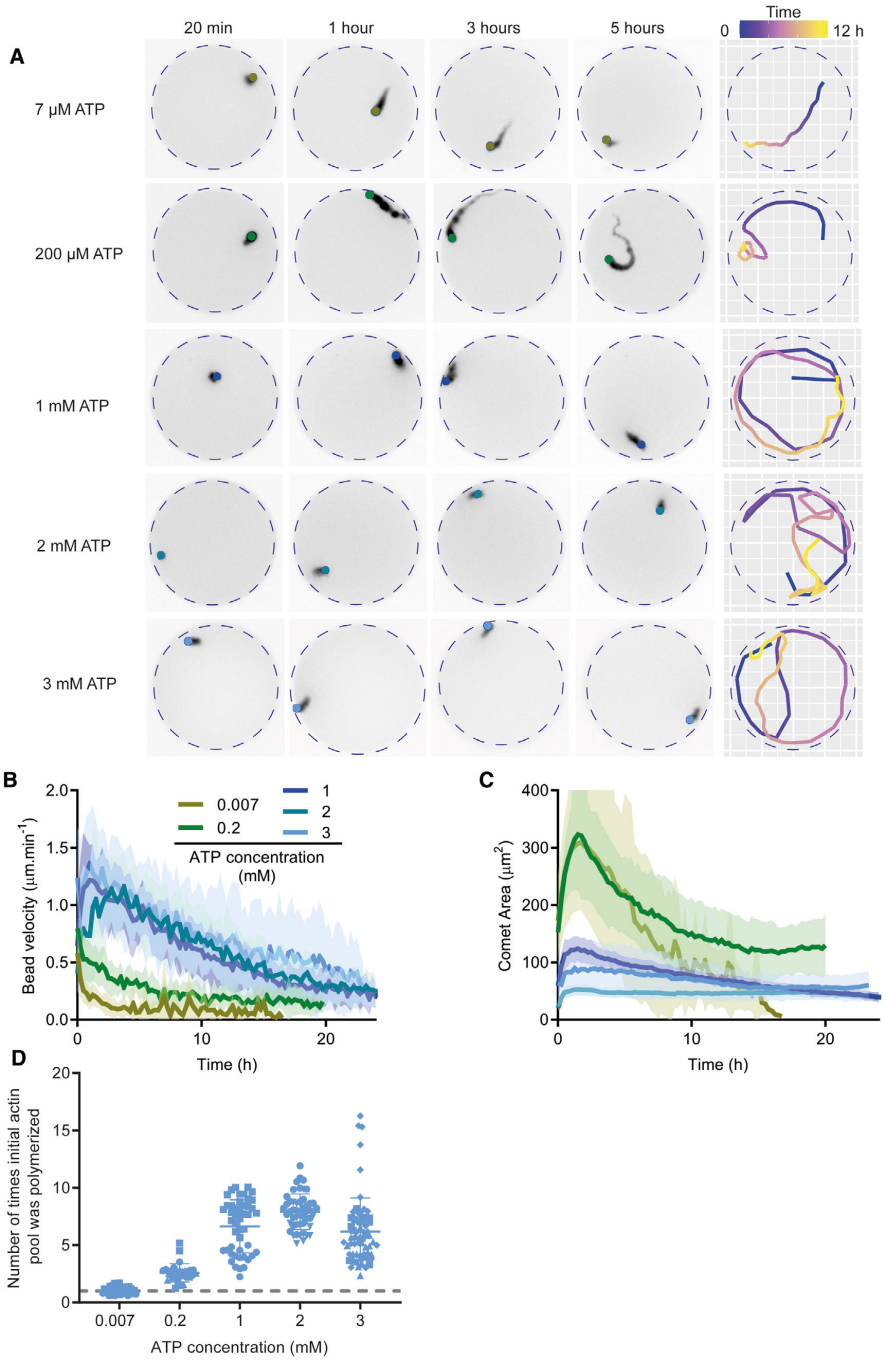
ATP is necessary for sustained actin turnover Left: Snapshots of actin comet tails in recycling conditions with the indicated ATP concentrations. Right: Tracking of the comet shown in the snapshots. Time is encoded in color.Bead velocity as a function of time for one dataset per condition. Mean and standard deviation are represented. [ATP] = 0.007 mM: *n* = 12 comet tails. [ATP] = 0.2 mM: *n* = 16 comet tails. [ATP] = 1 mM: *n* = 30 comet tails. [ATP] = 2 mM: *n* = 31 comet tails. [ATP] = 3 mM: *n* = 16 comet tails.Comet area as a function of time for one dataset per condition. Mean and standard deviation are represented.Number of times initial actin quantity was polymerized in the microwell for various concentrations of ATP. The gray dashed line represents 1 cycle which is equivalent to 3 μM, the initial concentration of actin introduced in the microwell. [ATP] = 0.007 mM: *N* = 2, *n* = 35 comet tails. [ATP] = 0.2 mM: *N* = 3, *n* = 30 comet tails. [ATP] = 1 mM: *N* = 2, *n* = 47 comet tails. [ATP] = 2 mM: *N* = 1, *n* = 31 comet tails. [ATP] = 3 mM: *N* = 4, *n* = 65 comet tails. Biochemical conditions: 4.5 μm polystyrene beads coated with 400 nM SNAP‐Strep‐WA‐His; 3 μM actin, 6 μM profilin, 90 nM Arp2/3, 15 nM capping protein, 200 nM ADF/cofilin, 400 nM cyclase‐associated protein (CAP) and ATP concentration as indicated.

**Figure EV4:**
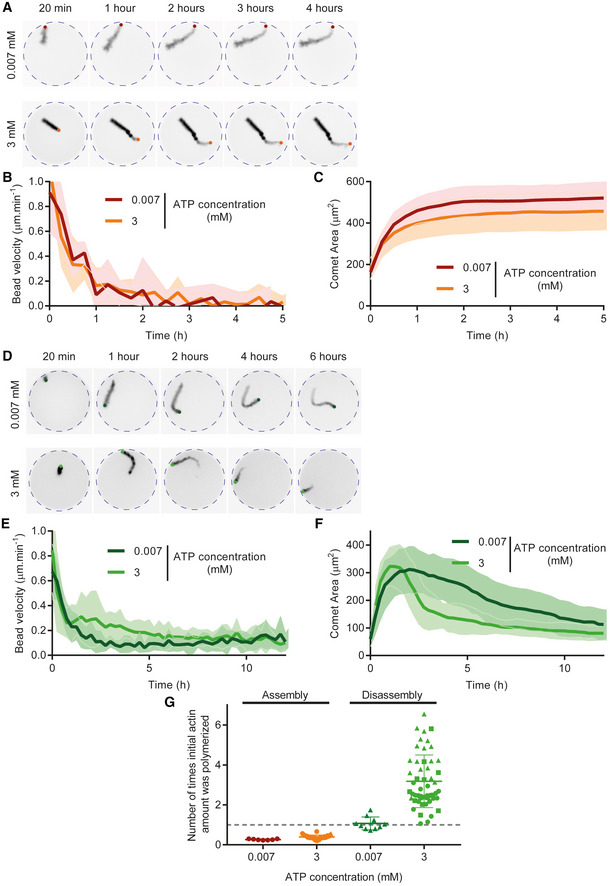
Effect of ATP concentration on actin‐based motility in assembly and disassembly conditions Snapshots of actin comet tails in assembly conditions with low ([ATP] = 0.007 mM) or high ([ATP] = 3 mM) ATP concentrations.Quantification of bead velocity for one dataset per condition in assembly conditions for low ([ATP] = 0.007 μM) or high ([ATP] = 3 mM) ATP concentrations. [ATP] = 0.007 μM: 7 comet tails. [ATP] = 3 mM: 11 comet tails.Quantification of comet area for one dataset per condition in Assembly conditions.Snapshots of actin comet tails in disassembly conditions with low ([ATP] = 0.007 mM) or high ([ATP] = 3 mM) ATP concentrations.Quantification of bead velocity for one dataset per condition in disassembly conditions for low ([ATP] = 0.007 μM) or high ([ATP] = 3 mM) ATP concentrations. [ATP] = 0.007 mM: 10 comet tails. [ATP] = 3 mM: 16 comet tails.Quantification of comet area for one dataset per condition.Number of times initial actin quantity was polymerized in the microwell for various concentrations of ATP in assembly or disassembly conditions. The gray dashed line represents 1 cycle which is equivalent to 3 μM, the initial concentration of actin introduced in the microwell. Assembly, [ATP] = 0.007 mM: *N* = 1, *n* = 7 comet tails. Assembly, [ATP] = 3 mM: *N* = 3, *n* = 38 comet tails. Disassembly, [ATP] = 0.007 mM: *N* = 1, *n* = 10 comet tails. Disassembly, [ATP] = 3 mM: *N* = 3, *n* = 52 comet tails. Biochemical conditions: 4 μm polystyrene beads coated with 400 nM SNAP‐Strep‐WA‐His; 3 μM actin, 6 μM profilin, 90 nM Arp2/3, 15 nM capping protein (Assembly), 200 nM ADF/cofilin (Disassembly). Individual points for each comet (1 symbol per independent dataset) are represented with mean and standard deviation superimposed.

We varied ATP concentration from 7 μM (coming only from the actin introduced in the reaction mix) to 3 mM (concentration used in the previous parts of the study) under assembly, disassembly, and recycling conditions (Figs 4 and EV4; Movie EV6). Interestingly, under assembly conditions, the initial concentration of ATP has minimal influence on the kinetics of comet growth (Fig EV4A) and the comet area and bead velocity were similar for both ATP concentrations (Fig EV4B and C). By contrast, ATP concentration has an effect in the presence of ADF/cofilin after the initial 2 h. At 3 mM ATP, comets started to disassemble and the length of the comets decreased while at low ATP concentration, this disassembly was less efficient (Fig EV4D–F). Of particular interest, the number of polymerization cycles of the initial monomer pool was independent of ATP concentration under assembly conditions (Fig EV4G) but increased significantly with higher ATP concentration under disassembly conditions (Fig EV4G).

We then varied the ATP concentrations under recycling conditions (Fig 4A; Movie EV6), adding several intermediate concentrations (200 μM, 1 and 2 mM) to the 7 μM and 3 mM conditions used previously. At low ATP concentrations (7 and 200 μM), comet dynamics resemble those of disassembly conditions, with an initial increase in comet area followed by a progressive decrease (Fig 4C), a rapid decrease in bead velocity over time (Fig 4C), and almost complete comet disassembly after 8 h (Fig 4A and C; Movie EV6). At high ATP concentrations (1, 2, and 3 mM), there is a dramatic increase in both bead velocity and comet area, although both decrease progressively over 20 h (Fig 4A–C). Estimation of the number of times the initial actin monomer pool was consumed for the different ATP concentrations revealed that 1 mM ATP is already a saturating ATP concentration (Fig 4D).

These results demonstrate that recycling generates faster and long‐lived systems that are more sensitive to energy input. Since energy was not the limiting factor to maintain the steady state in our experimental conditions, we hypothesized that another component of our system may have degraded during the experiment. To test this hypothesis, we decided to evaluate the aging of the different components of the system in our experiment.

### Actin aging limits the lifetime of actin assembly

**Figure EV5:**
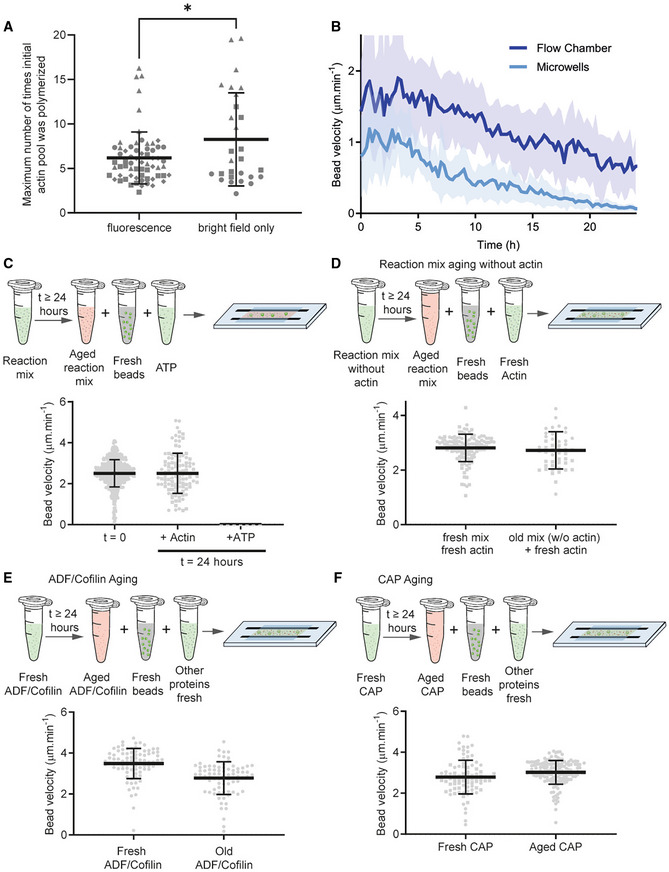
Determination of the aging factor in our biochemical assay AComparison of the number of times the initial actin pool was polymerized when the microwells were imaged by fluorescence or bright‐field imaging only. Fluorescence imaging: *N* = 4, *n* = 65 comets. Bright‐field only imaging: *N* = 3, *n* = 28 comets. Each independent replicate is represented by a different symbol; mean and standard deviation are superimposed on top of each condition. Unpaired *t*‐test statistics: fluorescence/bright‐field only: *P‐value = 0.016.BComparison of bead velocity in flow chamber and in microwells (one dataset in each condition, *n* = 37 comet tails in flow chamber; *n* = 13 comet tails in microwells).CEffect of ATP addition in an aged reaction mix. *t* = 0: *N* = 4, *n* = 494 comets. *t* = 24 h + actin: *N* = 2, *n* = 108 comets. *t* = 24 h + ATP: *N* = 1, *n* = 7 comets. Individual points for each comet are represented with mean and standard deviation superimposed.DTest of reaction mix aging without actin. Reaction mix was prepared without beads and without actin and left on the bench at room temperature. After 24 h, fresh beads and fresh actin monomers were added to the mix and bead velocity was estimated. Composition of the reaction mix: 6 μM profilin, 90 nM Arp2/3, 15 nM capping protein, 200 nM ADF/cofilin, 400 nM cyclase‐associated protein (CAP). Fresh mix, fresh actin: *N* = 2, *n* = 118 comets. Old mix (without actin) + fresh actin: *N* = 2, 49 comets. Individual points for each comet are represented with mean and standard deviation superimposed.E, FTest of ADF/Cofilin and CAP aging. Each protein was diluted in motility buffer and left on the bench at room temperature overnight. The morning after, the aged protein was added to the motility assay. Velocity of beads was estimated in the different conditions. Composition of the reaction mix: 3 μM actin, 6 μM profilin, 90 nM Arp2/3, 15 nM capping protein, 200 nM ADF/cofilin, 400 nM cyclase‐associated protein (CAP). Fresh ADF/cofilin: *N* = 1, *n* = 88 comet tails. Aged ADF/cofilin: *N* = 1, *n* = 81 comet tails. Fresh CAP: *N* = 2, *n* = 89 comets. Aged CAP: *N* = 1, *n* = 149 comets. Individual points for each comet are represented with mean and standard deviation superimposed.

We first confirmed that ROS (reactive oxygen species) production due to fluorescence imaging was not the main reason of the aging of the system. For that, we followed bead velocity using bright‐field imaging. We found that the number of times the initial actin monomer pool was polymerized was similar regardless of the imaging method used (Fig EV5A). The microwells are enclosed between a slide and a coverslip, implying that their content cannot be modified during the experiment. Therefore, addressing protein aging in microwells was technically challenging. We decided to compare under recycling conditions the slow decrease in bead velocity in the flow chamber (unlimited number of components) and in the microwells (limited number of components). We observed that, although variable, the rate of decrease in the velocity of the bead of 0.1 h^−1^ was similar in the two conditions (Fig EV5B). We therefore decided to investigate possible mechanism responsible for this decrease in the flow chamber.

**Figure 5:**
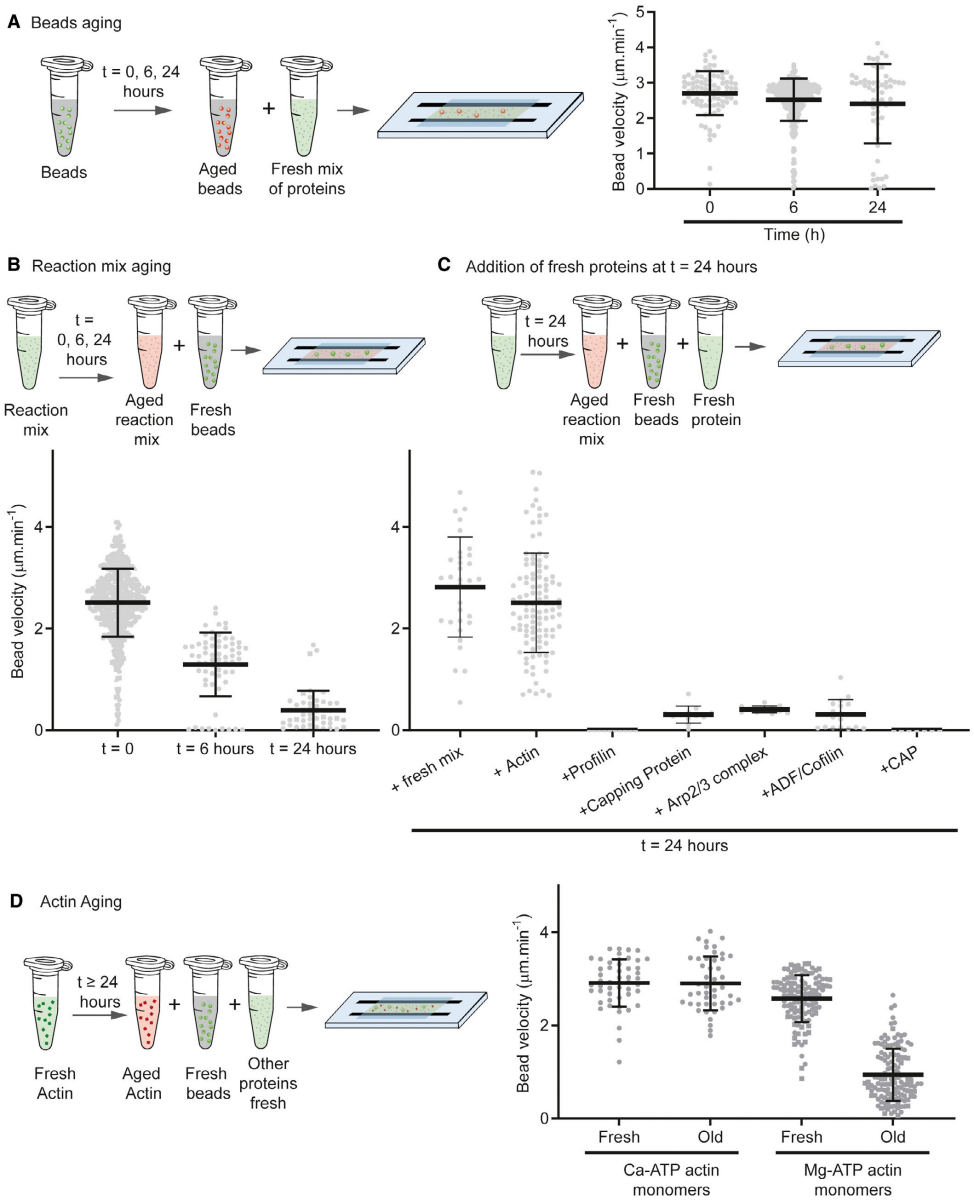
Aging of components during the actin dynamic steady state ATest of beads aging in motility buffer. Beads coated with 400 nM of SNAP‐Strep‐WA‐His were left on the bench and reintroduced into a new protein mixture at different elapsed times (0, 6 and 24 h). Bead velocity was estimated for those different times. Composition of the protein mix: 3 μM actin, 6 μM profilin, 90 nM Arp2/3, 15 nM capping protein, 200 nM ADF/cofilin, 400 nM cyclase‐associated protein (CAP). *N* = 1, *n* = 87 comets for *t* = 0, *n* = 292 comets for *t* = 6 h, *n* = 60 comets for *t* = 24 h. Individual points for each comet are represented with mean and standard deviation superimposed.B, CTest of reaction mix aging without beads. Reaction mix was prepared without beads and left on the bench at room temperature. Then, at different time intervals, fresh beads were added to the mix. After 24 h (right part of the graph), all (fresh) proteins in the assay were added one by one with fresh the beads. Velocity of fresh beads was estimated for those different times. Composition of the reaction mix: 3 μM actin, 6 μM profilin, 90 nM Arp2/3, 15 nM capping protein, 200 nM ADF/cofilin, 400 nM cyclase‐associated protein (CAP). *t* = 0: *N* = 4, *n* = 494 comets. *t* = 6 h: *N* = 1, *n* = 65 comets. *t* = 24 h: *N* = 2, *n* = 46 comets. *t* = 24 h + fresh mix: *N* = 1, *n* = 32 comets. *t* = 24 h + actin: *N* = 2, *n* = 108 comets. *t* = 24 h + profilin: *N* = 1, *n* = 10 comets. *t* = 24 h + capping protein: *N* = 1, *n* = 12 comets. *t* = 24 h + Arp2/3 complex: *N* = 1, *n* = 11 comets. *t* = 24 h + ADF/cofilin: *N* = 1, *n* = 17 comets. *t* = 24 h + CAP: *N* = 1, *n* = 7 comets. Individual points for each comet are represented with mean and standard deviation superimposed.DTest of actin monomer aging. Actin monomers were prepared in salt‐free motility buffer (Ca‐ATP monomers, see Materials and Methods) or in salt‐free motility buffer with EGTA and MgCl_2_ (Mg‐ATP monomers, see Materials and Methods) and left on the bench at room temperature overnight. The day after, aged actin monomers were added to a fresh mixture (without actin monomers) in presence of fresh beads. Velocity of beads was measured in the different conditions. Composition of the reaction mix: 3 μM actin, 6 μM profilin, 90 nM Arp2/3, 15 nM capping protein, 200 nM ADF/cofilin, 400 nM cyclase‐associated protein (CAP). Ca‐ATP‐actin monomers fresh: *N* = 1, *n* = 50 comets. Ca‐ATP‐actin monomers (old) *N* = 1, *n* = 46 comets. Mg‐ATP‐actin monomers (fresh): *N* = 2, *n* = 121 comets. Mg‐ATP‐actin monomers (old): *N* = 2, *n* = 134 comets. Individual points for each comet are represented with mean and standard deviation superimposed.

We first examined possible aging of the bead component, that is, whether the NPF proteins on the bead could become unstable or detach from the bead over time (Fig 5A). We prepared NPF‐coated beads, aged them at room temperature for 0, 6, or 24 h before adding them to a fresh motility mixture (Fig 5A; Movie EV7). The bead velocity was similar at these different time intervals (Fig 5A), suggesting that bead aging did not have a significant impact on the lifetime of our system.

We next tested the lifetime of the reaction mixture by allowing the mixture to age before adding fresh beads (Fig 5B). Bead velocity gradually decreased with the aging time of the reaction mixture (Fig 5B; Movie EV8), losing all motility after 24 h. These experiments demonstrate that protein aging in the reaction mixture is a limiting factor for the lifetime of the system. Moreover, the addition of a “fresh” mixture of proteins to the aged reaction allowed the beads to restore their initial velocity (Fig 5C; Movie EV9).

To identify the proteins that were aging in our system, we added separately each protein one by one to an aged motility mixture that had lost its activity (Fig 5C). In these experiments, only fresh actin monomers were able to restore the motility of the beads (Fig 5C). We verified that it was not the small amount of fresh ATP supplied by actin that restarted the system (Fig EV5C). To further confirm that aging of actin monomers was the cause of the loss of activity of the motility mixture, we aged the motility mixture in the absence of actin monomers and recovered the activity of a fresh mixture with fresh actin monomers (Fig EV5D). In addition, the fact that when actin monomers are added to an old mixture, comet growth is restored, demonstrates that the assembly machinery (Profilin, Arp2/3 complex, and capping protein) is still functional and that the aging of these components is not a limiting factor. We also tested the aging of the disassembly machinery (ADF/Cofilin and CAP) independently (Figs EV5E and EV5F). We found that neither protein ages significantly over the time course of the experiment.

Because Ca‐ATP‐actin monomers are known to be stable for days in storage G‐buffer after purification, these results were somewhat surprising. Therefore, we tested aging of Ca‐ATP‐actin monomers in salt‐free motility buffer to prevent polymerization. When added to the fresh motility mixture, the aged Ca‐ATP‐actin monomers were able to fully restore bead motility (Fig 5D; Movie EV10). Because an exchange of the divalent cation bound to the actin monomers from Ca^2+^ to Mg^2+^ occurs during actin polymerization, we tested the possibility that Mg‐ATP‐actin monomers age more rapidly than Ca‐ATP monomers. For that, we complemented the salt‐free motility buffer with EGTA and MgCl_2_ (see Materials and Methods and Blanchoin & Pollard, 1999). Interestingly, the aged Mg‐ATP monomers were unable to initiate bead motility, suggesting that aging of Mg‐ATP‐actin monomers was the main factor responsible for the loss of motility mixture activity over time.

We thus conclude that actin monomers are the limiting factor in the system and their aging causes motility to decrease over time.

## Discussion

Our results established the relative contribution of the three‐step cycle (assembly, disassembly, and recycling) on actin bead motility in the presence of a limited pool of building blocks. This allows us to propose a quantitative model of the control of actin turnover lifetime in a compartment with a limited amount of components for different scenarios.

**Figure 6:**
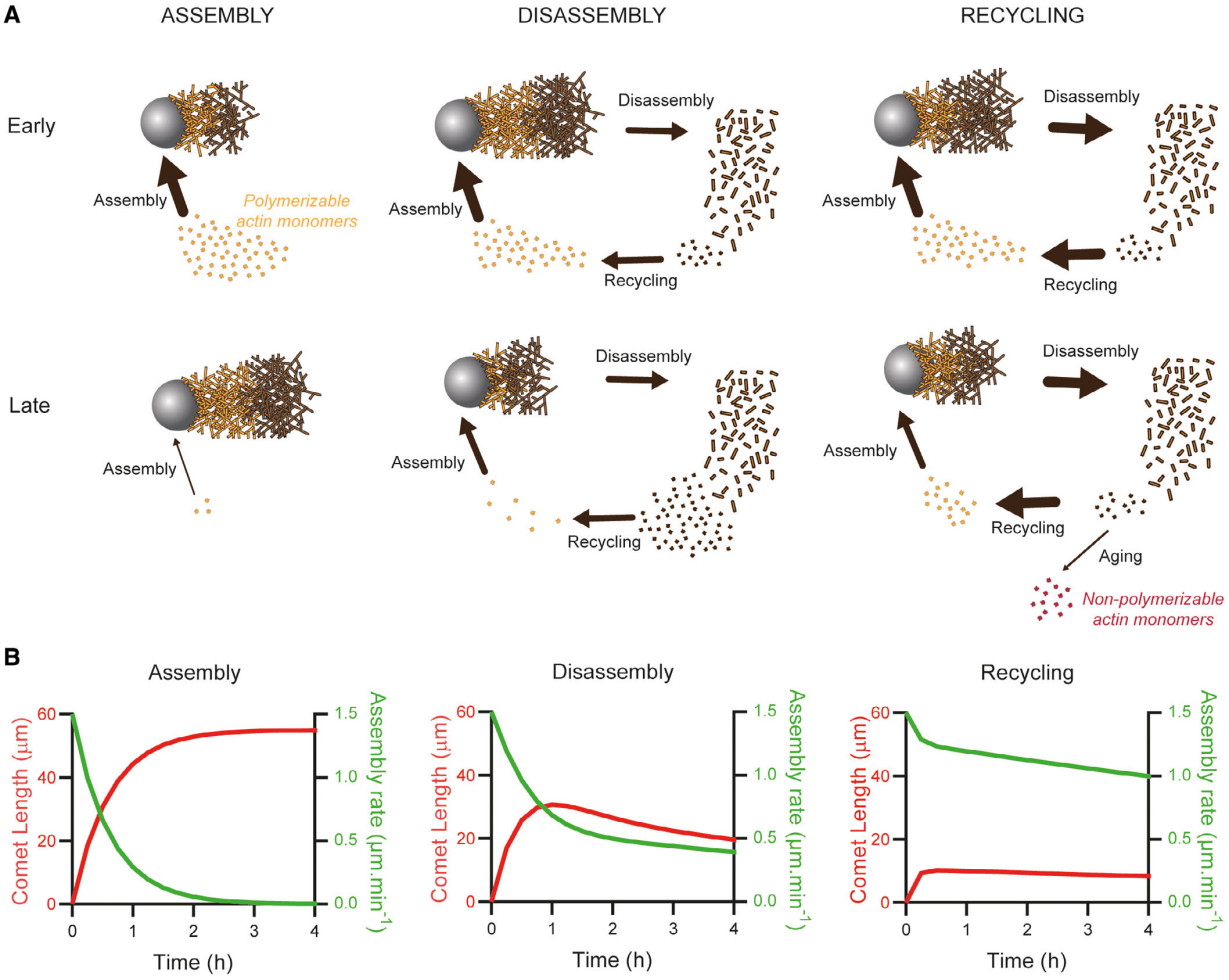
Quantitative model of the control of actin turnover lifetime in a cell‐sized compartment Summary scheme of the different conditions reconstituted in this study and the fluxes associated. The size of arrows scales with the value of the associated flux. Actin filament in orange represents the freshly assembled ATP or ADP‐Pi actin network, whereas actin in brown represents the ADP‐actin network.Model prediction of the assembly rate and comet length as a function of time in assembly, disassembly, and recycling conditions.

Under assembly conditions, at first, actin tail grows rapidly and consumes the actin monomer pool (Fig 6A, Early Assembly). Later, disassembly is too slow to compensate for the rapid monomer consumption and growth stops (Fig 6A, Assembly Late). This demonstrates that treadmilling limited by the rate of depolymerization at the filament ends cannot account for rapid actin turnover (Pollard & Borisy, 2003; Miyoshi & Watanabe, 2013; Blanchoin *et al*, 2014). A simple kinetic model (see Materials and Methods) considering the volume of the microwells, the concentration of the actin pool, and the rate of polymerization on the surface of the beads accurately predicts the variation of the comet growth speed as a function of time. The model also predicts accurately the comet length as a function of the initial pool of actin monomers (Fig 6B).

Under disassembly conditions, the initial rate of actin assembly is faster than disassembly (Fig 6A, Disassembly early). At early stage, the comet grows and the monomer pool decreases. As a result, the actin assembly rate decreases and at a given time corresponds to the disassembly rate. The comet then reaches a stable length over time. The actin network dynamic steady state is then limited by the recycling step necessary to maintain the pool of actin monomer (Fig 6A, Disassembly late). Interestingly, this system exhibits a feedback loop between assembly and disassembly via the monomer pool that allows the system to adapt to variations in operating rate over time (Fig 6B). This is in contrast to a system with an unlimited pool of monomers where a dynamic steady state of the actin network can be reached only in rare cases, when the initial assembly rate precisely matches the disassembly rate (Akin & Mullins, 2008; Manhart *et al*, 2019). Implementation in our model of the disassembly and recycling in presence of ADF/cofilin shows their impact on the length and growth speed of actin comets (Fig 6B).

Under recycling conditions, the rate of disassembly and recycling increases (see Materials and Methods for an estimate of these rates in disassembly and recycling conditions). The comet reaches a smaller stable length (Fig 6A, Recycling early). Fast recycling maintains the pool of actin monomer and increases the system lifetime (Fig 6A, Recycling late). The rate of actin network turnover in this condition (approximately 1 μm.min^−1^, Fig 6B Recycling) is very close to the rate measured *in vivo* for the lamellipodium (Wang, 1985; Theriot & Mitchison, 1991; Watanabe & Mitchison, 2002), demonstrating that we were able to reconstruct actin turnover at physiological rates in a cell‐sized compartment.

Fast recycling step was achieved by the protein CAP, which has emerged as a key regulator of actin dynamics *in vivo* (Rust *et al*, 2020). CAP works synergistically with ADF/cofilin to depolymerize actin filaments and subsequently catalyzes nucleotide exchange on the resulting monomers (Kotila *et al*, 2018, 2019; Shekhar *et al*, 2019). Our experiments provide evidence that, similar to cells, both acceleration of actin filament end depolymerization and nucleotide exchange on monomeric actin by CAP are important to reconstitute actin turnover at physiological rates.

Our work also emphasizes the need for a high recycling rate to maintain the pool of actin monomers necessary to participate in the assembly reaction. Most cells possess the complete recycling machinery that is composed of profilin and CAP (Paavilainen *et al*, 2004). However, in the absence of efficient monomer recycling, cells can use alternative strategies such as assembly by oligomer annealing, which has the advantage of being energetically more favorable (Okreglak & Drubin, 2010; Smith *et al*, 2013). Another possibility to overcome fast recycling rate is to maintain a large pool of nonpolymerizable monomers (most likely bound to thymosin‐ß4) that acts as a buffer allowing a temporary mismatch between assembly and recycling (Vitriol *et al*, 2015; Raz‐Ben Aroush *et al*, 2017).

How does this reconstituted *in vitro* system compare to the situation in a living cell? For example, in keratocytes and fibroblasts moving on a 2D surface, most actin polymerization takes place at the leading edge (Theriot & Mitchison, 1991). The leading edge is ∼ 30 μm wide and ∼ 0.2–0.4 μm high, so its surface area is ∼ 10 μm^2^. In comparison, a 4.5 μm bead has a surface on which polymerization takes place of the same order of magnitude. Thus, the actin assembly assay presented in this study is similar to that of the cell. However, in the cell, the total concentration of actin is ∼ 2 orders of magnitude higher than in the microwells (Pollard *et al*, 2000; Raz‐Ben Aroush *et al*, 2017; Funk *et al*, 2019), but the volume of the microwells is almost two orders of magnitude higher than that of the cell, so the total amount of actin in the microwells is comparable to that in the cell. (Note also that the length of the actin tail in the disassembly and recycling cases is on the order of a few microns to 10 microns, which is similar to the width of lamellipodial networks in living cells.) Thus, the main significant difference between the reconstituted system and a living cell is that the diffusion of actin monomers and actin‐binding proteins in a larger volume could become limited, as we have shown in previous studies (Boujemaa‐Paterski *et al*, 2017). However, the diffusion time is proportional to the square of the cell/chamber size. In this study, the characteristic size of the microwell is ∼ 100 μm and that of the motile living cell is ∼ 30 μm, so that the diffusion time in our case is at most ∼ 10 times larger than in the cell. However, considering that in the living cell diffusion is slowed by cytoplasmic crowding absent *in vitro* and that the flatness of the lamellae in living cells further slows the diffusive flow, our *in vitro* conditions quantitatively match those in living cells.

Actin turnover and the dynamic steady state of the network are intimately linked to energy consumption. Our results show that while assembly is independent of free ATP concentration, as long as an ATP is bound to an actin monomer (Blanchoin & Pollard, 1999), disassembly and recycling are very sensitive to ATP concentration. At low ATP concentration, recycling, which involves the exchange of the nucleotide bound to the actin monomer (Blanchoin & Pollard, 1998), is the limiting step in actin turnover. However, at high ATP concentration, recycling is almost instantaneous and disassembly becomes the limiting step. Interestingly, above 1 mM free ATP, the energy supply is not the factor defining the lifetime of our dynamic system. Since the concentration of ATP in a physiological context is well above 1 mM (Greiner & Glonek, 2021), our findings reinforced the notion that cells contained excessively high ATP concentration, compared with the concentration necessary to maintain actin organization at a dynamic steady state.

In addition to recycling, aging of actin monomers limits the lifetime of the system. Lifetime of *in vitro* systems has been overlooked in previous studies. Very few studies mentioned the lifetime of bead motility in unlimited volumes (Marchand *et al*, 1995; Akin & Mullins, 2008; Lacayo *et al*, 2012). We have shown that the aging of actin monomers is related to the nature of the divalent cation bound (Mg^2+^ or Ca^2+^). Mg‐ATP‐actin monomers lose their ability to polymerize over time, while Ca‐ATP‐actin monomers are very stable. The bound cation, Mg^2+^ or Ca^2+^ has been shown to modulate nucleotide binding and exchange on actin monomers, ATP hydrolysis activity, nucleation and polymerization (Estes *et al*, 1992; Blanchoin & Pollard, 1999; Cossio & Hocky, 2022; Oosterheert *et al*, 2022). The precise mechanism of aging dependent on divalent cations bound to actin monomers is not addressed here because it is beyond the scope of this study. Note, however, that in ATP‐actin monomer, Ca^2+^ is hepta‐coordinated whereas Mg^2+^ is hexa‐coordinated (Wang *et al*, 2010; Oosterheert *et al*, 2022). This keeps the nucleotide‐bound ATP, and the two halves of the actin monomer, in a slightly different conformation that facilitates polymerization of Mg‐ATP versus Ca‐ATP‐actin (Blanchoin & Pollard, 1999; Oosterheert *et al*, 2022). So, it is possible that age‐based denaturation of Mg‐ATP‐actin monomers occurs because the two halves of the actin monomers are not held together as tightly as they are for Ca‐ATP. It is also possible that Mg‐ATP‐actin becomes more sensitive to ROS. Cells may have overcome these issues with systems of chaperones (Dalle‐Donne *et al*, 2001; Wettstein *et al*, 2012; Grantham, 2020), by having an effective system of protein synthesis and degradation to maintain a pool of polymerizable actin monomers (Olson & Nordheim, 2010; Vedula *et al*, 2021) or by limiting oxidative stress (Rouyère *et al*, 2022).

To achieve structural stability and dynamics in cellular organization, the importance of balancing the supply and demand of building blocks in real time is crucial. Since cellular building blocks are often limited, recycling seems to be essential to keep this balancing mechanism under control and avoid a mismatch between supply and demand that would alter structural stability. Our system offers new opportunities to study the essential role of recycling in the balancing mechanism as a general principle for the dynamic steady state of intracellular organization. With this work, reconstituted systems were pushed to the limits that are key to cellular life: recycling and energy production to feed active assembly/disassembly as well as component self‐renewal to limit aging.

## Materials and Methods

### Protein purification

Actin was purified from rabbit skeletal muscle acetone powder (Spudich & Watt, 1971). Monomeric Ca‐ATP‐actin was purified by gel‐filtration chromatography on Sephacryl S‐300 at 4°C in G‐buffer (2 mM Tris–HCl, pH 8.0, 0.2 mM ATP, 0.1 mM CaCl_2_, 1 mM NaN_3_ and 0.5 mM dithiothreitol [DTT]). Actin was labeled on lysines with Alexa‐568 (Isambert *et al*, 1995). All experiments were carried out with 5% labeled actin. The Arp2/3 complex was purified from calf thymus according to (Egile *et al*, 1999) with the following modifications: the calf thymus was first mixed in an extraction buffer (20 mM Tris pH 7.5, 25 mM KCl, 1 mM MgCL2, 0.5 mM EDTA, 5% glycerol, 1 mM DTT, 0.2 mM ATP and proteases). Then, it was placed in a 50% ammonium sulfate solution in order to make the proteins precipitate. The pellet was resuspended in extraction buffer and dialyzed overnight. Arp2/3 complex was fluorescently labeled as described in Funk *et al* (2021).

Human profilin was expressed in BL21 DE3 pLys *Escherichia coli* cells and purified according to Almo *et al* (1994). Mouse capping protein was purified according to Palmgren *et al* (2001). Yeast cofilin was purified and fluorescently labeled according to Suarez *et al* (2011). The full‐length mouse cyclase‐associated protein 1 (CAP1) was purified in a similar fashion as described in Kotila *et al* (2019). To describe briefly, the CAP1 protein was expressed in *E. coli* as described earlier, or by using BL21 (DE3) *E. coli* cells (Sigma) and expression in LB medium at +16°C for 30 h. The bacteria were pelleted and resuspended to buffer A (50 mM Tris pH 7.5, 150 mM NaCl, 25 mM imidazole) and lysed by sonification in the presence of protease inhibitors (200 μg/ml PMSF, 1 μg/ml leupeptin, 1 μg/ml aprotinin, 1 μg/ml pepstatin A, and 150 μg/ml benzamidine hydrochloride, all from Sigma‐Aldrich). The supernatant, clarified by centrifugation, was then loaded to a 5 ml HisTrap Ni‐NTA column coupled to AKTA Pure protein purification system (GE Healthcare). The His‐SUMO‐tagged CAP1 protein was eluted from the nickel column with an imidazole gradient using buffer A and buffer B (buffer A + 250 mM imidazole), and the main peak fractions were collected and concentrated with Amicon Ultra‐15 30 kDa cutoff centrifugal filter device. The His‐SUMO‐tag was then cleaved from the CAP protein in the presence of SENP2 protease, after which the cleaved protein was subjected to gel filtration runs by using Superose 6 increase 10/300 GL gel filtration column equilibrated in 5 mM HEPES, 100 mM NaCl, 1 mM DTT, 1 μg/ml leupeptin, pH 7.4. Peak fractions from the same elution volume were combined, concentrated as above and snap‐frozen with liquid N_2_ for long‐term storage at −75°C. C‐CAP and N‐CAP were purified according to Kotila *et al* (2018) and Kotila *et al* (2019), respectively.

Snap‐Streptavidin‐WA‐His (pETplasmid) was expressed in Rosetta 2 (DE3) pLysS (Merck, 71403). Culture was grown in TB medium supplemented with 30 μg/ml kanamycin and 34 μg/ml chloramphenicol, then 0.5 mM isopropyl β‐D‐1‐ thiogalactopyranoside (IPTG) was added, and protein was expressed overnight at 16°C. Pelleted cells were resuspended in Lysis buffer (20 mM Tris pH8, 500 mM NaCl, 1 mM EDTA, 15 mM Imidazole, 0.1% TritonX100, 5% Glycerol, 1 mM DTT). Following sonication and centrifugation, the clarified extract was loaded on a Ni Sepharose high‐performance column (GE Healthcare Life Sciences, ref 17526802). Resin was washed with Wash buffer (20 mM Tris pH8, 500 mM NaCl, 1 mM EDTA, 30 mM Imidazole, 1 mM DTT). Protein was eluted with Elution buffer (20 mM Tris pH8, 500 mM NaCl, 1 mM EDTA, 300 mM Imidazole, 1 mM DTT). Purified protein was dialyzed overnight 4°C with storage buffer (20 mM Tris pH8, 150 mM NaCl, 1 mM EDTA, 1 mM DTT), concentrated with Amicon 3KD (Merck, ref UFC900324) to obtain concentration around 10 μM then centrifuged at 160,000 *g* for 30 min. Aliquots were flash‐frozen in liquid nitrogen and stored at −80°C.

### Polystyrene beads coating

Polystyrene beads coating with NPF was done following classical protocols (Reymann *et al*, 2011). A 4.5 μm polystyrene beads (Polybeads Carboxylate 4.5 microns; 2.6% solids‐latex) were centrifuged at 13,000 *g* for 2 min on a mini spin plus Eppendorf centrifuge (Rotor F45‐12‐11). The pellet was then resuspended in 50 μl of a 400 nM SNAP‐Strep‐WA‐His solution. Beads were incubated for 15 min at 15°C at 950 rpm in a thermoshaker. They were then centrifuged for 2 min at 3,800 *g*, resuspended in 200 μl of BSA 1%, and let on ice for 5 min. Beads were finally centrifuged again 2 min at 3,800 *g* and resuspended in 50 μl of BSA 0.1%.

### Microwells preparation

SU8 mold with pillars was prepared using standard protocols and silanized with Trichloro(1H,1H,2H,2H‐perfluoro‐octyl)silane for 1 h and heated for 1 h at 120°C. From the SU8 mold, a PDMS primary mold was prepared (Dow, SYLGARD 184 silicone elastomer kit) with a 1:10 w/w ratio of curing agent. PDMS was cured at 70°C for at least 2 h. PDMS primary mold was then silanized with Trichloro(1H,1H,2H,2H‐perfluoro‐octyl)silane for 1 h and heated for 2 h at 100°C. PDMS was then poured on top of the PDMS primary mold to prepare the PDMS stamps.

Coverslips were cleaned with the following protocol: They were first wiped with ethanol (96%) and then sonicated for 15 min in ethanol. After the first sonication, coverslips were rinsed 3 times with mqH_2_O. They were then sonicated for 30 min in Hellmanex 2% at 60°C. After this second sonication, coverslips were rinsed in several volumes of mqH_2_O and kept in water until use. Just before use, coverslips were dried with compressed air.

For the microwells preparation, PDMS stamps were cut in pieces and placed on the coverslips with the pillars facing the coverslip. A droplet of NOA 81 (Norland Products) was then placed on the side of the PDMS stamp, and NOA was allowed to go through the PDMS stamp by capillarity. When the NOA filled all the stamp, it was polymerized with UV light for 12 min (UV KUB2/KLOE; 100% power). After polymerization of the NOA, PDMS stamp was removed and the excess of NOA was cut. Then, an additional UV exposure of 2 min was done and the microwells were placed on a hot plate at 110°C for at least 3 h (or at 60°C overnight) to tightly bind the NOA to the glass.

### Lipids/SUV preparation

l‐α‐phosphatidylcholine (EggPC; Avanti, 840051C) and ATTO 647N labeled DOPE (ATTO‐TEC, AD 647N‐161 dehydrated) were used. Lipids were mixed in glass tubes as follows: 99.75% EggPC (10 mg/ml) and 0.25% DOPE‐ATTO647N (1 mg/ml). The mixture was dried with nitrogen gas. The dried lipids were incubated in a vacuum overnight. After that, the lipids were hydrated in the SUV buffer (10 mM Tris (pH 7.4), 150 mM NaCl, 2 mM CaCl_2_). The mixture was sonicated on ice for 10 min. The mixture was then centrifuged for 10 min at 20,238 *g* to remove large structures. The supernatants were collected and stored at 4°C.

### SilanePEG30k slides

SilanePEG (30 kDa, PSB‐2014, Creative PEG works) was prepared at a final concentration of 1 mg/ml in 96% ethanol and 0.1%(v/v) HCl. Slides were cleaned with the following protocol: they were sonicated for 30 min at 60°C in Hellmanex 2%. They were then rinsed with several volumes of mqH_2_O. Just before use, they were dried with compressed air. Slides were plasma cleaned for 5 min at 80% power and directly immersed in the silanePEG solution. They were kept in the silanePEG solution until use.

### Bead motility in microwells assay

A typical experiment of bead motility in microwells was performed as follows. The coverslip with microwells was activated with plasma for 2 min at 80% power. Just after the plasma, the flow chamber was mounted with the microwells coverslip, a slide passivated with SilanePEG 30 k and 180 μm height double‐side tape. Lipids were then inserted in the flow chamber and incubated for 10 min. Lipids were then rinsed with 600 μl of SUV buffer and 200 μl of HKEM buffer (50 mM KCl, 15 mM HEPES pH = 7.5, 5 mM MgCl_2_, 1 mM EGTA). The reaction mix with the different proteins was then prepared and flowed in the flow cell.

A typical reaction mix was prepared with beads coated with SNAP‐Strep‐WA‐His (activator of the Arp2/3 complex) and 3 μM of actin monomers, 6 μM profilin, 90 nM Arp2/3 complex, 15 nM capping protein in HKEM Buffer and was supplemented with 0.7% BSA, 0.2% methylcellulose, 2.7 mM ATP, 5 mM DTT, 0.2 mM DABCO (motility buffer). When needed, the polymerization mix was also supplement with yeast cofilin and/or cyclase‐associated protein (CAP). The microwells were then closed with mineral oil (Paragon scientific Viscosity Reference Standard RTM13). The whole flow cell was then closed with VALAP and imaged under the microscope.

### Bead motility in bulk environment

Bulk experiments were performed in a flow chamber made in the following way. A coverslip passivated with lipids or SilanePEG 30 k was mounted with a slide passivated with SilanePEG 30 k with double table of 70 μm height. The mix was injected in the flow chamber which was then sealed with VALAP.

### Aging of actin monomers

Ca‐ATP‐actin monomers were aged in a salt‐free motility buffer: G‐buffer with 0.25% methylcellulose, 2.7 mM ATP, 5 mM DTT, 0.2 mM DABCO. Mg‐ATP‐actin monomers were aged in a salt‐free motility buffer complemented with 25 μM MgCl_2_ and 1 mM EGTA.

### Imaging

Most of the experiments were done with an epifluorescence system (Ti2 Nikon inverted microscope equipped with a Hamamatsu Orca Flash 4.0 LT Plus Camera). The following objectives were used: Plan Fluor 10X DIC and S Plan Fluor ELWD 20X DIC. Time lapse were acquired with the NIS elements software (version 4.60).

Z‐stacks of microwells were performed with a confocal spinning disk system (EclipseTi‐E Nikon inverted microscope equipped with a CSUX1‐A1 Yokogawa confocal head), an Evolve EMCCD camera (Photometrics), Plan Fluor 60X objective. Z‐stacks were acquired with Metamorph software (Universal Imaging).

FRAP, k + estimation and visualization of branched actin network formation was done on a total internal reflection fluorescence (TIRF) microscopy instrument composed of a Nikon Eclipse Ti, an azimuthal iLas^2^ TIRF illuminator (Roper Scientific), a × 60 NA1.49 TIRF objective lens and an Evolve EMCCD camera (Photometrics). Time lapse and FRAP were done with Metamorph software (Universal Imaging).

### Image analysis

Images were analyzed with FiJi (Schindelin *et al*, 2012). Data were processed with R software and plotted with GraphPad Prism. Mean and standard deviation are represented for all the data. The dot plots show the individual values with the mean and standard deviation superimposed.

Actin comets were detected with the following procedure. First, threshold was adjusted manually and images were binarized. Actin comets were detected with the Analyze particles function. Comet length was obtained with the “skeletonize” and “analyze skeleton” functions. Comet growth velocity was obtained by calculating the length difference at each time point. Tracking of comets was then done with the TrackMate plugin using the thresholding detector (Ershov *et al*, 2022). Comet velocity was calculated from the (x, y) coordinates obtained with the trackmate tracking. Fluorescence profiles were manually drawn on the comet tail. Bead was detected from binarization and analyzed particles of the bright field movies.

#### Estimation of the ratio of actin consumed from bulk in the comet tail

To estimate the total quantity of actin in the microwell, we estimated the total fluorescence intensity. This value was constant during the time course of an experiment, showing that the actin in the microwell is constant during an experiment. The quantity of actin inside a comet tail was estimated after thresholding and binarization of the comet. Those two fluorescence intensities were corrected for the fluorescence background. Then, we estimated the ratio of actin consumed from the bulk in the comet by computing the comet fluorescence over the total fluorescence of the microwell.

Half‐life of bead motility was estimated by doing an exponential fit on the bead velocity curve for each independent dataset.

#### Estimation of k+

To estimate k+, we manually measured the length of actin filaments as a function of time.

### Quantitative estimates

#### Comparison of bead density in the flow chamber and in the microwells

In microwells, there is on average 1 bead per microwell, meaning 1 bead/140 pl. In flow chamber, we observe around 20 beads per field of view (10X field of view). The estimated volume of the field of view is 120,000 pl. Therefore, the bead density in the flow chamber is around 0.02 bead/140 pl. This means that the beads are 50 times more diluted in the flow chamber than in the microwell.

#### Estimates of the numbers of actin molecules

The microwell is a cylinder with radius *R* = 50 μm and height *H* = 20 μm, so the volume of the chamber is $W=\pi R^{2}H\approx 3\times {\left(50\;\mathrm{\mu m}\right)}^{2}\times 20\;\mathrm{\mu m}=1.5\times 10^{5}\;{\mathrm{\mu m}}^{3}$. There are about 600 molecules in one cubic micron of a solution with ∼ 1 μM concentration (to reflect that, we use parameter $\omega \approx 600/\left(\mathrm{\mu M}\cdot {\mathrm{\mu m}}^{3}\right)$), so the microwell contains $\omega \times W\times 3\;\mathrm{\mu M}\approx 3\times 10^{8}$ actin subunits. About 50% of actin subunits are assembled into the actin tail in the “assembly” case, so ∼ 1.5 × 10^8^ actin subunits are in the longest tail. Thus, the total length of all filaments in the tail is $1.5\times 10^{8}\times 2.7\;\mathrm{nm}\approx 4\times 10^{5}\;\mathrm{\mu m}$. Maximal actin tail length in the “assembly” case is *l* ∼ 60 μm (Fig EV2A), so *N*_*fil*_ ∼ 4 ×10^5^/60 ∼ 6,700 filaments are at every cross section of the tail. Considering that the beads are 4 microns in diameter, the cylindrical tail's cross‐section area is ∼*πR* ^2^ ∼ 12 μm^2^, the mesh size of the actin network (average distance between neighboring filaments), ζ, is on the order of $\zeta ∼\sqrt{\pi R^{2}/N_{\mathrm{fil}}}∼40\;\mathrm{nm}$, which is of the range widely reported in the literature (Kawska *et al*, 2012; Pujol *et al*, 2012).

#### Arp2/3 complex and capping protein

To validate that the Arp2/3 complex or capping protein is not depleted globally over the time of the experiment, we make the following estimates. The total length of all filaments in the tail is $1.5\times 10^{8}\times 2.7\;\mathrm{nm}\approx 4\times 10^{5}\;\mathrm{\mu m}$. The mean filament length observed in branched networks *in vitro and in vivo* is ∼ 300 nm (Vinzenz *et al*, 2012; Bieling *et al*, 2016). Therefore, there are about 1.3 × 10^6^ filaments in the comet tail.

Capping protein concentration is 15 nM, and this represents 1.5 × 10^6^ molecules of capping protein. Arp2/3 complex concentration is 90 nM, and this represents $10^{7}$ molecules of Arp2/3 complex.

If we consider one Arp2/3 complex and one capping protein per filament in the comet tail, we see from the number of molecules that there are enough proteins so that capping protein and Arp2/3 complex are not depleted over the time of the experiment.

This represents 13% of the total number of Arp2/3 complex in the comet tail. This number is on the same order of magnitude as that found experimentally.

In conclusion, the experimental observations and the numerical estimations confirm that Arp2/3 complex and capping protein are not globally depleted over the time course of the experiment in assembly conditions.

#### Estimates of the kinetics

##### Speed

Growth speed of the actin network is $V=k_{\mathrm{on}}\mathrm{\delta G}\Phi$, where $k_{\mathrm{on}}\approx 10/\mathrm{\mu M}\cdot s$ (Fig EV1D) is the polymerization rate, δ≈0.003μm is the half‐size of actin monomer, *G* is the G‐actin concentration, and Φ is the dimensionless factor that accounts for geometric (filaments are not exactly parallel to the tail's long axis), mechanical (slower growth against a mechanical load) and diffusion‐limited (depletion of the local G‐actin concentration at the bead‐tail interface by the “consumption” of monomers by growing barbed ends) factors. Normally, parameter Φ is in the range of 0.1–1. Considering that the observed initial growth rate of the actin tail is ∼ 1 μm/min ∼ 0.02 μm/s and that $k_{\mathrm{on}}\delta \bar{G}∼0.1\;\mathrm{\mu m}/s$ at total $\bar{G}=3\;\mathrm{\mu M}$, reasonable value of factor Φ∼0.2 explains the data.

##### Observed kinetics of the tail growth and fraction of actin polymerized in the “assembly” case

It is unlikely that the tail growth stops when critical actin concentration is reached: at the observed 50% of the assembled actin, the remaining actin concentration is too high to be critical. We therefore hypothesize that some of F‐actin is not part of the actin tail but rather short actin filaments or oligomers that either polymerize near the bead and do not connect to the tail's network, or spontaneously polymerize and then diffuse in the solute of the microwell, or both. Let *l* be the tail length, and $\tilde{l}$ be the total length of diffuse nontail filaments arranged into a “virtual tail” of the same geometry as the real one. The rate of filaments' assembly is $V=k_{\mathrm{on}}\mathrm{\delta G}\Phi$. Thus, without ADF/cofilin: $\frac{\mathrm{dl}}{\mathrm{dt}}=V=k_{\mathrm{on}}\mathrm{\delta G}\Phi$. Monomer concentration is $G=\bar{G}\left(1\;-\;\frac{l+\tilde{l}}{l_{\max}}\right)$ where $l_{\max}$ is the maximal length of the actin tail that would be achieved if *all* actin is assembled into one tail, and $\bar{G}$ is the initial monomer concentration. Therefore,

(1) $$\frac{\mathrm{dl}}{\mathrm{dt}}=k_{\mathrm{on}}\delta \bar{G}\Phi \left(1-\frac{l+\tilde{l}}{l_{\max}}\;\right)=V_{0}\left(1-\frac{l+\tilde{l}}{l_{\max}}\;\right)$$

where $V_{0}=k_{\mathrm{on}}\delta \bar{G}\Phi ∼1\frac{\mathrm{\mu m}}{\min}$, or $V_{0}=\tilde{k}l_{\max},\tilde{k}=k_{\mathrm{on}}\delta \Phi \frac{\bar{G}}{l_{\max}}∼0.01\frac{\mathrm{\mu m}}{\min}$.

Similar equation for the nontail F‐actin is:

(2) $$\frac{d\tilde{l}}{\mathrm{dt}}=\sigma V_{0}\left(1-\frac{l+\tilde{l}}{l_{\max}}\;\right)$$

where σ is the parameter that accounts for the fraction of the nontail assembly.

The solution of equation system (1–2) is: $l=\frac{l_{\max}}{1+\sigma }\left(1-\exp\left(-\left(1+\sigma \right)\frac{V_{0}t}{l_{\max}}\right)\right)$ and $\tilde{l}=\frac{\sigma l_{\max}}{1+\sigma }\left(1-\exp\left(-\left(1+\sigma \right)\frac{V_{0}t}{l_{\max}}\right)\right)$.

A few conclusions can be reached from these calculations:

- The model predicts that, in the “assembly” case, the tail length grows linearly at first and then exponentially saturates to the maximal length, as observed.
- In this case, the order of magnitude of the timescale on which the growth speed decreases, and the tail length stabilizes is the ratio of the max tail length to the initial velocity, $T∼l_{\max}/2V_{0}∼100\;\mathrm{\mu m}/2\;\mathrm{\mu m}/\min∼1\;h$, as observed.

##### General analysis of the dynamic steady state for assembling and disassembling actin tail in the case of no aging

In this analysis, we temporarily ignore the aging effect. As for the “unproductive” actin assembly, in the presence of ADF/cofilin, the short actin filaments that are not part of the tail are likely disassembled rapidly, and for simplicity, we omit the small fraction of diffuse short filaments from the analysis. We consider the following actin cycle: There is the actin tail of length *l* elongating with velocity *V* and disassembling into ADP‐G‐actin with rate γ. The net assembly flux is *V*, and the net disassembly flux is *γl*. The equation for the tail length is:

(3) $$\frac{\mathrm{dl}}{\mathrm{dt}}=V-\mathrm{\gamma l}$$

Note that area and length of the tail are proportional to each other, as the cross‐section area of the tail can be considered roughly constant. For convenience, in this analysis we use the tail length. ADP‐G‐actin concentration generated by the tail's F‐actin disassembly is $G_{D}$; the disassembly flux replenishing this concentration is ${\text{flux}}_{\text{disassembly}}=\mathrm{\gamma l}$. ADP‐G‐actin is recycled into ATP‐G‐actin, which concentration is $G_{T}$. Respective recycling flux is ${\text{flux}}_{\text{recycling}}=k_{\mathrm{DT}}\times \;G_{D}$. The dynamic equation for the ADP‐G‐actin concentration has the form:

(4) $$\frac{{\mathrm{dG}}_{D}}{\mathrm{dt}}=\mathrm{\gamma l}-k_{\mathrm{DT}}G_{D}$$

The dynamic equation for the ATP‐G‐actin concentration is given by the balance of the incoming recycling flux ${\text{flux}}_{\text{recycling}}=k_{\mathrm{DT}}\times G_{D}$ and outcoming assembly flux ${\text{flux}}_{\text{assembly}}=V$:

(5) $$\frac{{\mathrm{dG}}_{T}}{\mathrm{dt}}=k_{\mathrm{DT}}\times G_{D}-V$$

Note that according to equations (3), (4), (5) the total actin amount in the chamber, $\bar{G}=l+G_{D}+G_{T}$, is conserved (this becomes apparent if one adds equations 3, 4 and 5).

We measure both F‐actin and G‐actin concentrations in units of length. This is easy to envision in the case of the F‐actin in the tail. In the case of the G‐actin concentrations, there is a simple argument: If all available actin, $\bar{G}$, is assembled into a characteristic tail, then this tail's length will be equal to $l_{\max}$. Then, any concentration *G* measured in molar can be converted into length *l* measured in microns as follows: $l=\frac{l_{\max}}{\bar{G}}G$. Let us now discuss the assembly rate *V*. Recall that the maximal assembly rate $V_{0}=k_{\mathrm{on}}\delta \bar{G}\Phi$, or $V_{0}=\underset{\tilde{k}}{\underbrace{k_{\mathrm{on}}\Phi \delta \frac{\bar{G}}{l_{\max}}}}l_{\max}=\tilde{k}l_{\max}$. Here, $\tilde{k}=V_{0}/l_{\max}∼1\;\mathrm{\mu m}/\min/100\mathrm{\mu m}∼0.01/\min$. Then,

(6) $$V=k_{\mathrm{on}}\delta \Phi G_{T}=\tilde{k}G_{T}$$

where $G_{T}$ is now measured in units of length.

Equations (3), (4), (5), (6) define the actin dynamics characterized by a single stable dynamic steady state, in which three *fluxes* (number of actin subunits changing chemical state per unit time) are equal and balance each other: ${\text{flux}}_{\text{disassembly}}={\text{flux}}_{\text{assembly}}={\text{flux}}_{\text{recycling}}$. This leads to the formulas:

(7) $$\mathrm{\gamma l}=\tilde{k}G_{T}=k_{\mathrm{DT}}G_{D},\bar{G}=l_{\max}=l+G_{D}+G_{T}=\text{const}$$

From equation 7, $l=\frac{\tilde{k}}{\gamma }G_{T},\;G_{D}=\frac{\tilde{k}}{k_{\mathrm{DT}}}G_{T}.$ Substituting these into the conservation equation $l_{\max}=l+G_{D}+G_{T}=\text{const}$, we get: $G_{T}\left(1+\alpha +\beta \right)=l_{\max},\;\alpha =\frac{\tilde{k}}{k_{\mathrm{DT}}},\;\beta =\frac{\tilde{k}}{\gamma }$. From here, we obtain:

(8) $$G_{T}=\frac{l_{\max}}{1+\left(\alpha +\beta \right)},$$

(9) $$G_{D}=\frac{\alpha l_{\max}}{1+\left(\alpha +\beta \right)},$$

(10) $$l=\frac{\beta l_{\max}}{1+\left(\alpha +\beta \right)},$$

(11) $$V=\frac{\tilde{k}l_{\max}}{1+\left(\alpha +\beta \right)},$$

(12) $$\alpha =\frac{\tilde{k}}{k_{\mathrm{DT}}},\;\beta =\frac{\tilde{k}}{\gamma },\;\tilde{k}=k_{\mathrm{on}}\Phi \delta \frac{\bar{G}}{l_{\max}}.$$

Equations (8), (9), (10), (11), (12) allow comparison with the data.

##### Data on velocities and tail lengths in the “disassembly” and “recycling” cases allow rough estimates of the actin kinetics rates

In the “disassembly” case, the tail length rapidly increases at first, likely because it takes time for the disassembly machinery to start working and establishing the quasi‐steady state, which happens after about 2 h (Fig 2). After that, the tail length and velocity slowly (on ∼ 10 h scale) decrease due to the aging. Thus, we use the values of the velocity, ∼ 0.5 μm/min (Fig 2), and of the tail length, ∼ 20 μm established a few hours after the F‐actin growth starts (Fig EV2). This measured velocity is about twice smaller than the maximal velocity $\tilde{k}l_{\max}∼1\;\mathrm{\mu m}/\min$, and according to equation 11, $1+\left(\alpha +\beta \right)\approx 2$. The measured tail length is about threefold smaller than the maximal tail length in the assembly case, $l_{\max}∼60\;\mathrm{\mu m}$ (Fig EV2), and according to equation 10, $\beta /\left(1+\left(\alpha +\beta \right)\right)\approx 1/3$. From this, we deduce that α∼1/3,β∼2/3. Thus, $k_{\mathrm{DT}}=\tilde{k}/\alpha ∼0.03/\min,\gamma =\tilde{k}/\beta ∼0.015/\min$. This means that in the “disassembly” steady state, one‐third of actin is in the tail, half of it is in the ATP‐G‐actin form, and one‐sixth of it is in the ADP‐G‐actin form. The tail's F‐actin is disassembled in about 1 h, and G‐actin is recycled in about 30 min (more precise estimate, see below, suggests 1 h). This is in a good agreement with the fact that the actin tail length increases on an hour scale and then relaxes to the quasi‐steady value.

In the “recycling” case, the velocity during the first few hours is close to maximal, ∼ 1 μm/min (Fig 3), and the tail length is ∼ 10 μm (Fig EV2), 2 h after the start. According to equation 11, this means that $1+\left(\alpha +\beta \right)\approx 1$, and so values of both *α* and β are small. The measured tail length is about sixfold smaller than the maximal tail length in the assembly case, $l_{\max}∼60\;\mathrm{\mu m}$(Fig EV2), and according to equation 10, $\beta /\left(1+\left(\alpha +\beta \right)\right)\approx 1/6$. From this, we deduce that β∼1/6. Thus, $\gamma =\tilde{k}/\beta ∼0.06/\min$, and so the disassembly rate increases about fourfold in the “recycling” case: the tail's filaments disassemble, on average, in 15 min (more precise estimates that take aging effect into account suggest 10 min, see below). This agrees with the observation that the tail length in this case does not peak sharply in the beginning. The recycling rate is too fast to be estimated accurately, but it is likely to be at least an order of magnitude faster than that in the “disassembly” case, so G‐actin is recycled in minutes (more precise estimates that take aging effect into account suggest that G‐actin is recycled in 5 min or faster, see below). This means that in the “recycling” steady state, a small fraction of actin is in the tail, vast fraction of it is in the ATP‐G‐actin form, and a tiny fraction of it is in the ADP‐G‐actin form.

##### Fitting the data on velocities and tail lengths in the “disassembly” and “recycling” cases and including the aging effect into the model allows more precise estimates of the actin kinetics rates

We included the aging effect into the model by adding the aging term with respective rate ψ to equations (3), (4), (5), which now read:

(13) $$\begin{array}{l} \frac{\mathrm{dl}}{\mathrm{dt}}=V-\left(\gamma +\psi \right)l,\frac{{\mathrm{dG}}_{D}}{\mathrm{dt}}=\mathrm{\gamma l}-\left(k_{\mathrm{DT}}+\psi \right)G_{D},\\ \frac{{\mathrm{dG}}_{T}}{\mathrm{dt}}=k_{\mathrm{DT}}\times G_{D}-V-\psi G_{T}. \end{array}$$

(Note that strictly speaking the tail has still to disassemble with rate γ, after which some monomers are aged, and some are not, but because the rate of aging is an order of magnitude slower than that of disassembly, this does not lead to a significant error). We solved the system of equations 13 numerically and found the model parameters' values for which the fits to the data looked excellent. (The fitting procedure was ad hoc, by trying out a few tens of parameter sets.) The model results shown in the figures are the results of the numerical solutions with these optimal parameter values.

The following estimates of the parameters were obtained. In the disassembly case, the aging rate ψ≈0.002/min. In the recycling case, the aging rate ψ≈0.001/min. The F‐actin disassembly rate γ≈0.02/min in the disassembly case, so it takes about an hour to disassemble the tail. In the recycling case, γ≈0.12/min, so it takes about 10 min to disassemble the tail. The recycling rate $k_{\mathrm{DT}}\approx 0.02/\min$ in the disassembly case, so it takes about an hour to recycle a monomer after the disassembly. In the recycling case, any rate equal to $k_{\mathrm{DT}}\approx 0.2/\min$ or faster gives a good fit, so it takes less than several minutes (could be a minute, could be seconds) to recycle a monomer after the disassembly.

## Author contributions

**Laurent Blanchoin:** Conceptualization; funding acquisition; investigation; methodology; writing – original draft; project administration; writing – review and editing. **Manuel Thery:** Conceptualization; funding acquisition; investigation; methodology; writing – original draft; project administration; writing – review and editing. **Alexandra Colin:** Conceptualization; data curation; formal analysis; investigation; visualization; methodology; writing – original draft; writing – review and editing. **Tommi Kotila:** Resources; methodology. **Christophe Guérin:** Data curation; investigation; methodology. **Magali Orhant‐Prioux:** Data curation; investigation; methodology. **Benoit Vianay:** Data curation; project administration. **Alex Mogilner:** Conceptualization; formal analysis; funding acquisition; writing – original draft; writing – review and editing. **Pekka Lappalainen:** Resources; funding acquisition.

## Disclosure and competing interests statement

The authors declare that they have no conflict of interest.

## Supporting information



Expanded View Figures PDF

Movie EV1

Movie EV2

Movie EV3

Movie EV4

Movie EV5

Movie EV6

Movie EV7

Movie EV8

Movie EV9

Movie EV10

PDF+

## Data Availability

This study includes no data deposited in external repositories. The data that support the findings of this study are available from the corresponding author upon request.
