# Supplementary material for: Recycling of the actin monomer pool limits the lifetime of network turnover
Source: EMBO J. 2023 Mar 13;42(9):e112717. doi: 10.15252/embj.2022112717 (PMC10152149; doi:10.15252/embj.2022112717)
Supplement: Supplementary file 1 — Expanded View Figures PDF [file EMBJ-42-e112717-s004.pdf]

Expanded View Figures

Figure EV1. Microwells are closed environments that preserve key parameters for actin assembly.

A Top and side views of microwells used in this study.
B Snapshots of FRAP experiment in open (Top) and closed (Bottom) microwells.
C Quantification of FRAP experiment in open and closed microwell. Closed wells: $N = 3$, $n = 5$ microwells. Open wells: $N = 1$, $n = 2$ microwells. Mean and standard deviation are represented.
D Quantification of the association rate constant of actin filament assembly at the barbed ends in flow chamber and in microwells. Biochemical conditions: [actin] = 0.8 μM. [profilin] = 2.4 μM. $N = 2$, $n = 26$ filaments for the flow chamber and $n = 35$ filaments for the microwells. Individual points are represented (1 symbol per independent dataset) with mean and standard deviation superimposed.
E Visualization of actin branched network formation in closed microwells (full well and zoom). Biochemical conditions: [actin] = 1 μM. [profilin] = 3 μM. [WA] = 50 nM. [Arp2/3 complex] = 25 nM.
F Snapshots of actin comet tail grown in assembly conditions with labeled Arp2/3 complex (see Materials and Methods).
G Quantification of the Arp2/3 complex incorporated in the comet tail over the total quantity of the Arp2/3 complex in the microwell. Biochemical conditions of the experiment: 4.5 μm polystyrene beads coated with 400 nM SNAP-Strep-WA-His; 3 μM actin, 6 μM profilin, 90 nM Arp2/3 complex (labeled with Alexa647, see Materials and Methods), 15 nM capping protein. $N = 1$, $n = 16$ comet tails. Individual points are represented with mean and standard deviation superimposed.

© 2023 The Authors

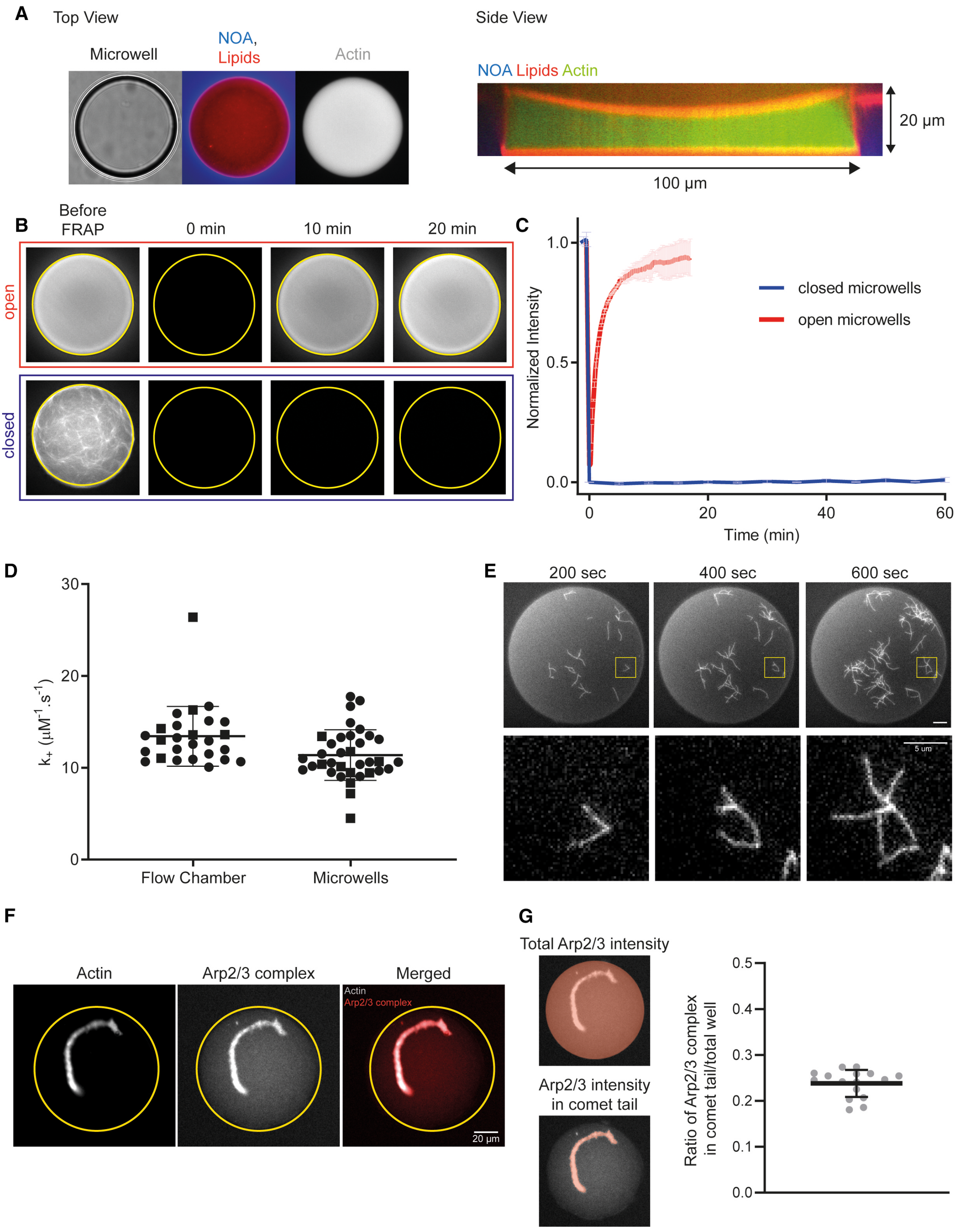

Figure EV1.

 © 2023 The Authors

Figure EV2. Quantitative analysis of actin within the comets in assembly, disassembly, and recycling conditions.

A Estimation of length, area, and actin integrated density of the comet shown in Fig 2 for Assembly conditions.
B Estimation of length, area, and actin integrated density of the comet shown in Fig 2 for Disassembly conditions.
C Estimation of length, area, and actin integrated density of the comet shown in Fig 2 for Recycling conditions.
D Mean intensity (actin density) of comet tails for the different conditions reconstituted. Assembly: $N = 3$, 38 comet tails, Disassembly: $N = 3$, 50 comet tails, Recycling: $N = 3$, 45 comet tails. Each independent replicate is represented by a different symbol; mean and standard deviation are superimposed on top of each condition.

© 2023 The Authors

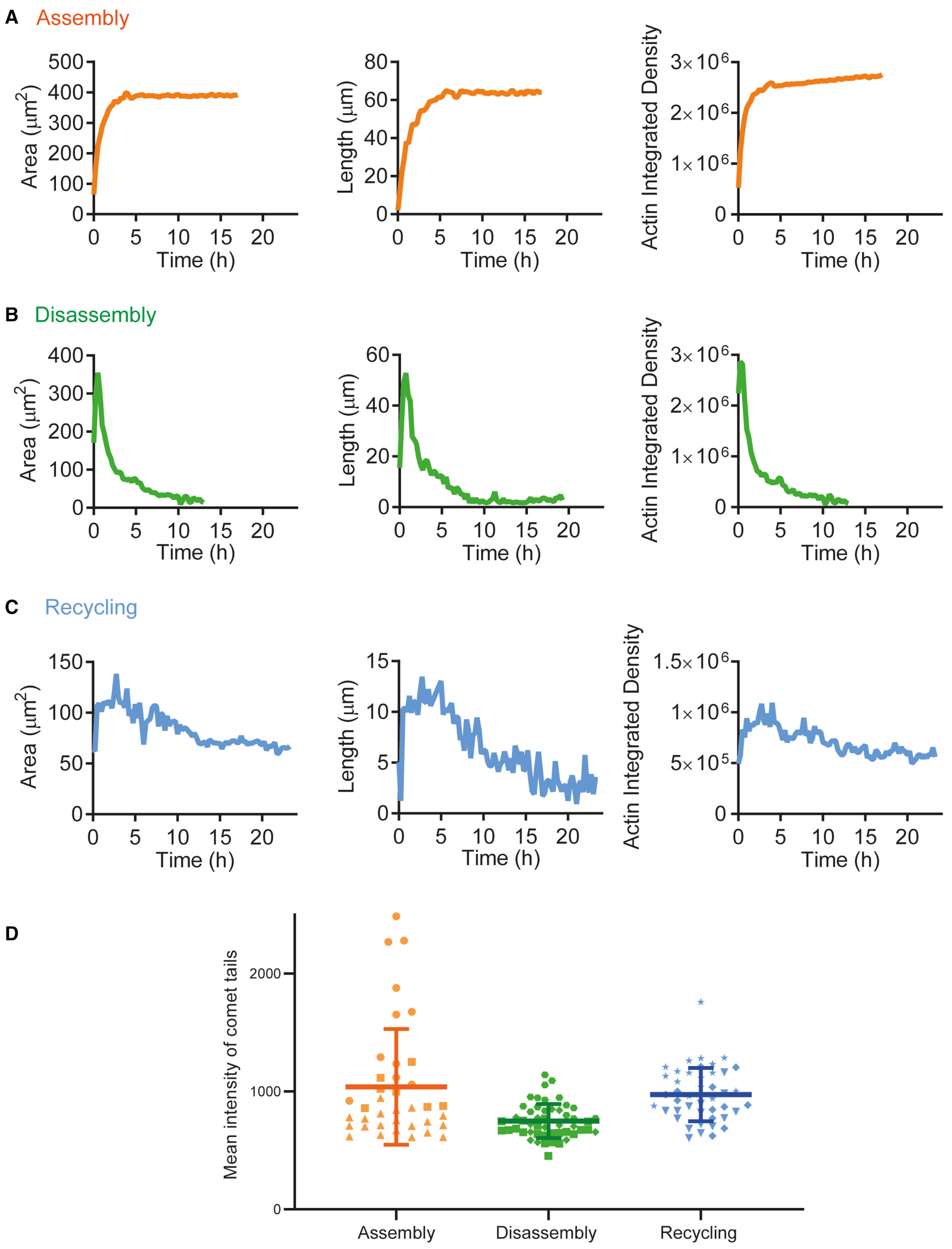

Figure EV2.

© 2023 The Authors

Figure EV3. Quantitative estimates of the experimental system under assembly, disassembly, and recycling conditions.

A Maximum number of times the initial pool of actin monomers was polymerized in the microwell for the different CAP constructs. Full-length: $N = 2$, $n = 18$ comet tails. C-CAP: $N = 2$, $n = 28$ comet tails. N-CAP: $N = 2$, $n = 20$ comet tails. Individual points are represented with mean and standard deviation superimposed. One-way ANOVA statistics: Full-length/C-CAP: ****P-value < 0.0001; Full-length/N-CAP: *P-value < 0.05. The gray dashed line represents 1 cycle which is equivalent to 3 μM, the initial concentration of actin introduced in the microwell.

B–D determination of the rate of disassembly of the actin comets in assembly, disassembly, and recycling conditions. (B) Left: Snapshots of an actin comet tail assembled in Assembly conditions in a microwell. The bead is in orange and the tracked defect in red. Right: Defect fluorescence intensity as a function of time (solid line) and exponential fit (dashed line). Time constant is estimated from the exponential fit. (C) Left: Snapshots of an actin comet tail assembled in Disassembly conditions in a microwell. The bead is in green and the tracked defect in red. Right: Defect fluorescence intensity as a function of time (solid line) and exponential fit (dashed line). Time constant is estimated from the exponential fit. (D) Left: Snapshots of an actin comet tail assembled in Recycling conditions in a microwell. The bead is in blue and the tracked defect in red. Right: Defect fluorescence intensity as a function of time (solid line) and exponential fit (dashed line). Time constant is estimated from the exponential fit.

E Comet disassembly time in disassembly ($N = 2$, $n = 3$ comets) and recycling ($N = 2$, $n = 3$ comets) conditions. Individual points for each comet are represented with mean and standard deviation superimposed.

F Speed of comet disassembly in disassembly ($N = 2$, $n = 3$ comets) and recycling conditions ($N = 2$, $n = 3$ comets). Individual points for each comet are represented with mean and standard deviation superimposed.

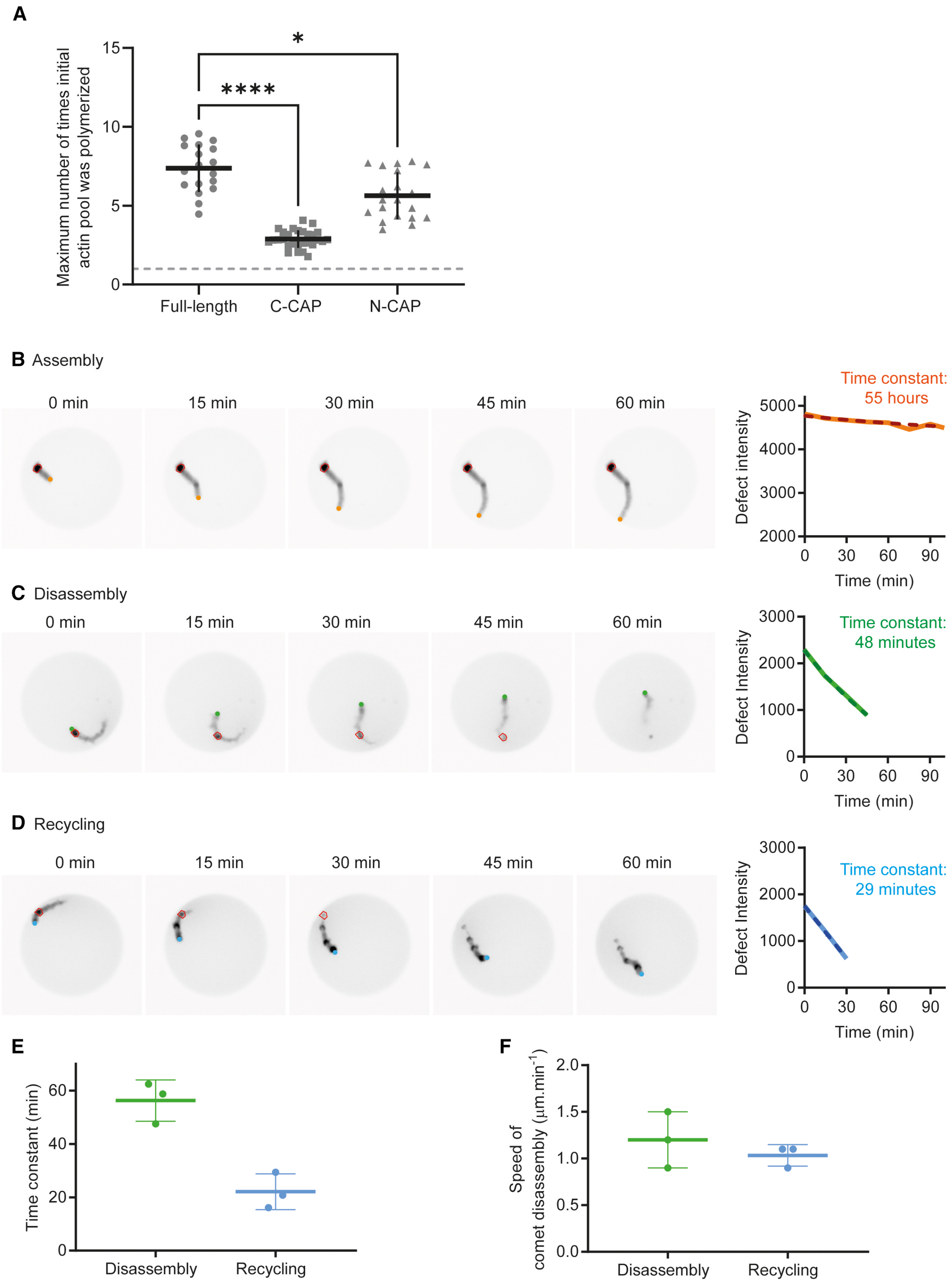

Figure EV3.

 © 2023 The Authors

Figure EV4. Effect of ATP concentration on actin-based motility in assembly and disassembly conditions.

A Snapshots of actin comet tails in assembly conditions with low ([ATP] = 0.007 mM) or high ([ATP] = 3 mM) ATP concentrations.
B Quantification of bead velocity for one dataset per condition in assembly conditions for low ([ATP] = 0.007 μM) or high ([ATP] = 3 mM) ATP concentrations. [ATP] = 0.007 μM: 7 comet tails. [ATP] = 3 mM: 11 comet tails.
C Quantification of comet area for one dataset per condition in Assembly conditions.
D Snapshots of actin comet tails in disassembly conditions with low ([ATP] = 0.007 mM) or high ([ATP] = 3 mM) ATP concentrations.
E Quantification of bead velocity for one dataset per condition in disassembly conditions for low ([ATP] = 0.007 μM) or high ([ATP] = 3 mM) ATP concentrations. [ATP] = 0.007 mM: 10 comet tails. [ATP] = 3 mM: 16 comet tails.
F Quantification of comet area for one dataset per condition.
G Number of times initial actin quantity was polymerized in the microwell for various concentrations of ATP in assembly or disassembly conditions. The gray dashed line represents 1 cycle which is equivalent to 3 μM, the initial concentration of actin introduced in the microwell. Assembly, [ATP] = 0.007 mM: $N = 1$, $n = 7$ comet tails. Assembly, [ATP] = 3 mM: $N = 3$, $n = 38$ comet tails. Disassembly, [ATP] = 0.007 mM: $N = 1$, $n = 10$ comet tails. Disassembly, [ATP] = 3 mM: $N = 3$, $n = 52$ comet tails. Biochemical conditions: 4 μm polystyrene beads coated with 400 nM SNAP-Strep-WA-His; 3 μM actin, 6 μM profilin, 90 nM Arp2/3, 15 nM capping protein (Assembly), 200 nM ADF/cofilin (Disassembly). Individual points for each comet (1 symbol per independent dataset) are represented with mean and standard deviation superimposed.

© 2023 The Authors

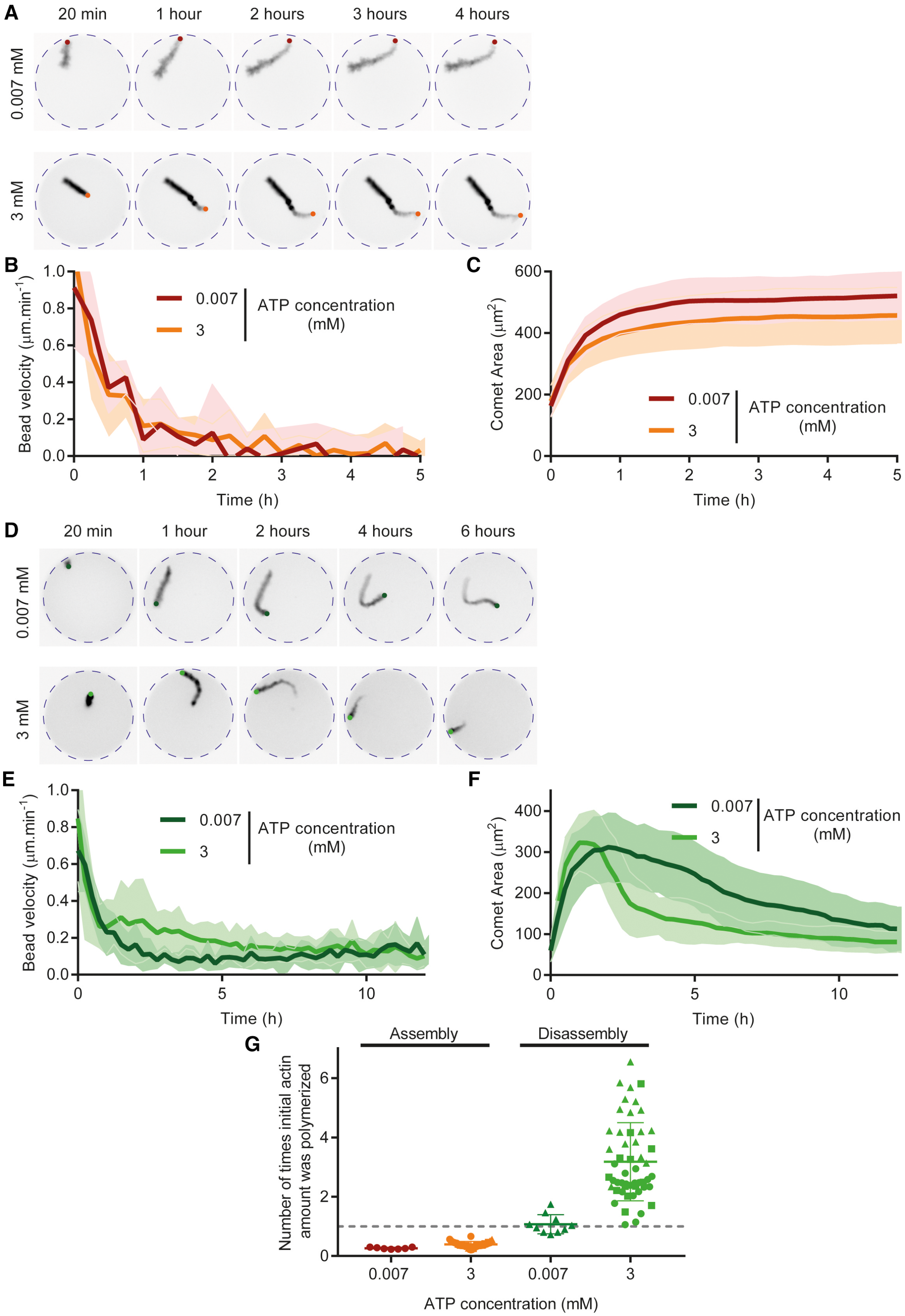

Figure EV4.

© 2023 The Authors

Figure EV5. Determination of the aging factor in our biochemical assay.

A Comparison of the number of times the initial actin pool was polymerized when the microwells were imaged by fluorescence or bright-field imaging only. Fluorescence imaging: $N = 4$, $n = 65$ comets. Bright-field only imaging: $N = 3$, $n = 28$ comets. Each independent replicate is represented by a different symbol; mean and standard deviation are superimposed on top of each condition. Unpaired t-test statistics: fluorescence/bright-field only: *P-value = 0.016.

B Comparison of bead velocity in flow chamber and in microwells (one dataset in each condition, $n = 37$ comet tails in flow chamber; $n = 13$ comet tails in microwells).

C Effect of ATP addition in an aged reaction mix. $t = 0$: $N = 4$, $n = 494$ comets. $t = 24$ h + actin: $N = 2$, $n = 108$ comets. $t = 24$ h + ATP: $N = 1$, $n = 7$ comets. Individual points for each comet are represented with mean and standard deviation superimposed.

D Test of reaction mix aging without actin. Reaction mix was prepared without beads and without actin and left on the bench at room temperature. After 24 h, fresh beads and fresh actin monomers were added to the mix and bead velocity was estimated. Composition of the reaction mix: 6 μM profilin, 90 nM Arp2/3, 15 nM capping protein, 200 nM ADF/cofilin, 400 nM cyclase-associated protein (CAP). Fresh mix, fresh actin: $N = 2$, $n = 118$ comets. Old mix (without actin) + fresh actin: $N = 2$, 49 comets. Individual points for each comet are represented with mean and standard deviation superimposed.

E, F Test of ADF/Cofilin and CAP aging. Each protein was diluted in motility buffer and left on the bench at room temperature overnight. The morning after, the aged protein was added to the motility assay. Velocity of beads was estimated in the different conditions. Composition of the reaction mix: 3 μM actin, 6 μM profilin, 90 nM Arp2/3, 15 nM capping protein, 200 nM ADF/cofilin, 400 nM cyclase-associated protein (CAP). Fresh ADF/cofilin: $N = 1$, $n = 88$ comet tails. Aged ADF/cofilin: $N = 1$, $n = 81$ comet tails. Fresh CAP: $N = 2$, $n = 89$ comets. Aged CAP: $N = 1$, $n = 149$ comets. Individual points for each comet are represented with mean and standard deviation superimposed.

© 2023 The Authors

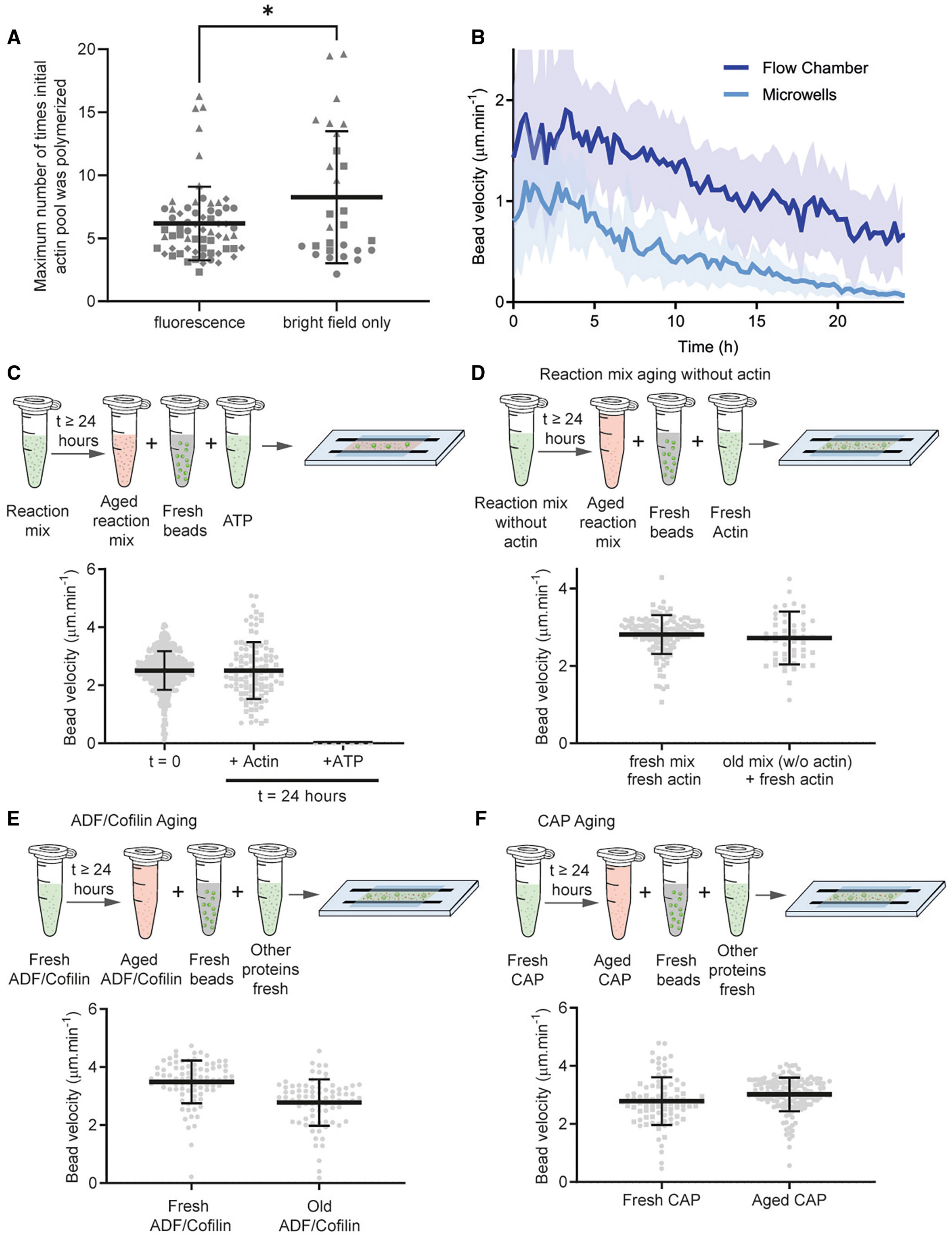

Figure EV5.

 © 2023 The Authors
