# Supplementary material for: Recycling of the actin monomer pool limits the lifetime of network turnover
Source: EMBO J. 2023 Mar 13;42(9):e112717. doi: 10.15252/embj.2022112717 (PMC10152149; doi:10.15252/embj.2022112717)
Supplement: Supplementary file 2 — Movie EV1 [file EMBJ-42-e112717-s013.zip › Movie EV1.docx]

## **Movie EV1 – Actin comet tail assembly in bulk and in microwells.**

Time lapse imaging of actin comet tail assembly in bulk and in microwells. Data is also shown in Figure 1D. Movie playback is 10 frames per second. Total elapsed time is 6 hours.
