# Supplementary material for: Recycling of the actin monomer pool limits the lifetime of network turnover
Source: EMBO J. 2023 Mar 13;42(9):e112717. doi: 10.15252/embj.2022112717 (PMC10152149; doi:10.15252/embj.2022112717)
Supplement: Supplementary file 3 — Movie EV2 [file EMBJ-42-e112717-s006.zip › Movie EV2.docx]

## **Movie EV2 – Contribution of assembly, disassembly and recycling on actin turnover in cell-sized compartment.**

Time lapse imaging of beads in microwells for various biochemical conditions (Assembly, Disassembly, Recycling). Data is also shown in Figure 2. Movie playback is 20 frames per second. Total elapsed time is 23 hours.
