# Supplementary material for: Recycling of the actin monomer pool limits the lifetime of network turnover
Source: EMBO J. 2023 Mar 13;42(9):e112717. doi: 10.15252/embj.2022112717 (PMC10152149; doi:10.15252/embj.2022112717)
Supplement: Supplementary file 5 — Movie EV4 [file EMBJ-42-e112717-s011.zip › Movie EV4.docx]

## **Movie EV4 – Examples of actin comet tails assembled in Disassembly conditions in microwells.**

Nine examples of time-lapse imaging of actin comet tail assembly in Disassembly conditions. Movie playback is 10 frames per second. Total elapsed time is 19 hours.
