# Supplementary material for: Recycling of the actin monomer pool limits the lifetime of network turnover
Source: EMBO J. 2023 Mar 13;42(9):e112717. doi: 10.15252/embj.2022112717 (PMC10152149; doi:10.15252/embj.2022112717)
Supplement: Supplementary file 7 — Movie EV6 [file EMBJ-42-e112717-s007.zip › Movie EV6.docx]

## **Movie EV6 – Variation of ATP concentration in Recycling conditions.**

Time lapse imaging of beads in microwells for various ATP concentrations in recycling conditions. Data is also shown in Figure 4. Movie playback is 20 frames per second. Total elapsed time is 20 hours.
