# Supplementary material for: Recycling of the actin monomer pool limits the lifetime of network turnover
Source: EMBO J. 2023 Mar 13;42(9):e112717. doi: 10.15252/embj.2022112717 (PMC10152149; doi:10.15252/embj.2022112717)
Supplement: Supplementary file 8 — Movie EV7 [file EMBJ-42-e112717-s005.zip › Movie EV7.docx]

## **Movie EV7 – Evaluation of beads aging.**

Time lapse imaging of beads left in buffer at room temperature nucleating actin comets in recycling conditions at different time points. Data quantification is shown in Figure 5A. Movie playback is 10 frames per second. Total elapsed time is 3 hours.
