# Supplementary material for: Recycling of the actin monomer pool limits the lifetime of network turnover
Source: EMBO J. 2023 Mar 13;42(9):e112717. doi: 10.15252/embj.2022112717 (PMC10152149; doi:10.15252/embj.2022112717)
Supplement: Supplementary file 9 — Movie EV8 [file EMBJ-42-e112717-s003.zip › Movie EV8.docx]

## **Movie EV8 – Evaluation of reaction mix aging.**

Time lapse imaging of beads added to reaction mix left at room temperature for aging at different time points. Data quantification is shown in Figure 5B. Movie playback is 10 frames per second. Total elapsed time is 3 hours.
