# Supplementary material for: Recycling of the actin monomer pool limits the lifetime of network turnover
Source: EMBO J. 2023 Mar 13;42(9):e112717. doi: 10.15252/embj.2022112717 (PMC10152149; doi:10.15252/embj.2022112717)
Supplement: Supplementary file 10 — Movie EV9 [file EMBJ-42-e112717-s008.zip › Movie EV9/Movie EV9.docx]

## **Movie EV9 – Addition of fresh components after 24 hours of aging.**

Time lapse imaging of beads added with fresh components to a reaction mix left at room temperature for aging during 24 hours. Data quantification is shown in Figure 5C. Movie playback is 7 frames per second. Total elapsed time is 120 minutes.
