# Supplementary material for: Recycling of the actin monomer pool limits the lifetime of network turnover
Source: EMBO J. 2023 Mar 13;42(9):e112717. doi: 10.15252/embj.2022112717 (PMC10152149; doi:10.15252/embj.2022112717)
Supplement: Supplementary file 11 — Movie EV10 [file EMBJ-42-e112717-s002.zip › Movie EV10/Movie EV10.docx]

**Movie EV10 – Evaluation of actin monomers aging.**

Time-lapse imaging of beads containing actin monomers with bound Ca-ATP or Mg-ATP, fresh or aged at room temperature for 24 hours. Data quantification is shown in Figure 5D. Movie playback is 7 frames per second. Total elapsed time is 160 minutes.
