# Supplementary material for: Recycling of the actin monomer pool limits the lifetime of network turnover
Source: EMBO J. 2023 Mar 13;42(9):e112717. doi: 10.15252/embj.2022112717 (PMC10152149; doi:10.15252/embj.2022112717)
Supplement: Supplementary file 13 [file EMBJ-42-e112717-s009.pdf]

THE EMBO JOURNAL

Recycling limits the lifetime of actin turnover

Alexandra Colin, Tommi Kotila, Christophe Guérin, Magali Orhant-Prioux, Benoit Vianay, Alex Mogilner, Pekka Lappalainen, Manuel Théry and Laurent Blanchoin
DOI: 10.15252/embj.2022112717

Corresponding author: Laurent BLANCHOIN (laurent.blanchoin@cea.fr) , Manuel Thery (manuel.thery@cea.fr)

Review Timeline:

Submission Date:	29th Sep 22
Editorial Decision:	21st Nov 22
Revision Received:	13th Jan 23
Editorial Decision:	9th Feb 23
Revision Received:	10th Feb 23
Accepted:	21st Feb 23

Editor: Ieva Gailite

Transaction Report:

(Note: With the exception of the correction of typographical or spelling errors that could be a source of ambiguity, letters and reports are not edited. Depending on transfer agreements, referee reports obtained elsewhere may or may not be included in this compilation. Referee reports are anonymous unless the Referee chooses to sign their reports.)

Thank you for submitting your manuscript for consideration by the EMBO Journal. I sincerely apologise for the protracted assessment of your manuscript due to delayed submission of referee reports. We have now received comments from three reviewers, which are included below for your information.

As you will see from the reports, the reviewers appreciate the work, while also indicating a number of aspects where they would require further improvement before they can support acceptance here, mostly pertaining to better characterisation of the experimental system. In addition, reviewer #1 requests further characterisation of the contribution from the other components of the reaction mix, such as CP and Arp2/3, a delineation of CAP functional contribution and recommends providing further analysis of actin aging. From my side, I find the reviewer comments generally reasonable and agree that adding the data in the directions indicated by reviewer #1 would strengthen the manuscript. Therefore, based on these positive assessments, I would like to invite you to address the issues raised by the reviewers in a revised manuscript. I would be happy to discuss the revision in more detail via email or phone/videoconferencing.

We generally allow three months as standard revision time. As a matter of policy, competing manuscripts published during this period will not negatively impact on our assessment of the conceptual advance presented by your study. However, please contact me as soon as possible upon publication of any related work to discuss the appropriate course of action. Should you foresee a problem in meeting this three-month deadline, please contact us to arrange an extension.

When preparing your letter of response to the referees' comments, please bear in mind that this will form part of the Review Process File and will therefore be available online to the community. For more details on our Transparent Editorial Process, please visit our website: https://www.embopress.org/page/journal/14602075/authorguide#transparentprocess. Please also see the attached instructions for further guidelines on preparation of the revised manuscript.

Please feel free to contact me if you have any further questions regarding the revision. Thank you for the opportunity to consider your work for publication, and I look forward to discussing your revision.

Referee #1:

Colin et al study the assembly and turnover of branched networks over long (hours) time scales in confinement. To this end, they develop micron-sized reaction chambers in which they monitor network assembly from encapsulated NPF-coated beads. They observe that, in the absence of network turnover, depletion of network constituents rapidly limits assembly. The authors increase the complexity of the reconstitution in a stepwise manner to include network disassembly and recycling, thereby closing the network turnover cycle. Turnover can sustained to a point where protein ageing becomes an issue, which is in itself an impressive feat. The molecular basis of long-term protein ageing remains somewhat unclear but is likely linked to actin oxidation.

The manuscript has many strong elements. The experiments are masterfully executed, the technical quality is very high and the presentation is very clear as characteristic for the Blanchoin/Thery lab. A weakness is that essentially all players used in the reconstitution have been characterized in great detail individually before. The degree of novel molecular insight is therefore a bit limited. On the other hand, this is the first time where continuous and rapid recycling has been reconstituted under boundary conditions similar to those in cells. As such, it constitutes an important step for more complex reconstitutions of cell motility in the future, which is of broad relevance. It also places the emphasis on considering the soluble actin pool; a much needed development in the cytoskeleton field. Overall, I consider it appropriate for the EMBO Journal, provided that some key points are addressed:

Major points:

1) The authors claim that the limiting component under confinement in the absence of network turnover/recycling is the actin monomer itself. However, also other network constituents (CP and Arp2/3) are consumed/sequestered in the process of network assembly. Especially the surprising observation that only half of the total actin is consumed under "assembly" conditions might argue against actin becoming limiting. Obtaining longer tails at higher actin concentrations does not necessarily mean that actin is limiting. Of course, one could elevate CP and Arp2/3 levels to test, but changing their concentrations has similar ambiguous effects on network composition and growth rate as modulating actin amounts. In my opinion, the authors should perform additional experiments to measure the degree of depletion for all network components under their "assembly" condition. Specifically, they should measure the remaining amounts of CP and Arp2/3 in solution outside of the network over time.

2) The microwell system developed is obviously very powerful. However, I feel that labeling them as "cell sized" (first in the abstract, but also throughout the paper) is somewhat misleading. Microwell volume (150pl) is still about 50-fold larger than the typical cell volume (3pl for a HeLa fibroblast). Of course cell size varies considerably, but the authors should clearly present and discuss these typical values.

More importantly, the authors should clearly state and relate two central quantities: a) the compartment volume and b) the reactive NPF-covered surface area on the bead, which spawns the actin network (I assume that they only analyzed microwells containing a single bead, but this not mentioned or described in the manuscript). What is the ratio between these quantities and how does this compare to the situation in e.g. motile cells, which generate branched networks at the leading edge? Some of the authors have done landmark modeling e.g on fish keratocytes, but the new experiments here are not put in perspective of this prior work.

3) The authors argue that "both acceleration of actin filament end depolymerization and nucleotide exchange on monomeric actin by CAP are important...". I agree that they have convincingly shown that CAP does indeed accelerate network disassembly (Suppl Fig. 3). However demonstrating that both activities are needed would require separation-of-function mutants of CAP, which are selectively impaired in promoting either filament depolymerization or monomer nucleotide exchange. Such mutants have been established in previous work by some of the authors (Kotila and Lappalainen), hence they should also be tested here. This would firmly establish whether indeed both CAP activities are indeed required and equally important.

4) The later parts of the paper concerning protein ageing are informative, but less complete compared the rest of the manuscript. Regarding the mechanism of ageing, it would be very informative to employ mass spectrometry on the aged reaction mix using a fresh one as an internal reference.

I was also surprised by the decision of the authors to monitor protein stability by high-speed centrifugation individually and at high concentration. Both of the latter changes might change stability/solubility. I suggest performing these assays in the complete motility mix in the absence of NPF-coated beads at the same concentration as used in the experiment (even actin should not significantly pellet under these conditions) and then probe their solubility by western blots.

Minor points:

- The choice of DTT as a reducing agent in long-term motility reactions is surprising, because it is easily the most short-lived and volatile among those normally used. This might be causally linked to long-term protein oxidation/ageing. I would recommend testing TCEP as an alternative.

- The distinction between the "disassembly" (profilin+cofilin) and the "recycling" (profilin+cofilin+CAP) conditions is less obvious than first introduced by the authors, because profilin should be able to also promote nucleotide exchange in actin as also explained by the authors later in the paper. I would consider introducing some of the overview schemes in Fig 6 early on, to illustrate that these are changes in degree but not complete absence/presence of recycling.

- Abstract: "Cellular actin networks consume matter and energy to sustain their dynamics...". While the energy aspect is rather obvious, I genuinely do not understand how actin networks consume matter.

- Abstract: I do not get the term "dynamic steady state". By my comprehension, any steady state is by definition "dynamic". The presence of net fluxes (aka broken detailed balance) distinguishes a steady state from an equilibrium.

- Abstract: ..."bead motility"... The general audience might not be familiar with this particular assay, so I would avoid field-specific jargon.

- Suppl. Figure 1A side view needs labels. What is labeled in green in the color merge?

- Results P6: "The disassembly step following the addition of ADF/cofilin allowed the bead to move for ~24 hours...". Only 12 hours are shown in the Figure.

- Results P6 and Figure 2E: The measure of "half-time of bead motility" is not defined or explained in the figure legend (had to go to the methods). Specifically the time course under "disassembly" conditions do not seem to be decaying mono-exponentially as also mentioned and explained by the authors later.

- Discussion P11: "This demonstrates that treadmilling limited by the rate of depolymerization at the pointed ends of the filament cannot account for rapid actin turnover". Filament pointed ends should be capped by the Arp2/3 complex under their experimental conditions, hence depolymerization from the pointed end should not occur.

Peter Bieling, MPI Dortmund

Referee #2:

The authors of "Recycling limits the lifetime of actin turnover" do an excellent job of introducing a new technology developed in their lab, namely use of microfabricated microwells, to address a long-standing hurdle in in vitro reconstitution. Using the microwell system, the authors address how a dynamic steady-state can be achieved in a cell-sized compartment with limiting reagents. This work is of great interest to the actin field and the in vitro reconstitution field, but also has a broad appeal in applications addressing how limiting components and competition for limited components affects biochemical reactions.

The authors developed a novel system that makes use of microfabricated microwells to generate a cell-sized compartment to investigate the factors required for establishment and maintenance of a dynamic steady state of actin assembly and disassembly with limited components. Furthermore, the authors then use this system to dissect what components in the system cause the

eventual limit on the recycling of components. The manuscript is well written with appropriate experiments and controls. The data is presented clearly and is interpreted appropriately. There are a few minor things that need to be addressed to improve the clarity of the manuscript, which are outlined below.

The authors first established the microwell system and confirmed that actin assembly is preserved inside the microwells with minimal components, NPR, arp2/3, actin, profilin and capping protein. Appropriate controls were completed confirming that rate of actin assembly at the barbed ends is the same in flow cell and microwell condition. Perhaps more filaments could be evaluated as only 13-17 filaments were evaluated. They also confirmed that the wells are sealed and components are consumed through the FRAP experiments.

I have some minor points that should be considered:

(1) In figure 1, D, the time should be indicated in the snapshots of actin comet growth as they are indicated for the flow chamber in figure 1C.
In figure 1B, different colors are used for the branched actin network close to and farther from the bead (tan vs. brown). If this is meant to represent newly assembled versus aged network, it should be specified (and also in figure 6).

(2) In the text for figure 1, more discussion occurs regarding the comet length and intensity, with less focus on the comet growth velocity, but later in the paper, more focus is on the comet velocity. This metric should be discussed in the discussion of results. Additionally, comet growth velocity is used in figure 1F, but bead velocity is used elsewhere in the paper- how are these measurements different and why is bead velocity not used in figure 1?

(3) Regarding figure 3, there are several assumptions used in the calculations that would benefit from further discussion and clarification. It is not clear why using the amount of actin assembled in the comet under assembly conditions as a factor to convert the length of action polymerized into a quantity of actin polymerized in the disassembly and recycling conditions is valid in these conditions. One might expect that the inclusion of ADF/ cofilin in the disassembly and recycling conditions might change the assembly conditions in these experiments. More discussion and elaboration of how the estimate of consumption of actin monomers in the system was determined and validated is needed. If there are technical limitations that impact the ability to make an accurate measurement of this, it should be addressed in the manuscript.

(4) For figure 3D, the mean for one representative experiment shown rather than the data for all the experiments as had been shown in other figures and in figure 3E. Based on figure 3E, it appears that the distribution of the maximum number of times the initial actin pool was polymerized is broad for disassembly and recycling conditions and as such, the range of values for the time course is of interest. The time course results from the other experiments should be included in a supplemental figure if it cannot be included in the main figure.

(5) The experiments with the ATP and evaluating the additional components in the reaction were well done. It would be interesting to evaluate and test the factors that the authors propose are contributing to actin aging, but it is reasonable that these experiments are outside the current scope and will be addressed in subsequent work.

(6) The model in figure 6 is somewhat confusing particularly the differences between the early disassembly conditions vs. the recycling conditions. The difference in arrow sizes is somewhat subtle. The model for the late conditions is much clearer. Additionally, this model is different than the models used in figure 2, which is maybe clearer (although the models in Figure 6 is more accurate, as there is some recycling with disassembly conditions, but it is not very efficient).

Referee #3:

Reconstituting actin turnover in vitro has been a challenge for many labs, a major obstacle being the quantification of what really limits the lifetime of any observed turn over. In the present study the authors overcome a number of obstacles by using microwells with a defined small volume of a 140 pl. Key points of the study are a minimal set of components and the limited availability of those components bringing it closer to a cell-like system. The manuscript demonstrates that with a limited amount of monomers, a three-step cycle is required to achieve prolonged actin dynamics. The authors have well determined what are some of the limiting factors for the system's functionality. Combination of a series of quantitative experiments allows them to identify the limit of long time stability of actin turn over, with Actin stability to be the major culprit for lack of stability of the actin turnover system.
The manuscript is very well written and key findings are clearly presented. I strongly recommend the acceptance of the manuscript as it advances our knowledge of in vitro systems significantly. I would suggest the authors to consider some minor points, which may help to strengthen the manuscript:

- The authors estimate the amount of consumed actin monomers by assuming that the bead velocity with cofilin would produce

the same amount or length of actin compared to the assembly conditions. The comet produced by the disassembly or recycling conditions seems to be thicker and denser though. Additionally, the conditions in Supp. Fig. 4A-C produce a different comet area even though the bead velocity is the same. A control showing that the bead velocity in different experiments is equal to the actin polymerization and therefore to the integrated actin intensity in the comet could be instructive to appreciate the accuracy of the values for the number of times actin was consumed.

- The aging of proteins was analyzed in the flow chamber setup and discussed as being caused by reactive oxygen species. Another cause could be a change in pH over the long imaging time caused by the ATP hydrolysis. This effect would be even more pronounced in the small volume of the microwells. Would it be possible to check this using e.g. microelectrodes or pH sensitive fluorescent dyes? Or estimate from the buffer capacity?

- The authors argue cys374 beeing responsible for the aging effect, I vaguely remember old discussions about DTT necessary to keep actin monomers stable in stock. Would it be possible use an adequate anti-oxidation system in the buffer? Do the authors have any indication that the oxygen radicals formed by fluorescence imaging affects the lifetime of the system?

- The authors compare the small volume of 140 pL in the microwells (which is still an order of magnitude larger than a human cell) to the large volume of the flow chamber. To appreciate the difference in monomers available between these compartments, it would be important to know the ratio of beads per volume instead of just comparing the volumes in these experiments.

- The authors describe a system in which the small confined volume leads to a more robust dynamic steady state. This can be achieved in different ways by means of adding different actin binding proteins as well. Bleicher et al. (Scientific Reports 2020) were able to produce a robust dynamic Arp2/3 network from beads in a large confinement by coupling it to actin polymerization by formins.

Some details:
- To my knowledge, the name ADF/Cofilin is used to describe the whole protein family - clearly the author is the expert on this. Yet, would it be more accurate to just use cofilin as the protein name in the manuscript and also specify in the results which cofilin is used.
- Fig2: Description of F says that it is "same as panel C", while most likely it is referring to panel D.
- Fig3B: It would be easier to compare the time values, if the x-axis would be in hours like in 3A and D.
- In figure 4 B and C colors used to depict the standard deviation affect the color of the mean, which makes reading the figure reading slightly confusing.
- Word "rince" is present all over the text instead of the word "rinse".
- In the section protein purification used "Rosettas 2", while likely "Rosetta 2" was intended.
- In the SUV preparation part both DOPE-Atto390 are Atto-647N mentioned and it is unclear which one was actually used.
- A very small point: I got first a bit confused, to which condition exactly the recycling condition refers to - it is mentioned in a paranthesis for Figure 2C - but maybe the authors add this explicitly into the sentence outside the parenthesis.

Rebuttal letter: **Colin et al. MS: EMBOj-2022-112717R**

Referee #1:

Colin et al study the assembly and turnover of branched networks over long (hours) time scales in confinement. To this end, they develop micron-sized reaction chambers in which they monitor network assembly from encapsulated NPF-coated beads. They observe that, in the absence of network turnover, depletion of network constituents rapidly limits assembly. The authors increase the complexity of the reconstitution in a stepwise manner to include network disassembly and recycling, thereby closing the network turnover cycle. Turnover can sustained to a point where protein ageing becomes an issue, which is in itself an impressive feat. The molecular basis of long-term protein ageing remains somewhat unclear but is likely linked to actin oxidation.

The manuscript has many strong elements. The experiments are masterfully executed, the technical quality is very high and the presentation is very clear as characteristic for the Blanchoin/Thery lab. A weakness is that essentially all players used in the reconstitution have been characterized in great detail individually before. The degree of novel molecular insight is therefore a bit limited. On the other hand, this is the first time where continuous and rapid recycling has been reconstituted under boundary conditions similar to those in cells. As such, it constitutes an important step for more complex reconstitutions of cell motility in the future, which is of broad relevance. It also places the emphasis on considering the soluble actin pool; a much needed development in the cytoskeleton field. Overall, I consider it appropriate for the EMBO Journal, provided that some key points are addressed:

Major points:

1) The authors claim that the limiting component under confinement in the absence of network turnover/recycling is the actin monomer itself. However, also other network constituents (CP and Arp2/3) are consumed/sequestered in the process of network assembly. Especially the surprising observation that only half of the total actin is consumed under "assembly" conditions might argue against actin becoming limiting. Obtaining longer tails at higher actin concentrations does not necessarily mean that actin is limiting. Of course, one could elevate CP and Arp2/3 levels to test, but changing their concentrations has similar ambiguous effects on network composition and growth rate as modulating actin amounts. In my opinion, the authors should perform additional experiments to measure the degree of depletion for all network components under their "assembly" condition. Specifically, they should measure the remaining amounts of CP and Arp2/3 in solution outside of the network over time.

We thank the reviewer for these comments to which we have responded with additional data.

1. Experimental observations

For Capping Protein, we know that it is not becoming limiting over the time course of the experiment because we do not see the appearance of fishbones patterns as can be observed when capping protein is limited (Pantaloni et al. NCB 2000; Achard et al., Current Biology, 2010).

For the Arp2/3 complex, we performed additional experiments in microwells using labelled Arp2/3 complex. As suggested by the reviewer, we measured the proportion of the Arp2/3 complex in the comet tail over the total amount of Arp2/3 complex in the microwell. We found that on average 25% of the labelled Arp2/3 complex (**Figure below**) is in the comet tail meaning that there is about 75% of the initial Arp2/3 complex still available in the bulk of the microwell. This demonstrates that Arp2/3 complex is not limiting in assembly conditions.

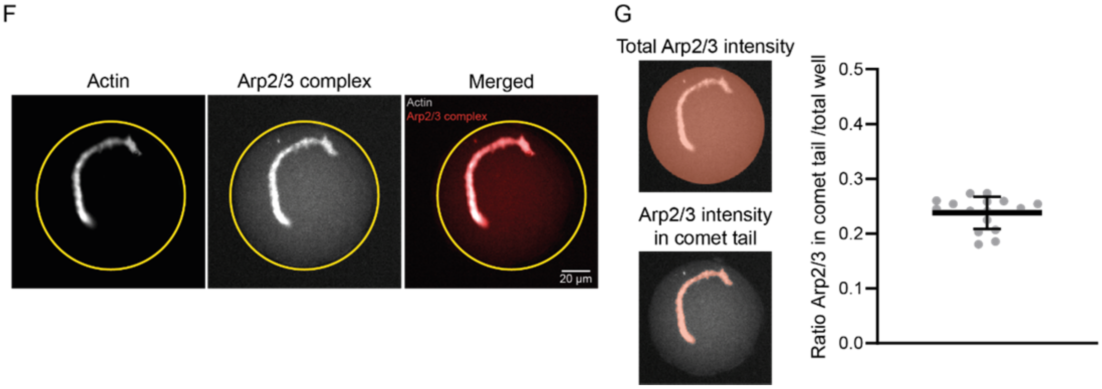

(Figure EV1) F. Snapshots of actin comet tail grown in assembly conditions with labelled Arp2/3 complex (see Methods). **G.** Quantification of Arp2/3 complex incorporated in the comet tail over the total quantity of Arp2/3 complex in the microwell. Biochemical conditions of the experiment: 4.5 µm polystyrene beads coated with 400 nM Strep-SNAP-WA; 3 µM actin, 6 µM profilin, 90 nM Arp2/3 complex (labelled with Alexa647, see methods), 15 nM capping protein. N = 1, n = 16 comet tails.

<u>2. Mathematical estimates.</u> We also made numerical estimations to confirm our experimental observations.

The microwell is a cylinder with radius $R = 50\mu m$ and height $H = 20\mu m$, so the volume of the chamber is $W = \pi R^2 H \approx 3 \times (50\mu m)^2 \times 20\mu m = 1.5 \times 10^5 \mu m^3$. There are about 600 molecules in one cubic micron of a solution with $\sim 1\mu M$ concentration (to reflect that, we use parameter $\omega \approx 600 / (\mu M \cdot \mu m^3)$), so the microwell contains $\omega \times W \times 3\mu M \approx 3 \times 10^8$ actin subunits. About 50% of actin subunits are assembled into the actin tail in the "assembly" case, so $\sim 1.5 \times 10^8$ actin subunits are in the longest tail. Thus, the total length of all filaments in the tail is $1.5 \times 10^8 \times 2.7 nm \approx 4 \times 10^5 \mu m$. The mean filament length observed in branched networks in vitro and in vivo is ~300 nm (Bieling et al. Cell 2016, Vinzenz et al. JCS 2012). Therefore, there is about 1.3×10^6 filaments in the comet tail.

Capping protein concentration is 15 nM, this represents 1.5×10^6 molecules of Capping protein. Arp2/3 complex concentration is 90 nM, this represents 10^7 molecules of Arp2/3.

If we consider one Arp2/3 complex and one capping protein per filament in the comet tail, we see from the number of molecules that there is enough proteins so that Capping protein and Arp2/3 complex are not depleted over the time of the experiment.

This represents 13% of the total number of Arp2/3 complex in the comet tail. This number is on the same order of magnitude as the one found experimentally.

In conclusion, the experimental observations and the numerical estimations confirm that Arp2/3 complex and Capping Protein are not globally depleted over the time course of the experiment in assembly conditions.

We added the numerical estimations in the “quantitative estimations” part and the quantification of the experiment with labelled Arp2/3 complex in Supplementary Figure 1.

2) The microwell system developed is obviously very powerful. However, I feel that labeling them as "cell sized" (first in the abstract, but also throughout the paper) is somewhat misleading. Microwell volume (150pl) is still about 50-fold larger than the typical cell volume (3pl for a HeLa fibroblast). Of course cell size varies considerably, but the authors should clearly present and discuss these typical values.

More importantly, the authors should clearly state and relate two central quantities: a) the compartment volume and b) the reactive NPF-covered surface area on the bead, which spawns the actin network (I assume that they only analyzed microwells containing a single bead, but this not mentioned or described in the manuscript). What is the ratio between these quantities and how does this compare to the situation in e.g. motile cells, which generate branched networks at the leading edge? Some of the authors have done landmark modeling e.g on fish keratocytes, but the new experiments here are not put in perspective of this prior work.

We thank the reviewer for this insightful comment. We have toned down the argument about cell size in the abstract even though (see discussion below), the reconstituted system presented here is very close to cellular conditions.

We have now added to the discussion (and below) a paragraph on the relevance of our reconstructed system to the cellular situation.

How does this reconstituted *in vitro* system compare to the situation in a living cell? For example, in keratocytes and fibroblasts moving on a 2D surface, most actin polymerization takes place at the leading edge (Theriot & Mitchison, 1991). The leading edge is ~ 30 µm wide and ~ 0.2 - 0.4 µm high, so its surface area is ~ 10 μm^2. In comparison, a 4.5 µm bead has a surface on which polymerization takes place of the same order of magnitude. Thus, the actin assembly assay presented in this study is similar to that of the cell. However, in the cell, the total concentration of actin is ~ 2 orders of magnitude higher than in the microwells (Pollard *et al*, 2000; Raz-Ben Aroush *et al*, 2017; Funk *et al*, 2019), but the volume of the microwells is almost 2 orders of magnitude higher than that of the cell, so the total amount of actin in the microwells is comparable to that in the cell. (Note also that the length of the actin tail in the disassembly and recycling cases is on the order of a few microns to ten microns, which is similar to the width of lamellipodial networks in living cells.) Thus, the main significant difference between the reconstituted system and a living cell is that the diffusion of actin monomers and actin-binding proteins in a larger volume could become limited, as we have shown in previous studies (Boujemaa-Paterski *et al*, 2017). However, the diffusion time is proportional to the square of the cell/chamber size. In this study, the characteristic size of the microwell is ~ 100 µm and that of the motile living cell is ~ 30 µm, so that the diffusion time in our case is at most ~ 10 times larger than in the cell. However, considering that in the living cell diffusion is slowed by cytoplasmic crowding absent *in vitro*, and that the flatness of the lamellae in living cells further slows the diffusive flow, our *in vitro* conditions quantitatively match those in living cells.

3) The authors argue that "both acceleration of actin filament end depolymerization and nucleotide exchange on monomeric actin by CAP are important...". I agree that they have convincingly shown that CAP does indeed accelerate network disassembly (Suppl Fig. 3). However demonstrating that both activities are needed would require separation-of-function mutants of CAP, which are

selectively impaired in promoting either filament depolymerization or monomer nucleotide exchange. Such mutants have been established in previous work by some of the authors (Kotila and Lappalainen), hence they should also be tested here. This would firmly establish whether indeed both CAP activities are indeed required and equally important.

We thank the reviewer for this suggestion. Indeed, what we call the recycling step consists in switching from the ADP loaded actin fragments generated by the ADF/cofilin-mediated disassembly of comet tails to the ATP-actin monomers to perform a new polymerization cycle. To better understand this recycling step, we performed experiments with mutants with separate CAP functions: the N-CAP mutant involved in the depolymerization of small oligomers into monomers; and the C-CAP mutant involved in nucleotide exchange on monomers. By looking at the number of times the initial pool of actin was polymerized in presence of the C-CAP or N-CAP mutant, we found that both activities are necessary for the recycling step. We now introduced those results in Supplementary Figure 3A and refer to them in the text:

Because CAP can both increase the rate of actin filament disassembly and nucleotide exchange on subunits, we used N-CAP and C-CAP known to respectively accelerate filament depolymerization and recharge ADP-actin monomers with ATP (Kotila *et al*, 2018, 2019). We saw that both activities were required for an efficient recycling (Supplemental Figure 3A).

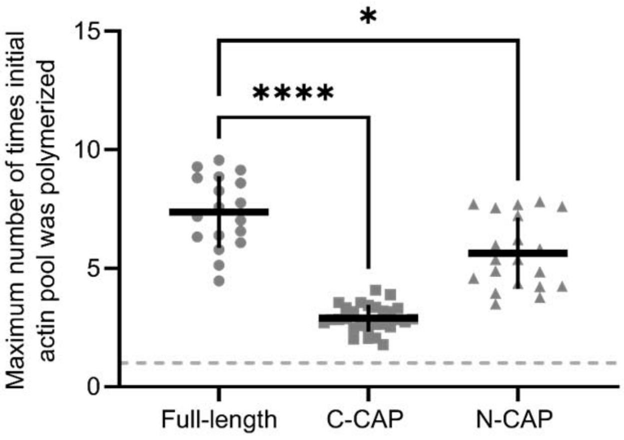

(Figure EV3) A. Maximum number of times the initial pool of actin monomers was polymerized in the microwell for the different constructions of CAP. Full-length: N = 2, n= 18 comet tails. C-CAP: N = 2, n = 28 comet tails. N-CAP: N = 2, n= 20 comet tails. 1 symbol per independent dataset. One-way ANOVA statistics: Full-length/C-CAP: p-value<0.0001 ****; Full-length/N-CAP: p-value <0.05 *.

4) The later parts of the paper concerning protein ageing are informative, but less complete compared the rest of the manuscript. Regarding the mechanism of ageing, it would be very informative to employ mass spectrometry on the aged reaction mix using a fresh one as an internal reference.

I was also surprised by the decision of the authors to monitor protein stability by high-speed centrifugation individually and at high concentration. Both of the latter changes might change stability/solubility. I suggest performing these assays in the complete motility mix in the absence of

NPF-coated beads at the same concentration as used in the experiment (even actin should not significantly pellet under these conditions) and then probe their solubility by western blots.

We thank the reviewer for this comment that we have now addressed in different ways:

We agree with the reviewer that the pelleting assay was not the best for monitoring protein stability over time. We have removed it from the manuscript and added experiments (see below) that specifically address component aging. Regarding the exact mechanism of aging, we believe it is beyond the scope of this study, although we have now identified the divalent cation bound (Mg^{2+} or Ca^{2+}) to actin monomers playing a key role during aging.

1. We aged the reaction mix with all the proteins. After 24 hours, we added fresh beads and individual fresh proteins. We found that addition of actin monomers is the only condition where we can restore bead motility.

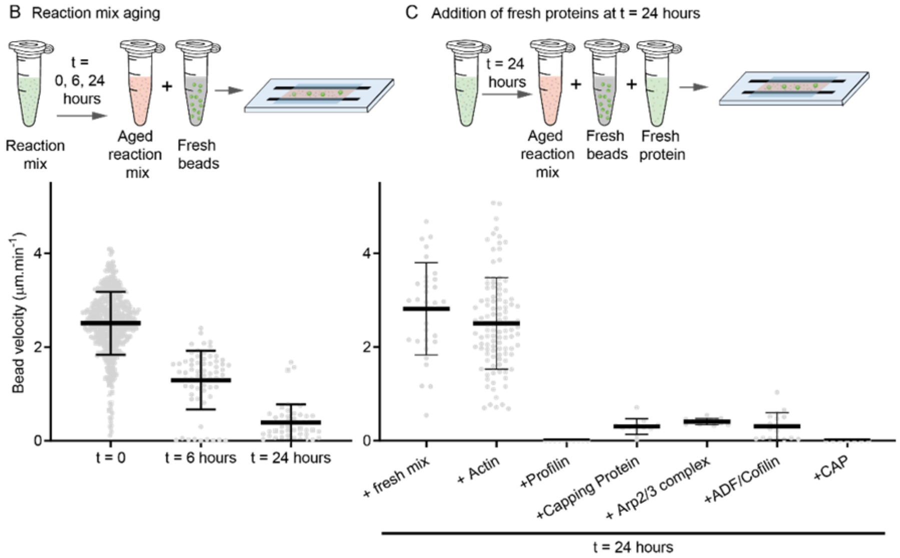

(Figure 5) B, C. Test of reaction mix aging without beads. Reaction mix was prepared without beads and left on the bench at room temperature. Then at different time intervals, fresh beads were added to the mix. After 24 hours (right part of the graph), all the (fresh) proteins of the assay were added individually with the beads. Velocity of fresh beads was estimated for those different times. Composition of the reaction mix: 3 µM actin, 6 µM profilin, 90 nM Arp2/3, 15 nM Capping protein, 200 nM cofilin, 400 nM Cyclase Associated Protein (CAP). t = 0: N = 4, n = 494 comets. t = 6 hours: N = 1, n = 65 comets. t = 24 hours: N = 2, n = 46 comets. t = 24 hours + fresh mix: N = 1, n = 32 comets. t = 24 hours + actin: N = 2, n = 108 comets. t = 24 hours + profilin: N = 1, n = 10 comets. t = 24 hours + capping protein: N = 1, n = 12 comets. t = 24 hours + Arp2/3 complex: N = 1, n = 11 comets. t = 24 hours + ADF/cofilin: N = 1, n = 17 comets. t = 24 hours + CAP: N = 1, n = 7 comets.

2. We also added an experiment where we aged the reaction mix without actin monomers. The addition of fresh actin monomers induced activity similar to a fresh mixture regarding bead motility. This demonstrates that aging of actin monomers is the main cause of the loss of motility mixture activity.

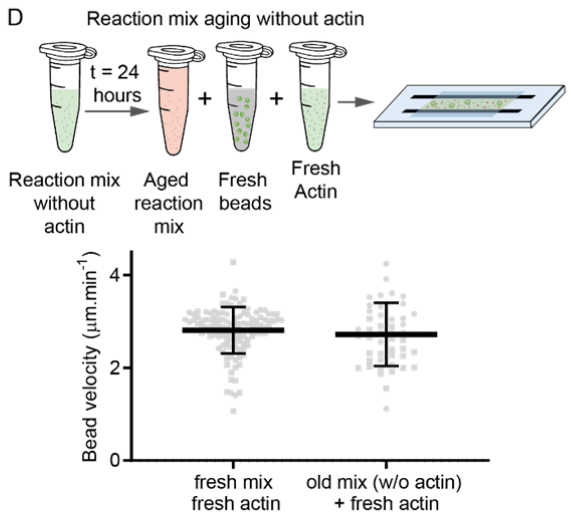

(Figure EV5) D. Test of reaction mix aging without actin. Reaction mix was prepared without beads and without actin and left on the bench at room temperature. After 24 hours, fresh beads and fresh actin monomers were added to the mix and bead velocity of fresh beads was estimated. Composition of the reaction mix: 3 µM actin, 6 µM profilin, 90 nM Arp2/3, 15 nM Capping protein, 200 nM cofilin, 400 nM Cyclase Associated Protein (CAP). Fresh mix, fresh actin: N = 2, n = 118 comets. Old mix (without actin) + fresh actin: N = 2, 49 comets.

3. Furthermore, the fact that when actin monomers were added to an old mixture, comet growth started as for a fresh mixture demonstrates that the assembly machinery (Profilin, Arp2/3 complex, capping protein) is still functional and therefore does not age significantly. In order to confirm that the disassembly machinery is not aging, we performed independently aging of ADF/cofilin and CAP proteins in the motility buffer. We found that none of these two protein significantly ages during the time course of the experiment.

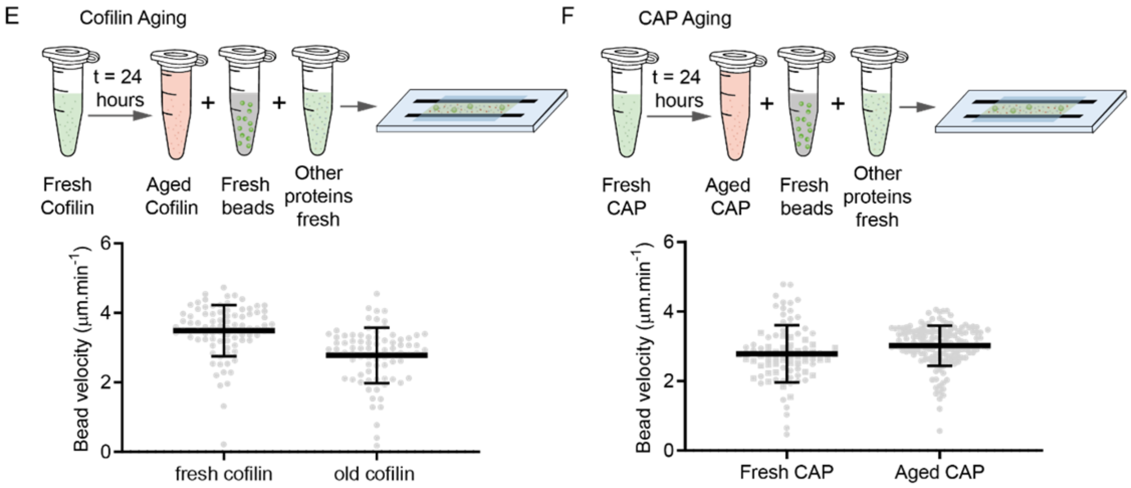

(Figure EV5) E, F. Test of ADF/Cofilin and CAP aging. Each protein was diluted in motility buffer and left on the bench at room temperature overnight. The morning after, the aged protein was added to the motility assay. Velocity of beads was estimated in the different conditions. Composition of the reaction mix: 3 µM actin, 6 µM profilin, 90 nM Arp2/3, 15 nM Capping protein, 200 nM cofilin, 400 nM Cyclase Associated Protein (CAP). Fresh cofilin: N = 1, n = 88 comet tails. Aged cofilin: N = 1, n = 81 comet tails. Fresh CAP: N = 2, n = 89 comets. Aged CAP: N = 1, n= 149 comets.

4. In order to better understand the cause of aging of actin monomers, we performed additional experiments that are now referred to as follows in the text:

Because Ca ATP-actin monomers are known to be stable for days in storage G-buffer after purification, these results were somewhat surprising. Therefore, we tested aging of Ca-ATP actin monomers in salt-free motility buffer to prevent polymerization. When added to the fresh motility mixture, the aged Ca-ATP actin monomers were able to fully restore bead motility (Figure 5D). Because an exchange of the divalent cation bound to the actin monomers from Ca2+ to Mg2+ occurs during actin polymerization, we tested the possibility that Mg-ATP actin monomers age more rapidly than Ca-ATP monomers. For that, we complemented the salt-free motility buffer with EGTA and MgCl2 (see Methods). Interestingly, the aged Mg-ATP monomers were unable to initiate bead motility demonstrating that aging of Mg-ATP actin monomers was the main factor responsible for the loss of motility mixture activity over time.

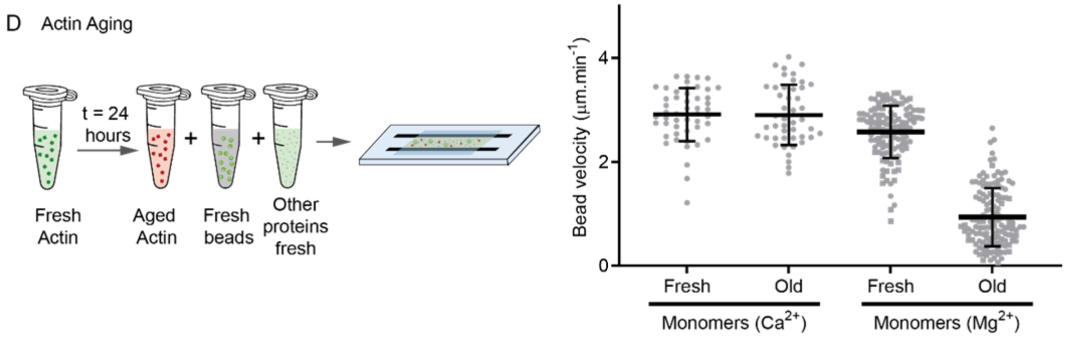

(Figure 5) D. Test of actin monomers aging. Actin monomers were prepared in salt-free motility buffer (Ca-ATP monomers, see Methods) or in salt-free motility buffer with EGTA and $MgCl_2$ (Mg-ATP monomers, see Methods) and left on the bench at room temperature overnight. The day after, the mix was added to fresh beads and fresh proteins (except actin). Velocity of beads was estimated in the different conditions. Composition of the reaction mix: 3 µM actin, 6 µM profilin, 90 nM Arp2/3, 15 nM Capping protein, 200 nM cofilin, 400 nM Cyclase Associated Protein (CAP). Ca-ATP actin monomers fresh: N = 1, n = 50 comets. Ca-ATP actin monomers (old) N = 1, n = 46 comets. Mg-ATP actin monomers (fresh): N = 2, n = 121 comets. Mg-ATP actin monomers (old): N = 2, n = 134 comets.

Minor points:

- The choice of DTT as a reducing agent in long-term motility reactions is surprising, because it is easily the most short-lived and volatile among those normally used. This might be causally linked to long-term protein oxidation/ageing. I would recommend testing TCEP as an alternative.

We agree with the reviewer that aging of DTT could explain potential protein oxidation as a cause of aging. We tried to use TCEP but it did not improve the lifetime of the experiment. In addition, we performed experiments without fluorescence imaging. In those experiments, we only used bright-field imaging. We found that the number of times the initial pool of actin monomers was polymerized was similar for both imaging conditions. We introduced those new results in Figure EV5.

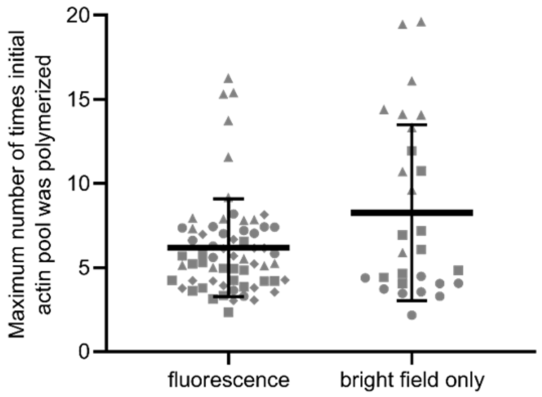

(Figure EV5) A. Comparison of number of times initial actin pool was polymerized when the experiment was imaged with fluorescence or with bright field imaging only. Fluorescence imaging: N = 4, n = 65 comets. Bright field only imaging: N = 3, n = 28 comets. Each independent replicate is represented by a different symbol; mean and standard deviation are superimposed on top of each condition.

- The distinction between the "disassembly" (profilin+cofilin) and the "recycling" (profilin+cofilin+CAP) conditions is less obvious than first introduced by the authors, because profilin should be able to also promote nucleotide exchange in actin as also explained by the authors later in the paper. I would consider introducing some of the overview schemes in Fig 6 early on, to illustrate that these are changes in degree but not complete absence/presence of recycling.

We understand the referee's point of view, but we prefer to first have a simple scheme to illustrate the main steps during the 3 conditions presented in Figure 2 and then complete this scheme in Figure 6 based on the findings of this study.

- Abstract: "Cellular actin networks consume matter and energy to sustain their dynamics...". While the energy aspect is rather obvious, I genuinely do not understand how actin networks consume matter.

We modified the abstract to make it clearer:

Intracellular organization is largely mediated by actin turnover. Cellular actin networks continually assemble and disassemble, while maintaining their appearance. This behavior, called "dynamic steady state," allows cells to sense and adapt to their environment.

- Abstract: I do not get the term "dynamic steady state". By my comprehension, any steady state is by definition "dynamic". The presence of net fluxes (aka broken detailed balance) distinguishes a steady state from an equilibrium.

We agree with this point. But (see below) we added the term “dynamic” to illustrate that the flux in and out equalize.

Indeed, dynamics can be either in thermodynamic equilibrium, in which case fluxes between any two states are equal, or not in thermodynamic equilibrium, which is our case (i.e. F-actin does not turn back into ATP-G-actin). So, in our case, it is not a thermodynamic equilibrium, but dynamic steady state. We would say that there are ‘dynamic’ and ‘static’ steady states. The difference is that in the dynamic one, there is a flux in and a flux out, which equalize. In the ‘static’ one, there are no fluxes. A good example is in our assembly case, we have a steady state which is more or less static: no more

assembly, and no disassembly. In our disassembly and recycling cases, the steady states are dynamic – there are significant equal fluxes of actin from one state to the other.

- Abstract: ..."bead motility"... The general audience might not be familiar with this particular assay, so I would avoid field-specific jargon.

We modified the abstract. The formulation is now the following:
To answer this question, we developed an experimental system where polystyrene beads are propelled by an actin comet in a microwell containing a limited amount of components.

- Suppl. Figure 1A side view needs labels. What is labeled in green in the color merge?

We modified Supplemental Figure 1 to make it clearer.

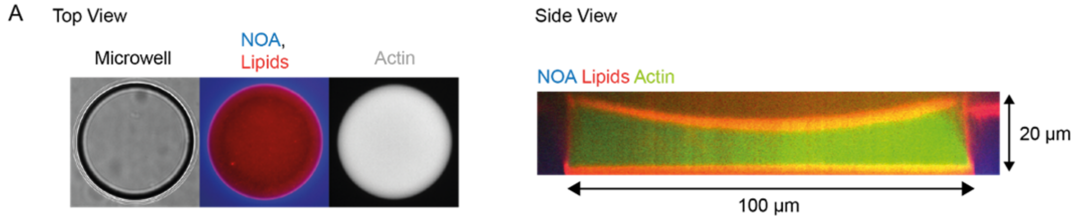

- Results P6: "The disassembly step following the addition of ADF/cofilin allowed the bead to move for ~24 hours...". Only 12 hours are shown in the Figure.

We only put images on 12 hours time-scale on the figure for clarity and space constraints. However, the movie 2 and the analysis (Figure 2D) are shown on a duration of 24 hours.

- Results P6 and Figure 2E: The measure of "half-time of bead motility" is not defined or explained in the figure legend (had to go to the methods). Specifically the time course under "disassembly" conditions do not seem to be decaying mono-exponentially as also mentioned and explained by the authors later.

We have modified the legend of Figure 2 to add more details on this point:

(Figure 2) E. Estimation of half-life of bead motility (from an exponential fit to the bead velocity curve)

- Discussion P11: "This demonstrates that treadmilling limited by the rate of depolymerization at the pointed ends of the filament cannot account for rapid actin turnover". Filament pointed ends should be capped by the Arp2/3 complex under their experimental conditions, hence depolymerization from the pointed end should not occur.

We have modified the sentence to mention end depolymerization and not go too much in detail.

Referee #2:

The authors of "Recycling limits the lifetime of actin turnover" do an excellent job of introducing a new technology developed in their lab, namely use of microfabricated microwells, to address a long-standing hurdle in in vitro reconstitution. Using the microwell system, the authors address how a dynamic steady-state can be achieved in a cell-sized compartment with limiting reagents. This work is of great interest to the actin field and the in vitro reconstitution field, but also has a broad appeal in applications addressing how limiting components and competition for limited components affects biochemical reactions.

The authors developed a novel system that makes use of microfabricated microwells to generate a cell-sized compartment to investigate the factors required for establishment and maintenance of a dynamic steady state of actin assembly and disassembly with limited components. Furthermore, the authors then use this system to dissect what components in the system cause the eventual limit on the recycling of components. The manuscript is well written with appropriate experiments and controls. The data is presented clearly and is interpreted appropriately. There are a few minor things that need to be addressed to improve the clarity of the manuscript, which are outlined below.

The authors first established the microwell system and confirmed that actin assembly is preserved inside the microwells with minimal components, NPR, arp2/3, actin, profilin and capping protein. Appropriate controls were completed confirming that rate of actin assembly at the barbed ends is the same in flow cell and microwell condition. Perhaps more filaments could be evaluated as only 13-17 filaments were evaluated. They also confirmed that the wells are sealed and components are consumed through the FRAP experiments.

We thank the reviewer for his/her comments. We added more filaments for the evaluation of the rate of actin assembly at the barbed end.

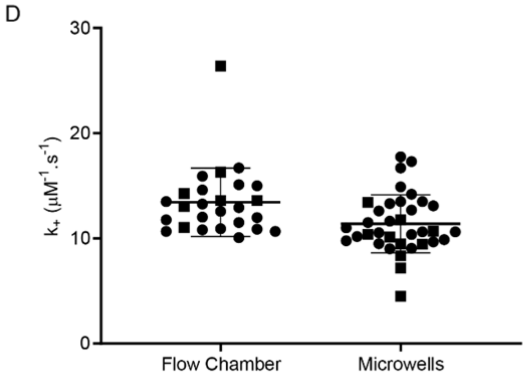

(Figure EV1) D. Quantification of the association rate constant of actin filament assembly at the barbed ends in flow chamber and in microwells. Biochemical conditions: [actin] = 0.8 µM. [profilin] = 2.4 µM. N = 2, n = 26 filaments for the flow chamber and n = 35 filaments for the microwells.

I have some minor points that should be considered:

We respond bellow point by point to the reviewer's comment.

(1) In figure 1, D, the time should be indicated in the snapshots of actin comet growth as they are indicated for the flow chamber in figure 1C.
In figure 1B, different colors are used for the branched actin network close to and farther from the bead (tan vs. brown). If this is meant to represent newly assembled versus aged network, it should be specified (and also in figure 6).

We modified Figure 1 and modified the figure legends of Figure 1 and Figure 6 to make them clearer.

(2) In the text for figure 1, more discussion occurs regarding the comet length and intensity, with less focus on the comet growth velocity, but later in the paper, more focus is on the comet velocity. This metric should be discussed in the discussion of results. Additionally, comet growth velocity is used in figure 1F, but bead velocity is used elsewhere in the paper- how are these measurements different and why is bead velocity not used in figure 1?

The growth velocity is an easy value to estimate from the data. In assembly conditions only, the growth velocity is equivalent to the bead velocity. Therefore, growth velocity reported in figure 1 is equivalent to bead velocity.

(3) Regarding figure 3, there are several assumptions used in the calculations that would benefit from further discussion and clarification. It is not clear why using the amount of actin assembled in the comet under assembly conditions as a factor to convert the length of action polymerized into a quantity of actin polymerized in the disassembly and recycling conditions is valid in these conditions. One might expect that the inclusion of ADF/ cofilin in the disassembly and recycling conditions might change the assembly conditions in these experiments. More discussion and elaboration of how the estimate of consumption of actin monomers in the system was determined and validated is needed. If there are technical limitations that impact the ability to make an accurate measurement of this, it should be addressed in the manuscript.

We looked at the average intensity of the comet tail under the different conditions reconstituted in our assay. We found that their average intensity does not change significantly between assembly, disassembly, or recycling, showing that assembly does not vary when disassembly and recycling proteins are added.

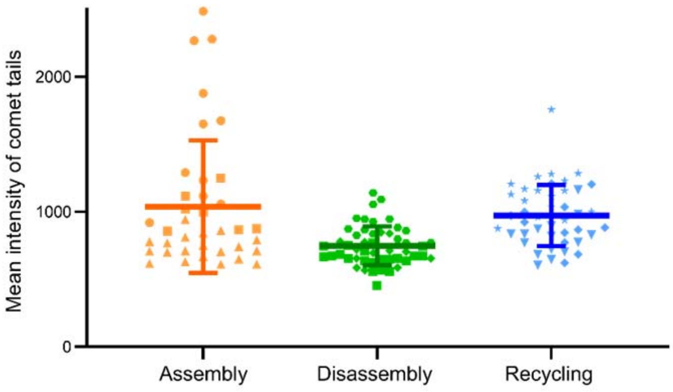

(Figure EV2) D. Mean intensity (actin density) of comet tails for the different conditions reconstituted. Assembly: N = 3, 38 comet tails, Disassembly: N = 3, 50 comet tails, Recycling: N = 3, 45 comet tails. Each

independent replicate is represented by a different symbol; mean and standard deviation are superimposed on top of each condition.

(4) For figure 3D, the mean for one representative experiment shown rather than the data for all the experiments as had been shown in other figures and in figure 3E. Based on figure 3E, it appears that the distribution of the maximum number of times the initial actin pool was polymerized is broad for disassembly and recycling conditions and as such, the range of values for the time course is of interest. The time course results from the other experiments should be included in a supplemental figure if it cannot be included in the main figure.

We modified Figure 3D to represent the mean over all the replicates.

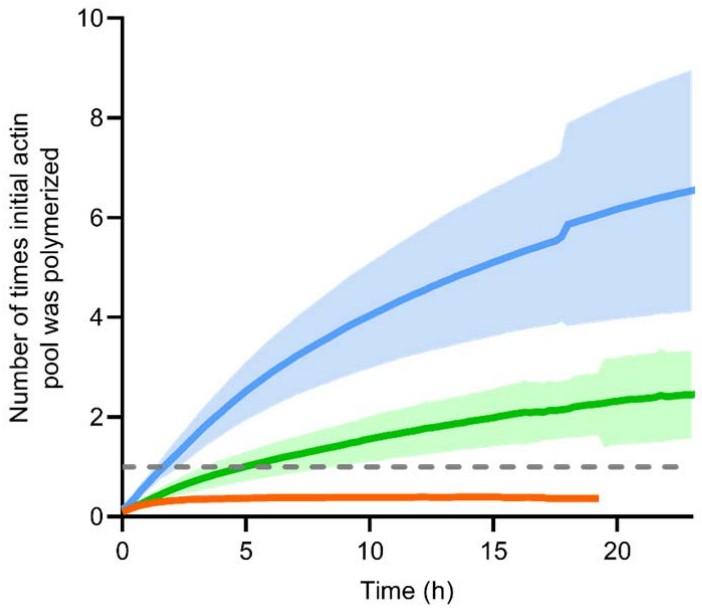

D. Mean number of times initial actin amount was polymerized as a function of time (mean and standard deviation are shown). Assembly: N = 3, n= 38 comet tails. Disassembly: N = 3, n = 52 comet tails. Recycling: N = 4, n= 65 comet tails.

(5) The experiments with the ATP and evaluating the additional components in the reaction were well done. It would be interesting to evaluate and test the factors that the authors propose are contributing to actin aging, but it is reasonable that these experiments are outside the current scope and will be addressed in subsequent work.

We agree with the point of the referee.

(6) The model in figure 6 is somewhat confusing particularly the differences between the early disassembly conditions vs. the recycling conditions. The difference in arrow sizes is somewhat subtle. The model for the late conditions is much clearer. Additionally, this model is different than the models used in figure 2, which is maybe clearer (although the models in Figure 6 is more accurate, as there is some recycling with disassembly conditions, but it is not very efficient).

We understand the point of the referee, but we prefer to have a simple scheme in Figure 2 to illustrate the main step during the 3 conditions presented and a more accurate description in Figure 6.

Referee #3:

Reconstituting actin turnover in vitro has been a challenge for many labs, a major obstacle being the quantification of what really limits the lifetime of any observed turn over. In the present study the authors overcome a number of obstacles by using microwells with a defined small volume of a 140 pl. Key points of the study are a minimal set of components and the limited availability of those components bringing it closer to a cell-like system. The manuscript demonstrates that with a limited amount of monomers, a three-step cycle is required to achieve prolonged actin dynamics. The authors have well determined what are some of the limiting factors for the system's functionality. Combination of a series of quantitative experiments allows them to identify the limit of long time stability of actin turn over, with Actin stability to be the major culprit for lack of stability of the actin turnover system.

The manuscript is very well written and key findings are clearly presented. I strongly recommend the acceptance of the manuscript as it advances our knowledge of in vitro systems significantly. I would suggest the authors to consider some minor points, which may help to strengthen the manuscript:

- The authors estimate the amount of consumed actin monomers by assuming that the bead velocity with cofilin would produce the same amount or length of actin compared to the assembly conditions. The comet produced by the disassembly or recycling conditions seems to be thicker and denser though. Additionally, the conditions in Supp. Fig. 4A-C produce a different comet area even though the bead velocity is the same. A control showing that the bead velocity in different experiments is equal to the actin polymerization and therefore to the integrated actin intensity in the comet could be instructive to appreciate the accuracy of the values for the number of times actin was consumed.

See response to point 3 of reviewer #2 where we show that actin mean intensity in comet tails does not change between the different conditions reproduced.

- The aging of proteins was analyzed in the flow chamber setup and discussed as being caused by reactive oxygen species. Another cause could be a change in pH over the long imaging time caused by the ATP hydrolysis. This effect would be even more pronounced in the small volume of the microwells. Would it be possible to check this using e.g. microelectrodes or pH sensitive fluorescent dyes? Or estimate from the buffer capacity?

This is an interesting point and change in pH is always a possibility. But the fact that the system restarts just by adding actin monomers demonstrates that if any change in pH is not the main cause for aging.

- The authors argue cys374 being responsible for the aging effect, I vaguely remember old discussions about DTT necessary to keep actin monomers stable in stock. Would it be possible use an adequate anti-oxidation system in the buffer? Do the authors have any indication that the oxygen radicals formed by fluorescence imaging affects the lifetime of the system?

See response to the first minor point of reviewer 1.

- The authors compare the small volume of 140 pL in the microwells (which is still an order of magnitude larger than a human cell) to the large volume of the flow chamber. To appreciate the difference in monomers available between these compartments, it would be important to know the ratio of beads per volume instead of just comparing the volumes in these experiments.

Following the advice of the reviewer, we made the following estimates which are now included in the materials and methods:

<u>Comparison of bead density in the flow chamber and in the microwells</u>

In microwells, there is on average 1 bead per microwell, meaning 1 bead/140 pL. In flow chamber, we observe around 20 beads per field of view (10X field of view). The estimated volume of the field of view is 120000 pL. Therefore, the bead density in the flow chamber is around 0.02 bead/140 pL. This means that the beads are 50 times more diluted in the flow chamber than in the microwell.

- The authors describe a system in which the small confined volume leads to a more robust dynamic steady state. This can be achieved in different ways by means of adding different actin binding proteins as well. Bleicher et al. (Scientific Reports 2020) were able to produce a robust dynamic Arp2/3 network from beads in a large confinement by coupling it to actin polymerization by formins.

We thank the reviewer for pointing out this paper. We have now included it in the introduction.

Some details:
- To my knowledge, the name ADF/Cofilin is used to describe the whole protein family - clearly the author is the expert on this. Yet, would it be more accurate to just use cofilin as the protein name in the manuscript and also specify in the results which cofilin is used.
As a standard in the field we use ADF/cofilin as a generic name to avoid confusion, so we hope this is ok if we continue to use the generic name.
- Fig2: Description of F says that it is "same as panel C", while most likely it is referring to panel D.
modified
- Fig3B: It would be easier to compare the time values, if the x-axis would be in hours like in 3A and D. modified
- In figure 4 B and C colors used to depict the standard deviation affect the color of the mean, which makes reading the figure reading slightly confusing.
- Word "rince" is present all over the text instead of the word "rinse". modified
- In the section protein purification used "Rosettas 2", while likely "Rosetta 2" was intended.
modified
- In the SUV preparation part both DOPE-Atto390 are Atto-647N mentioned and it is unclear which one was actually used. modified
- A very small point: I got first a bit confused, to which condition exactly the recycling condition refers to - it is mentioned in a paranthesis for Figure 2C - but maybe the authors add this explicitly into the sentence outside the parenthesis.

Thank you for submitting a revised version of your manuscript. Your study has now been seen by all original referees, who find that their previous concerns have been addressed and now recommend publication of the manuscript. There remain only a few editorial points that have to be addressed before I can extend formal acceptance of the manuscript .

Please let me know if you have any questions regarding any of these points. You can use the link below to upload the revised files.

Thank you again for giving us the chance to consider your manuscript for The EMBO Journal. I look forward to receiving the final version.

Referee #1:

The authors have comprehensively addressed all points raised in the original review. The new experiments and textual revisions have significantly improved the paper and I fully support its publication in EMBO J.

Referee #2:

The points that we brought up for consideration have been appropriately addressed and we have no additional concerns that require attention.

Referee #3:

I thank the authors for replying in depth to all raised points, and continue to support the publication of this excellent manuscript!

The authors addressed the remaining editorial issues.

Thank you for addressing the final editorial issues. I am now pleased to inform you that your manuscript has been accepted for publication in the EMBO Journal.

EMBO Press Author Checklist

Corresponding Author Name: Laurent Blanchoin	
Journal Submitted to: EMBO Journal	
Manuscript Number: EMBOJ-2022-112717	

USEFUL LINKS FOR COMPLETING THIS FORM
The EMBO Journal - Author Guidelines
EMBO Reports - Author Guidelines
Molecular Systems Biology - Author Guidelines
EMBO Molecular Medicine - Author Guidelines

Reporting Checklist for Life Science Articles (updated January 2022)
This checklist is adapted from Materials Design Analysis Reporting (MDAR) Checklist for Authors. MDAR establishes a minimum set of requirements in transparent reporting in the life sciences (see Statement of Task: 10.31222/osf.io/9sm4x). Please follow the journal's guidelines in preparing your manuscript.
Please note that a copy of this checklist will be published alongside your article.

Abridged guidelines for figures
1. Data
The data shown in figures should satisfy the following conditions:
- the data were obtained and processed according to the field's best practice and are presented to reflect the results of the experiments in an accurate and unbiased manner.
- ideally, figure panels should include only measurements that are directly comparable to each other and obtained with the same assay.
- plots include clearly labeled error bars for independent experiments and sample sizes. Unless justified, error bars should not be shown for technical replicates.
- if n<5, the individual data points from each experiment should be plotted. Any statistical test employed should be justified.
- Source Data should be included to report the data underlying figures according to the guidelines set out in the authorship guidelines on Data Presentation.

2. Captions
Each figure caption should contain the following information, for each panel where they are relevant:
- a specification of the experimental system investigated (eg cell line, species name).
- the assay(s) and method(s) used to carry out the reported observations and measurements.
- an explicit mention of the biological and chemical entity(ies) that are being measured.
- an explicit mention of the biological and chemical entity(ies) that are altered/varied/perturbed in a controlled manner.
- the exact sample size (n) for each experimental group/condition, given as a number, not a range;
- a description of the sample collection allowing the reader to understand whether the samples represent technical or biological replicates (including how many animals, litters, cultures, etc.).
- a statement of how many times the experiment shown was independently replicated in the laboratory.
- definitions of statistical methods and measures:
  - common tests, such as t-test (please specify whether paired vs. unpaired), simple χ2 tests, Wilcoxon and Mann-Whitney tests, can be unambiguously identified by name only, but more complex techniques should be described in the methods section;
  - are tests one-sided or two-sided?
  - are there adjustments for multiple comparisons?
  - exact statistical test results, e.g., P values = x but not P values < x;
  - definition of 'center values' as median or average;
  - definition of error bars as s.d. or s.e.m.

Please complete ALL of the questions below.
Select "Not Applicable" only when the requested information is not relevant for your study.

Materials

Newly Created Materials	Information included in the manuscript?	In which section is the information available? (Reagents and Tools Table, Materials and Methods, Figures, Data Availability Section)
New materials and reagents need to be available; do any restrictions apply?	Not Applicable	

Antibodies	Information included in the manuscript?	In which section is the information available? (Reagents and Tools Table, Materials and Methods, Figures, Data Availability Section)
For **antibodies** provide the following information:<br>- Commercial antibodies: RRID (if possible) or supplier name, catalogue number and or/clone number<br>- Non-commercial: RRID or citation	Not Applicable	

DNA and RNA sequences	Information included in the manuscript?	In which section is the information available? (Reagents and Tools Table, Materials and Methods, Figures, Data Availability Section)
**Short novel DNA or RNA including primers, probes**: provide the sequences.	Not Applicable	

Cell materials	Information included in the manuscript?	In which section is the information available? (Reagents and Tools Table, Materials and Methods, Figures, Data Availability Section)
**Cell lines:** Provide species information, strain. Provide accession number in repository **OR** supplier name, catalog number, clone number, and/**OR** RRID.	Not Applicable	
**Primary cultures:** Provide species, strain, sex of origin, genetic modification status.	Not Applicable	
Report if the cell lines were recently **authenticated** (e.g., by STR profiling) and tested for mycoplasma contamination.	Not Applicable	

Experimental animals	Information included in the manuscript?	In which section is the information available? (Reagents and Tools Table, Materials and Methods, Figures, Data Availability Section)
**Laboratory animals or Model organisms:** Provide species, strain, sex, age, genetic modification status. Provide accession number in repository **OR** supplier name, catalog number, clone number, **OR** RRID.	Not Applicable	
**Animal observed in or captured from the field:** Provide species, sex, and age where possible.	Not Applicable	
Please detail **housing and husbandry conditions**.	Not Applicable	

Plants and microbes	Information included in the manuscript?	In which section is the information available? (Reagents and Tools Table, Materials and Methods, Figures, Data Availability Section)
**Plants:** provide species and strain, ecotype and cultivar where relevant, unique accession number if available, and source (including location for collected wild specimens).	Not Applicable	
**Microbes:** provide species and strain, unique accession number if available, and source.	Not Applicable	

Human research participants	Information included in the manuscript?	In which section is the information available? (Reagents and Tools Table, Materials and Methods, Figures, Data Availability Section)
If collected and within the bounds of privacy constraints report on age, sex and gender or ethnicity for all study participants.	Not Applicable	

Core facilities	Information included in the manuscript?	In which section is the information available? (Reagents and Tools Table, Materials and Methods, Figures, Data Availability Section)
If your work benefited from core facilities, was their service mentioned in the acknowledgments section?	Yes	Acknowledgments

Design

Study protocol	Information included in the manuscript?	In which section is the information available? (Reagents and Tools Table, Materials and Methods, Figures, Data Availability Section)
If study protocol has been **pre-registered, provide DOI in the manuscript**. For clinical trials, provide the trial registration number **OR** cite DOI.	Not Applicable	
Report the **clinical trial registration number** (at ClinicalTrials.gov or equivalent), where applicable.	Not Applicable	

Laboratory protocol	Information included in the manuscript?	In which section is the information available? (Reagents and Tools Table, Materials and Methods, Figures, Data Availability Section)
Provide DOI OR other citation details if **external detailed step-by-step protocols** are available.	Not Applicable	

Experimental study design and statistics	Information included in the manuscript?	In which section is the information available? (Reagents and Tools Table, Materials and Methods, Figures, Data Availability Section)
Include a statement about **sample size** estimate even if no statistical methods were used.	Not Applicable	
Were any steps taken to minimize the effects of subjective bias when allocating animals/samples to treatment (e.g. **randomization procedure**)? If yes, have they been described?	Not Applicable	
Include a statement about **blinding** even if no blinding was done.	Not Applicable	
Describe **inclusion/exclusion criteria** if samples or animals were excluded from the analysis. Were the criteria pre-established?<br><br>If sample or data points were omitted from analysis, report if this was due to attrition or intentional exclusion and provide justification.	Not Applicable	
For every figure, are **statistical tests** justified as appropriate? Do the data meet the assumptions of the tests (e.g., normal distribution)? Describe any methods used to assess it. Is there an estimate of variation within each group of data? Is the variance similar between the groups that are being statistically compared?	Yes	Figures, Methods

Sample definition and in-laboratory replication	Information included in the manuscript?	In which section is the information available? (Reagents and Tools Table, Materials and Methods, Figures, Data Availability Section)
In the figure legends: state number of times the experiment was **replicated** in laboratory.	Yes	Figures legends
In the figure legends: define whether data describe **technical or biological replicates**.	Not Applicable	

Ethics

Ethics	Information included in the manuscript?	In which section is the information available? (Reagents and Tools Table, Materials and Methods, Figures, Data Availability Section)
Studies involving **human participants**: State details of **authority granting ethics approval** (IRB or equivalent committee(s), provide reference number for approval.	Not Applicable	
Studies involving **human participants**: Include a statement confirming that **informed consent** was obtained from all subjects and that the experiments conformed to the principles set out in the WMA Declaration of Helsinki and the Department of Health and Human Services Belmont Report.	Not Applicable	
Studies involving **human participants:** For publication of **patient photos**, include a statement confirming that consent to publish was obtained.	Not Applicable	
Studies involving experimental **animals**: State details of **authority granting ethics approva**l (IRB or equivalent committee(s), provide reference number for approval. Include a statement of compliance with ethical regulations.	Not Applicable	
Studies involving **specimen and field samples:** State if relevant **permits** obtained, provide details of authority approving study; if none were required, explain why.	Not Applicable	

Dual Use Research of Concern (DURC)	Information included in the manuscript?	In which section is the information available? (Reagents and Tools Table, Materials and Methods, Figures, Data Availability Section)
Could your study fall under dual use research restrictions? Please check biosecurity documents and list of **select agents and toxins** (CDC): https://www.selectagents.gov/sat/list.htm	Not Applicable	
If you used a select agent, is the security level of the lab appropriate and reported in the manuscript?	Not Applicable	
If a study is subject to dual use research of concern regulations, is the name of the **authority granting approval and reference number** for the regulatory approval provided in the manuscript?	Not Applicable	

Reporting

The MDAR framework recommends adoption of discipline-specific guidelines, established and endorsed through community initiatives. Journals have their own policy about requiring specific guidelines and recommendations to complement MDAR.

Adherence to community standards	Information included in the manuscript?	In which section is the information available? (Reagents and Tools Table, Materials and Methods, Figures, Data Availability Section)
State if relevant guidelines or checklists (e.g., **ICMJE, MIBBI, ARRIVE, PRISMA**) have been followed or provided.	Not Applicable	
For **tumor marker prognostic studies,** we recommend that you follow the **REMARK** reporting guidelines (see link list at top right). See author guidelines, under 'Reporting Guidelines'. Please confirm you have followed these guidelines.	Not Applicable	
For **phase II and III randomized controlled trials**, please refer to the **CONSORT** flow diagram (see link list at top right) and submit the CONSORT checklist (see link list at top right) with your submission. See author guidelines, under 'Reporting Guidelines'. Please confirm you have submitted this list.	Not Applicable	

Data Availability

Data availability	Information included in the manuscript?	In which section is the information available? (Reagents and Tools Table, Materials and Methods, Figures, Data Availability Section)
Have **primary datasets** been deposited according to the journal's guidelines (see 'Data Deposition' section) and the respective accession numbers provided in the Data Availability Section?	Not Applicable	
Were **human clinical and genomic datasets** deposited in a public access-controlled repository in accordance to ethical obligations to the patients and to the applicable consent agreement?	Not Applicable	
Are **computational models** that are central and integral to a study available without restrictions in a machine-readable form? Were the relevant accession numbers or links provided?	Not Applicable	
If publicly available data were reused, provide the respective **data citations in the reference list**.	Not Applicable	
